# Synthesis, computational studies, antimycobacterial and antibacterial properties of pyrazinoic acid–isoniazid hybrid conjugates†

**DOI:** 10.1039/c9ra03380g

**Published:** 2019-07-01

**Authors:** Siva S. Panda, Adel S. Girgis, Bibhuti B. Mishra, Mohamed Elagawany, Venkatasai Devarapalli, William F. Littlefield, Ahmed Samir, Walid Fayad, Nehmedo G. Fawzy, Aladdin M. Srour, Riham M. Bokhtia

**Affiliations:** Department of Chemistry & Physics, Augusta University Augusta GA 30912 USA sspanda12@gmail.com; Department of Pesticide Chemistry, National Research Centre Dokki Giza 12622 Egypt girgisas10@yahoo.com; Department of Immunology and Microbial Disease, Albany Medical College 47 New Scotland Avenue Albany NY-12208 USA; Department of Pharmaceutical Chemistry, Faculty of Pharmacy, Damanhour University Damanhour Egypt; Microbiology Department, Faculty of Veterinary Medicine, Cairo University Cairo Egypt; Drug Bioassay-Cell Culture Laboratory, Pharmacognosy Department, National Research Centre Dokki Giza 12622 Egypt; Department of Therapeutic Chemistry, National Research Centre Dokki Giza 12622 Egypt; Department of Pharmaceutical Organic Chemistry, Faculty of Pharmacy, Zagazig University Zagazig 44519 Egypt

## Abstract

Benzotriazole and microwave mediated syntheses led to a new set of hybrid conjugates of pyrazinoic acid with isoniazid *via* amino acid linkers in excellent yields with retention of chirality. Microbiological screening of the synthesized conjugates revealed an exceptionally high activity against some of the pathogenic bacterial strains at low concentrations. Promising antimycobacterial properties were observed against tuberculous and non-tuberculous mycobacteria. Robust molecular models (2D-QSAR and 3D-pharmacophore) support the observed biological properties. Safety profile of the synthesized conjugates against human normal cell (RPE1) was evaluated by MTT technique.

## Abstract 2

Synthesis and computational studies of new pyrazinoic acid–isoniazid hybrid conjugates as potential anti-infective agents.

## Graphical abstract

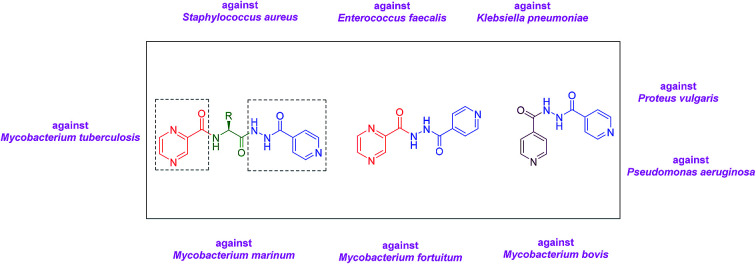

## Introduction

Infectious diseases continue to pose a major threat to human health caused by pathogenic microorganisms, such as viruses, bacteria, fungi or parasites. According to the World Health Organization (WHO), despite all the progress made to control or cure infectious diseases, the major challenges persist in the new drug development with high efficacy/selectivity for these diseases. TB (tuberculosis) is one of the top 10 causes of death worldwide and the leading cause of mortality/morbidity from a single infectious agent (above HIV/AIDS). Millions of people continue to fall sick with TB each year. In 2017, TB caused an estimated 1.3 million deaths (range, 1.2–1.4 million) among HIV-negative people and there were an additional 300 000 deaths from TB (range, 266 000–335 000) among HIV-positive people. Nearly 10.0 million people (range, 9.0–11.1 million) developed TB disease in 2017: among which 5.8 million were men, 3.2 million women and 1.0 million were children (WHO, 2018).^1^

TB disease is caused by a bacterial pathogen *Mycobacterium tuberculosis*, that existed for millennia and continues to be the leading cause of infectious disease deaths globally.^2^ It is known to cause pulmonary infection and become extremely pervasive within the lungs and between subjects. The current directly observed treatment, short course (DOTS) tuberculosis therapy requires a minimum of six months of treatment consisting of two months of the intensive phase of treatment with the first-line drugs including isoniazid (INH), rifampin (RIF), pyrazinamide (PZA) and ethambutol (EMB), followed by another four months of therapy with INH and RIF alone. Among the first line antibiotics, PZA is a highly effective bactericidal drug which is known to penetrate into TB lesions and remain active in the highly acidic environment of the TB granuloma. The parent prodrug is metabolized *via* pyrazinamidase (PZase) to pyrazinoic acid; which is the active form of the drug. However, the exact mechanism of action is not clear. Like PZA, INH is also a prodrug that requires the activity of mycobacterial catalase KatG. This interaction activates a series of reactions that eventually lead to the inhibition of mycolic acid synthesis, building block of mycobacterial cell wall. Inappropriate treatment, poor drug quality, and inadequate drug intake or treatment, all contribute to the emergence of drug resistant bacterial strains classified as multidrug-resistant (MDR) and extremely drug resistant (XDR) strains.^3^ Currently, existing drugs are of limited efficacy against drug resistance TB strains besides their known side effects.^4^

*Mycobacterium bovis* is another *Mycobacterium* strain that causes TB disease in animals and humans. In addition to *Mycobacterium tuberculosis* and *Mycobacterium bovis*, there are many non-tuberculous mycobacteria like *Mycobacterium marinum* and *Mycobacterium fortuitum* also cause various infections in humans. Although many therapeutic agents were developed for non-tuberculous mycobacterial infections, no standard treatment protocol was approved due to their natural multi-drug resistance.^5^

Drug resistance is a major global health concern for a plethora of bacterial and fungal infections.^6^ Gram-positive and Gram-negative bacterial strains are involved in the majority of infectious diseases.^7,8^ The rapid emergence of MDR infections necessitates the design and synthesis of novel antimicrobial molecules. The development of novel antimicrobial drugs is key to the treatment of infectious diseases, especially for which combinatorial drug treatments are needed.

Sub-optimal therapy caused by inefficient cellular penetration is a major issue associated with drug resistance. Many therapeutic agents owing to their charge, hydrophilic property, and/or size and stability under physiological conditions are not suitable for treating intracellular bacterial infections that require the drug to penetrate the cell membrane of the host and also the pathogen. To compensate for the reduced penetration and bioavailability, high drug doses are often used that causes strong adverse effects for vital organs. Synthetic vectors can offer a solution for these problems associated with drug delivery, overcoming the limitations such as bioavailability and/or cytotoxicity. Among such synthetic vectors, cell-penetrating peptides and amino acids have been successfully utilized because of their drug carrier ability across cell membranes.^9^ Recently, several approaches have been reported to combat the MDR problem.^10–13^

The present study deals with the synthesis of novel pyrazinoic acid–isoniazid conjugates connected through an amino acid linker using molecular hybridization approach and investigating their properties against a variety of microbial strains including, aerobic bacteria, tuberculous and non-tuberculous mycobacteria. Conjugation of effective drug fragments for developing novel agents is a well successful approach in drug design.^14^ Microwave methodology is utilized to synthesize the targeted conjugates since microwave protocol is well known in medicinal chemistry for synthesizing drug like molecules in high yield and purity.^15^ Molecular modeling techniques are effective tools for understanding and validation of the biological data.

## Results and discussion

### Chemistry

**Scheme 1:**
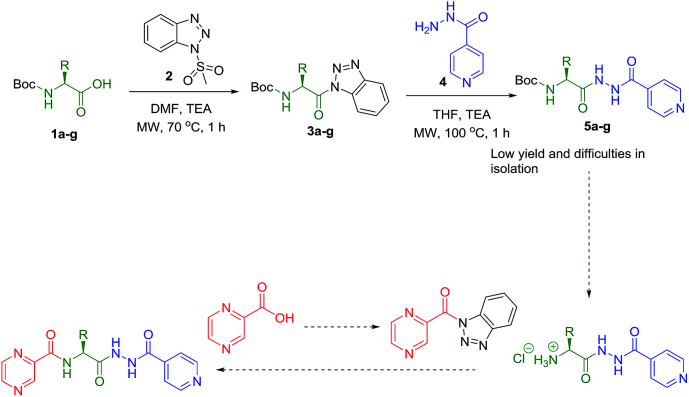
Synthesis of POA hybrids through reaction of isoniazid and amino acid (Route I).

Synthesis of the targeted hybrids was initially studied by activating the Boc-protected amino acids (1a–g) with 1*H*-benzotriazole. The boc-protected aminoacyl benzotriazoles (3a–g) were treated with isoniazid (4) in the presence of trimethylamine in acetonitrile at 20 °C for 2 h. According to the TLC profile, the reaction was completed but the formed compounds 5a–g could not be isolated in good yield because of the hydrophilic nature of isoniazid (4) (Scheme 1). Different conditions were utilized but none of them was successful to isolate the conjugates 5a–g in good yields.

**Scheme 2:**
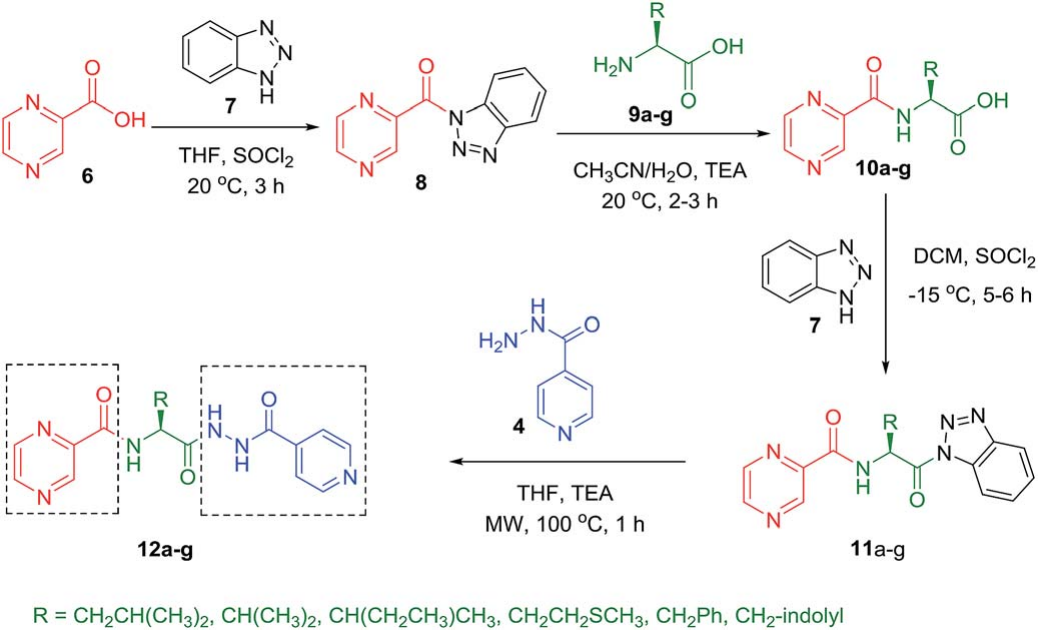
Synthesis of POA hybrids through reaction of POA–aminoacyl benzotriazoles with isoniazid (Route II).

Slight modification was considered for the synthetic strategy, where the pyrazinoic acid (POA, 6) was activated as benzotriazolyl derivative 8 obeying the reported procedure^16^ and coupled with free amino acids (9a–g) in a mixture of acetonitrile and water (7 : 3) at 20 °C for 2 h in the presence of 1.5 equivalents of trimethylamine to give compounds 10a–g. In our previous communication,^14^ we reported that we failed to prepare benzotriazole derivatives of POA–amino acid conjugates 10a–g. This time we were able to optimize the reaction condition and prepare the POA–aminoacyl benzotriazoles (11a–g) in pure form by treating 10a–g with benzotriazole (7) in presence of thionyl chloride at −15 °C for 5–6 h. Compounds 11a–g were irradiated under microwave condition with 4 in the presence of trimethylamine at 100 °C for 1 h to obtain our desired conjugates 12a–g in good yields without losing the chiral integrity (Scheme 2). The confirmation of chiral integrity was established by HPLC studies by comparing the spectra of l-phenylalanine and dl-phenylalanine containing conjugates.

**Scheme 3:**
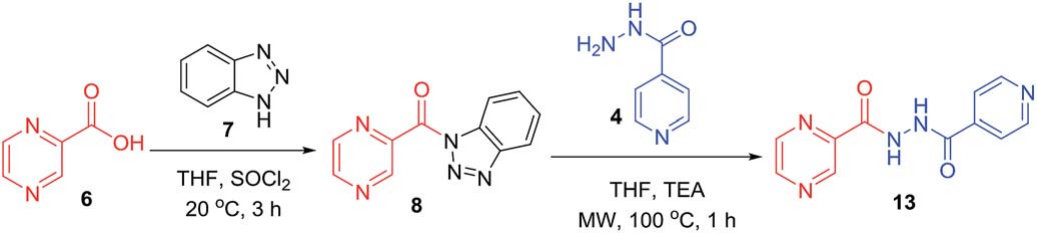
Synthesis of POA-INH hybrid.

**Scheme 4:**
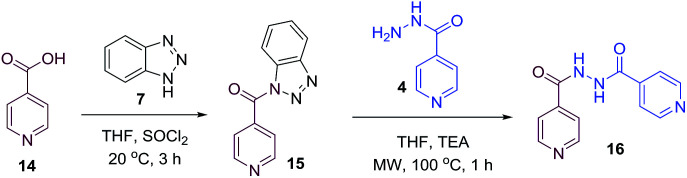
Synthesis of INH bis-conjugate.

To better understand the role and effect of amino acids in biological properties, we have also synthesized hybrid of pyrazinoic acid and isoniazid 13 without any linker (Scheme 3). As isoniazid is the pro-drug of isonicotinic acid, we prepared the bis conjugate of isonicotinic acid to compare the biological property of the hybrid 13 and bis conjugate 16 of isoniazid (Scheme 4). Conjugates 13 and 16 were previous reported and synthesized by using various catalysts and reagents.^17–20^ However, we were able to complete the synthesis by utilizing the aforementioned benzotriazole methodology under microwave irradiation in good yield and purity.

### Biological studies

#### Aerobic antibacterial properties

Antibacterial properties were investigated for the synthesized compounds along with parent drugs pyrazinamide (PZA), isoniazid (INH) and a standard drug ciprofloxacin against a variety of Gram-positive (*Staphylococcus aureus*, *Enterococcus faecalis*) and Gram-negative (*Klebsiella pneumonia*, *Proteus vulgaris*, *Pseudomonas aeruginosa*) clinical isolate bacteria utilizing the standard technique.^21^ From the results obtained (Table 1) the following observations can be stated.

**Table 1:** Antimicrobial properties of the tested compounds against aerobic bacteria

Entry	Compd	Minimum inhibitory concentration (MIC), μg mL^−1^ (μM)				
		*Staphylococcus aureus*	*Enterococcus faecalis*	*Klebsiella pneumoniae*	*Proteus vulgaris*	*Pseudomonas aeruginosa*
1	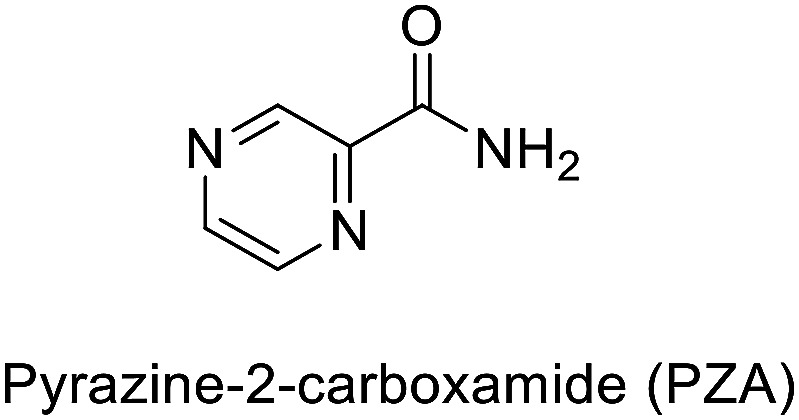	1024 (8317.43)	0.12 (0.97)	64 (519.84)	8 (64.98)	4 (32.49)
2	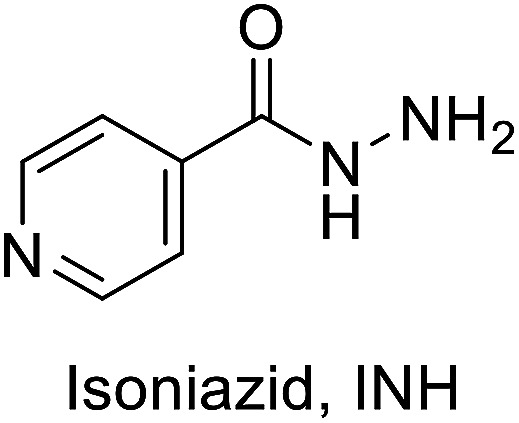	64 (466.67)	16 (116.67)	8 (58.33)	64 (466.67)	64 (466.67)
3	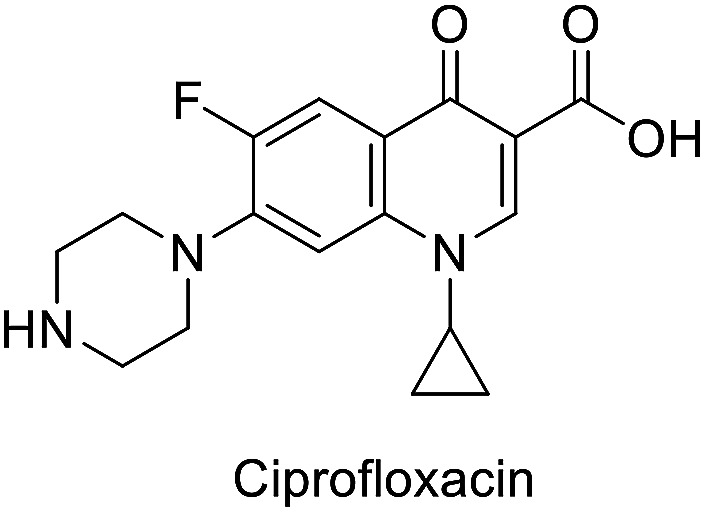	0.4 (1.21)	0.4 (1.21)	2 (6.04)	2 (6.04)	4 (12.07)
4	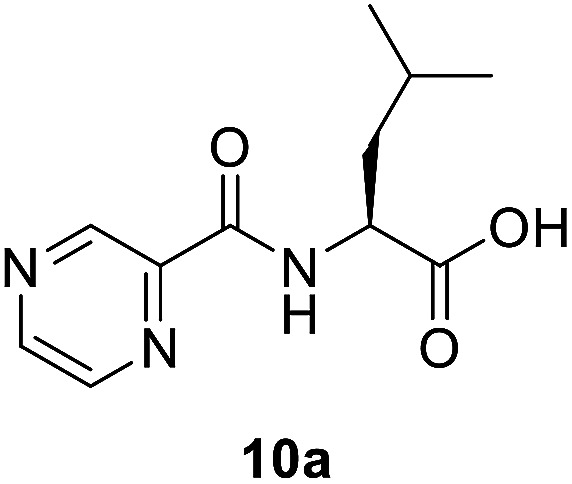	1024 (4315.96)	0.03 (0.13)	64 (269.75)	8 (33.72)	0.03 (0.13)
5	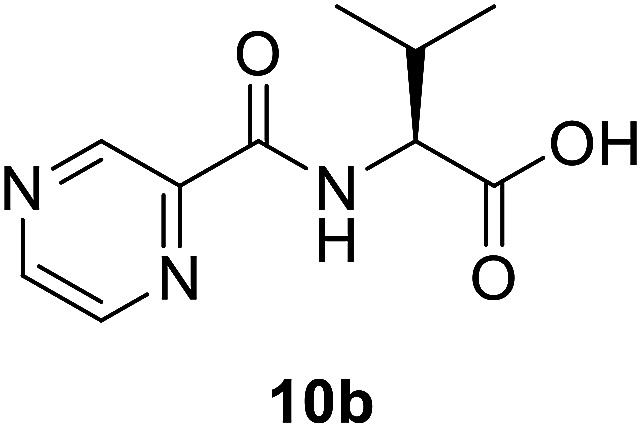	512 (2293.58)	0.03 (0.13)	0.03 (0.13)	0.06 (0.27)	4 (17.92)
6	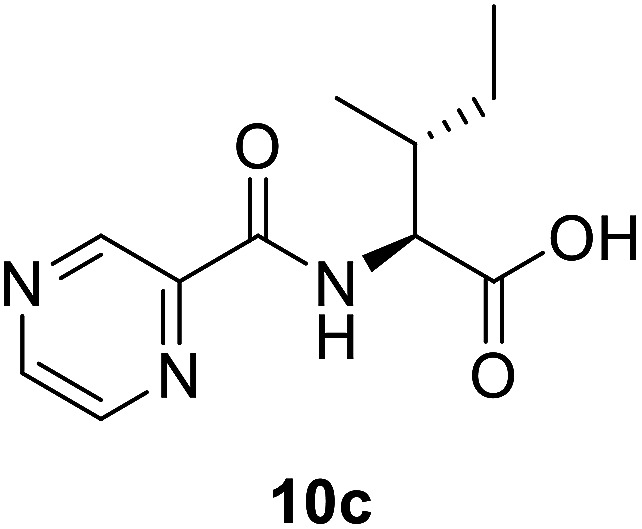	16 (67.44)	0.03 (0.13)	8 (33.72)	0.06 (0.25)	0.12 (0.51)
7	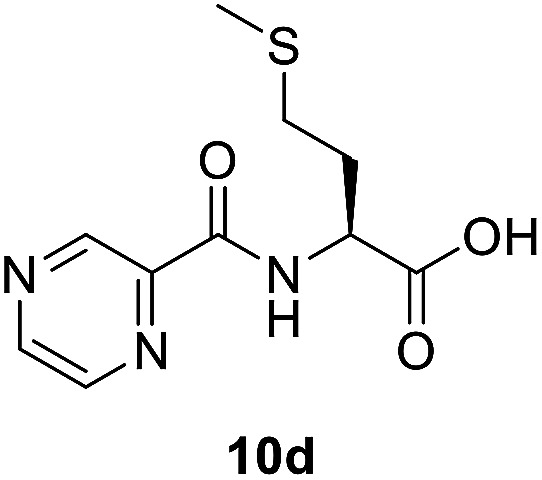	16 (62.67)	0.03 (0.12)	64 (250.69)	0.06 (0.24)	0.12 (0.47)
8	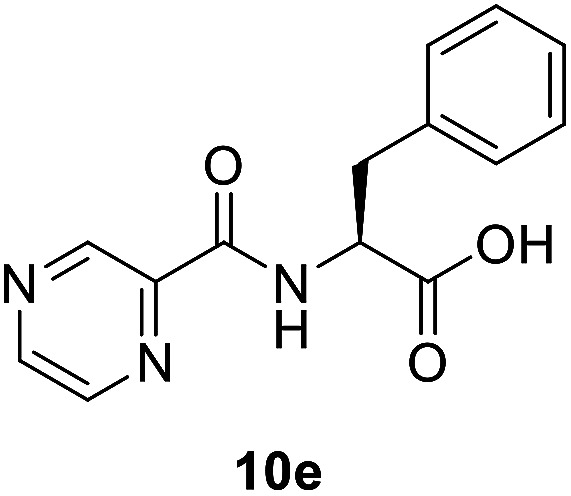	64 (235.92)	0.12 (0.44)	64 (235.92)	0.06 (0.22)	0.12 (0.44)
9	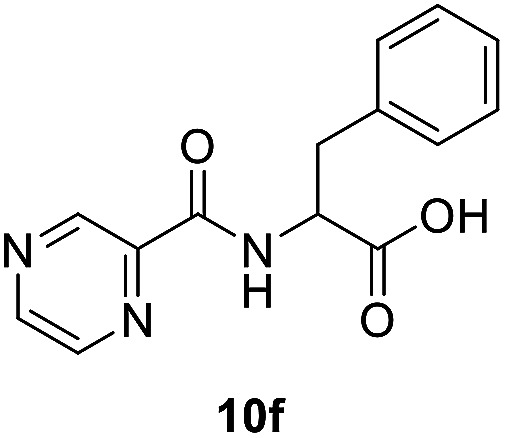	16 (58.98)	0.03 (0.11)	16 (58.98)	0.06 (0.22)	16 (58.98)
10	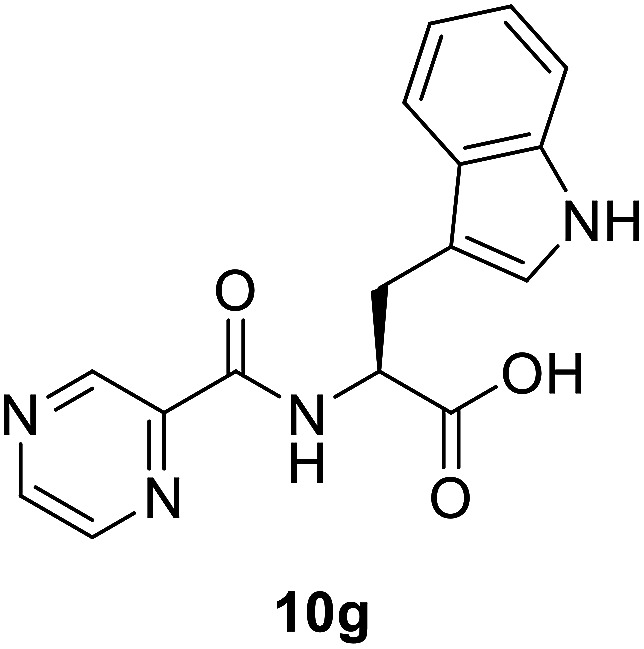	64 (206.24)	0.03 (0.10)	64 (206.24)	0.06 (0.19)	0.12 (0.39)
11	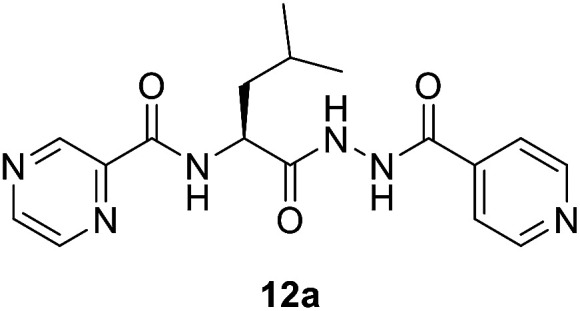	2 (5.61)	0.03 (0.08)	4 (11.22)	0.06 (0.17)	64 (179.58)
12	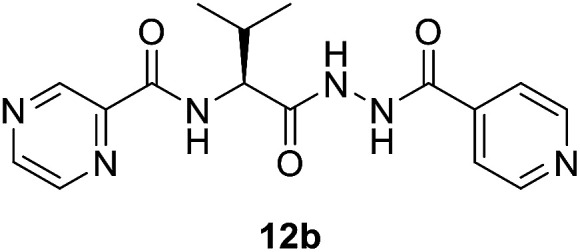	16 (46.73)	0.12 (0.35)	8 (23.37)	0.06 (0.18)	0.12 (0.35)
13	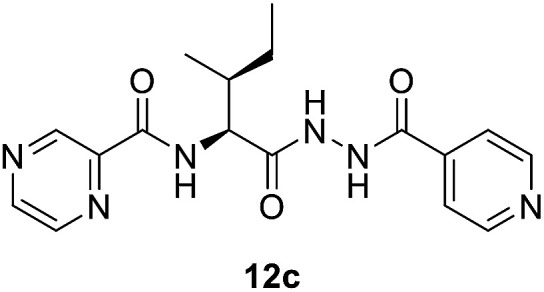	16 (44.90)	0.03 (0.08)	8 (22.45)	0.06 (0.17)	0.12 (0.34)
14	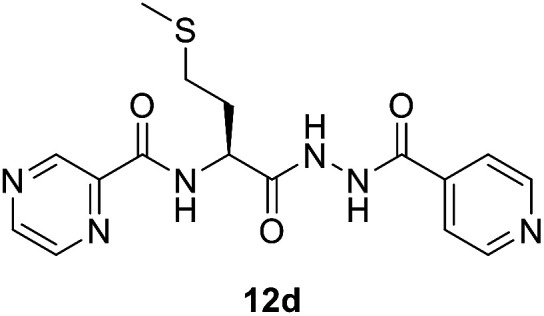	64 (170.93)	0.03 (0.08)	4 (10.68)	0.06 (0.16)	0.12 (0.32)
15	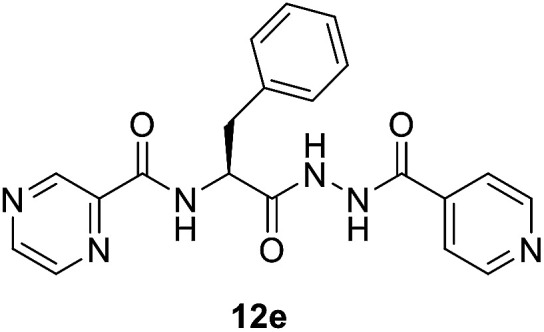	16 (40.98)	0.12 (0.31)	8 (20.49)	0.06 (0.15)	0.12 (0.31)
16	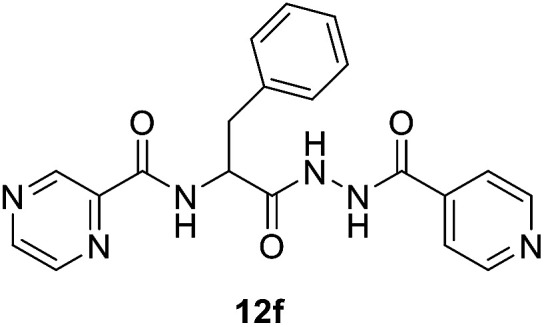	16 (40.98)	0.03 (0.08)	8 (20.49)	16 (40.98)	32 (81.97)
17	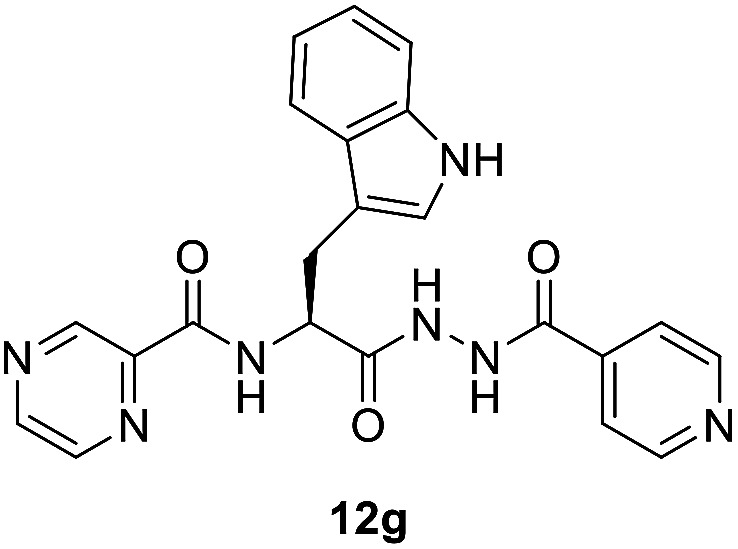	16 (37.26)	0.03 (0.07)	4 (9.31)	0.06 (0.14)	0.12 (0.28)
18	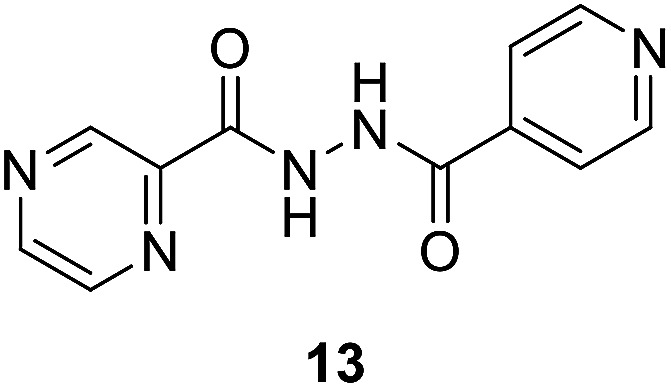	8 (32.89)	16 (65.78)	8 (32.89)	64 (263.13)	0.03 (0.12)
19	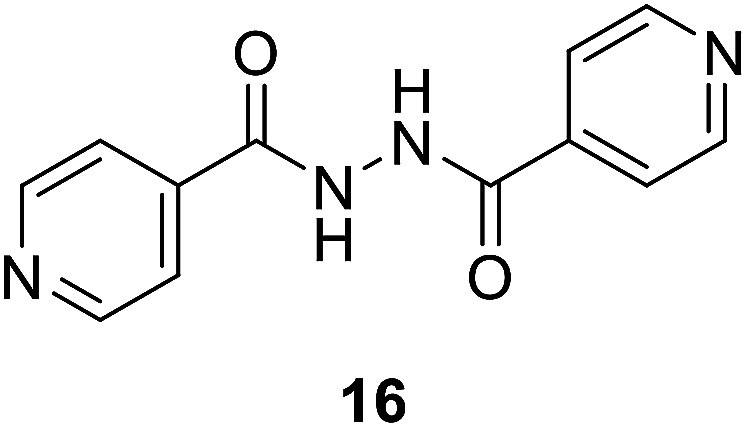	4 (16.51)	16 (66.05)	4 (16.51)	0.03 (0.12)	0.06 (0.25)

All the tested conjugates (10a–g and 12a–g) show high antibacterial properties at sub-micromolar values (MIC = 0.44–0.07 μM) with potency higher than that of the standard reference used (MIC of ciprofloxacin = 1.21 μM) against *Enterococcus faecalis*. Similar observations were also shown for most of the tested conjugates against *Proteus vulgaris* (except compounds 10a and 12f) and *Pseudomonas aeruginosa* (except compounds 10b, 10f, and 12a) with MIC values = 0.14–0.27, 0.13–0.51 μM, respectively relative to the standard reference ciprofloxacin (MIC = 6.04, 12.07 μM, respectively). It has also been noticed that all the synthesized conjugates 12a–g show higher potency than their precursors 10a–g against *Klebsiella pneumonia*. Similar inhibition properties were noticed towards *Enterococcus faecalis* [compounds 12b is an exception revealing antimicrobial properties close to its precursor 10b (MIC = 0.33, 0.13 μM, respectively)].

Establishing SAR (structure–activity relationships) with this information is difficult. However, we noticed some evidence like utilization of tryptophan enhances the antimicrobial properties against all the tested microorganisms than phenylalanine as observed for the synthesized compounds 10g/12g*versus*10e/12e. This can be attributed to the effect of indolyl heterocycle as indole analogues are well known for potential activities. The synthesized bis-conjugate 16 seems higher antimicrobial agent against *S. aureus*, *K. pneumonia*, and *P. vulgaris* than the hybrid analog 13 (MIC = 16.51, 16.51, 0.12; 32.89, 32.89, 263.13 μM, respectively). It also reveals close property to that of 13 against *E. faecalis* (MIC = 65.78, 66.05 μM for compounds 13 and 16, respectively). Overall hybrid conjugates with amino acids are more active than the hybrid conjugates without amino acid as a linker. In our future studies, it is intended to explore more possible linkers as well as use dipeptides and tripeptides to establish an evidence-based SAR and to develop more potent antibacterial agents.

#### Antimycobacterial properties

**Fig. 1:**
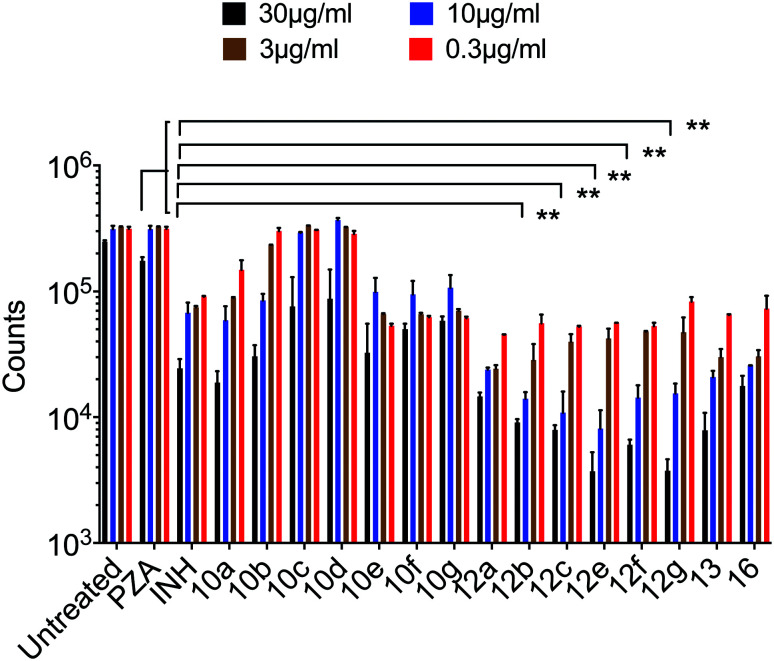
Bacterial growth was measured by recording luciferase counts in an IVIS-spectrum imaging system (data are presented as mean ± SD), one-way ANOVA Tukey's multiple comparison test. **, *p* < 0.001.

**Fig. 2:**
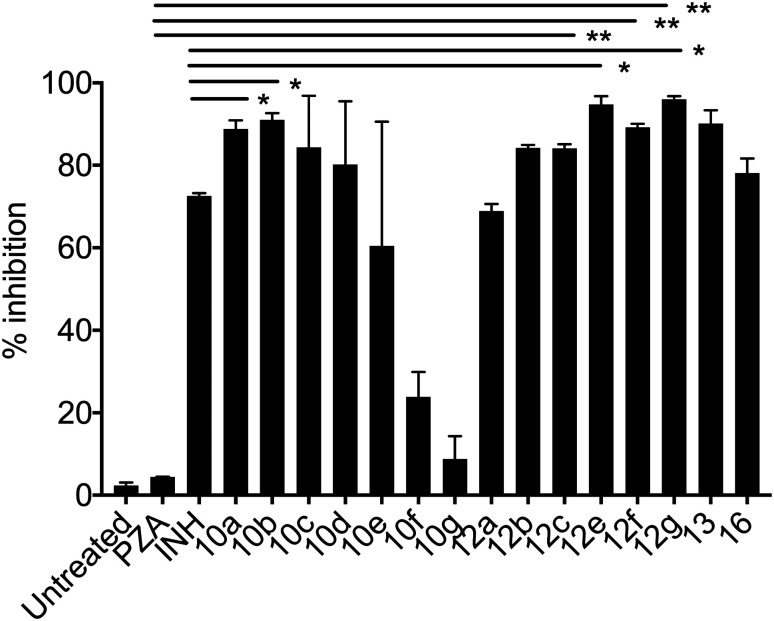
% growth inhibition was shown compared to untreated controls (data are presented as mean ± SD). Student's two-tailed *t* test. *, *p* < 0.05; **, *p* < 0.001.

Antimycobacterial properties of the synthesized compounds 10a–g, 12a–g, 13 and 16 were screened against clinical isolate tuberculous (*Mycobacterium bovis*) and non-tuberculous (*Mycobacterium marinum*, *Mycobacterium fortuitum*) strains using the standard technique.^16,22^ Antituberculosis properties of the conjugates were also studied against highly virulent *Mycobacterium tuberculosis* at Albany Medical College, Albany, NY. All the experimental data were compared with the reference standards PZA, INH. Based on the mycobacterial observations (Table 2, Fig. 1 and 2), the following SAR are observed.

**Table 2:** Anti-mycobacterial properties of the tested compounds

Entry	Compd	Minimum inhibitory concentration (MIC), μg ml^−1^ (μM)		
		*M. marinum*	*M. fortuitum*	*M. bovis*
1	PZA	10 (81.2)	10 (81.2)	20 (162.4)
2	INH	10 (72.9)	20 (145.8)	20 (145.8)
3	10a	20 (84.3)	20 (84.3)	>20 (>84.3)
4	10b	>20 (>89.6)	>20 (>89.6)	>20 (>89.6)
5	10c	>20 (>84.3)	>20 (>84.3)	>20 (>84.3)
6	10d	>20 (>78.3)	>20 (>78.3)	>20 (>78.3)
7	10e	>20 (>73.7)	>20 (>73.7)	>20 (>73.7)
8	10f	20 (73.7)	>20 (>73.7)	>20 (>73.7)
8	10g	>20 (>64.5)	>20 (>64.5)	>20 (>64.5)
10	12a	20 (56.1)	20 (56.1)	>20 (>56.1)
11	12b	20 (58.4)	20 (58.4)	>20 (>58.4)
12	12c	20 (56.1)	20 (56.1)	>20 (>56.1)
13	12d	10 (26.7)	20 (53.4)	>20 (>53.4)
14	12e	20 (51.2)	20 (51.2)	>20 (>51.2)
15	12f	10 (25.6)	10 (25.6)	>20 (>51.2)
16	12g	20 (46.6)	20 (46.6)	>20 (>46.6)
17	13	10 (41.1)	10 (41.1)	20 (82.2)
18	16	20 (82.6)	20 (82.6)	20 (82.6)

##### Mycobacterium marinum

Some of the synthesized conjugates reveal high anti-mycobacterial properties against *M. marinum* (about three folds potency; MIC = 26.7, 25.6 μM of 12d and 12f, respectively) than the standard references used (MIC = 81.2, 72.9 μM of PZA, INH, respectively). Other conjugates synthesized show potency comparable (slight enhancement) to that of the standard references (MIC = 46.6–58.4 μM for conjugates 12a–c, 12e and 12g). Compounds 10a and 10f show biological properties (MIC = 84.3, 73.7 μM, respectively) close to that of the standard references used. The synthesized conjugates 12a–g are more potent than their corresponding intermediates 10a–g. The hybrid 13 (MIC = 41.1 μM) seems an effective agent than its precursors (PZA and NIH) and the bis-conjugate 16 (MIC = 82.6 μM). The data clearly indicates the role of amino acids in the hybrid conjugates and probably synergetic effect of the two parent molecules. Interestingly, the aromatic amino acid residues show enhanced activity than the others.

##### Mycobacterium fortuitum

Compound 12f seems superior among all the synthesized analogs (MIC = 25.6 μM) with a potency about 5.6, 3.2 folds than that of the standard references used (MIC = 145.8, 81.2 μM of INH, PZA, respectively). Many of the synthesized compounds (MIC = 46.6–58.4 μM of conjugates 12a–e and 12g) also reveal promising biological properties relative to the standards. All the synthesized conjugates 12a–g are of high potency than their corresponding intermediates 10a–g. Hybrid conjugate 13 is of higher potency than the bis-conjugate 16. Again we observed tryptophan plays some important role in enhancing the activity.

##### Mycobacterium bovis

Only compounds 13 and 16 (MIC = 82.2, 82.6 μM, respectively) show promising antimycobacterial properties (about 1.8–2.0 folds) than the standard references (MIC = 145.8, 162.4 μM of INH, PZA, respectively). The results indicate the hybrid conjugates with amino acid linker lost the activity.

##### Mycobacterium tuberculosis

The PZA activity was not observed at 30 μg mL^−1^ concentration at pH = 7.0. However, at this concentration, significant growth inhibition was observed for the synthesized conjugates even though the parent PZA is known to exhibit *M. tuberculosis* activity *in vitro* at acidic pH < 6.0. INH showed >70% growth inhibition at 30 μg mL^−1^ concentration, while compound 13 hybrid compounds exhibit same growth inhibition at concentration as low as 3 μg mL^−1^. Moreover, the hybrid conjugates 12a–g exhibited >95% growth inhibitory activity at 30 μg mL^−1^ and >80% growth inhibition at 10 μg mL^−1^ concentration. Notably, PZA–INH hybrid compounds exhibit potent bactericidal effect at physiological PH, 7.0 (Fig. 1 and 2). We clearly notice the effect of hybrid conjugates over the corresponding intermediates 10a–g. We believe the combination along with amino acid enhance the activity of the parent drugs.

#### Antiproliferative properties

Antiproliferative properties of the synthesized compounds were screened against RPE1 (normal human immortalized retinal epithelial) cell line to determine the toxicity/selectivity towards normal cell by the standard MTT technique.^23^ The proliferation properties of RPE1 cell lines are 96.4–80.1% at 100 μM suggest that the synthesized conjugates are likely to be safe to the human normal cell (ESI Table S1†).

### Computational studies

#### 2D-QSAR

2D-QSAR (quantitative structure activity relationships) is a successful technique used intensively in medicinal chemistry. It is capable to express the biological properties in terms of mathematical equation(s) of descriptors (physico-chemical) parameters.^24^ The biologically active compounds against *Mycobacterium marinum*, *Mycobacterium fortuitum* and *Mycobacterium tuberculosis* were considered for 2D-QSAR studies utilizing CODESSA-Pro software that allows understanding the parameters governing bio-properties and also validates the bio-observations.

##### Mycobacterium marinum

The two-descriptor model was determined to describe the properties of the biologically active agents against *Mycobacterium marinum* (ESI Table S2†). Maximum e–n attraction for bond C–N is a semi-empirical descriptor with level of significance = −4.762. This descriptor participates negatively in the 2D-QSAR model. In other words, the highest value of the descriptor the more anti-mycobacterial potency of the compound as observed in the high effective agent synthesized 12d (MIC_observed_ = 26.7, MIC_estimated_ = 30.8 μM) relative to 16 (MIC_observed_ = 82.6, MIC_estimated_ = 82.3 μM) that possess descriptor values = 330.7008, 329.2578, respectively (ESI Tables S3 and S4†). Nuclear-electron attraction energy between two given atoms is determined by eqn (1).^25^1
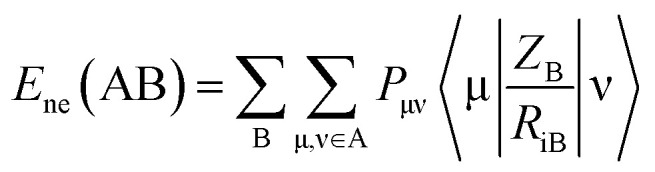
where A, B are two atomic species. *P*_μν_ is the density matrix elements over atomic basis {μν}. *Z*_B_ is the charge of atomic nucleus B, *R*_iB_ is the distance between the electron and atomic nucleus B. 
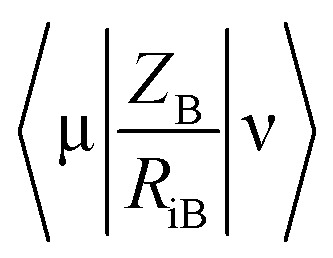
 is the electron-nuclear attraction integrals on atomic basis {μν}.

Maximum e–e repulsion for bond C–C is also a semi-empirical descriptor participates negatively in the BMLR-QSAR model. Electron–electron repulsion between two given atoms can be calculated by eqn (2).^25^2
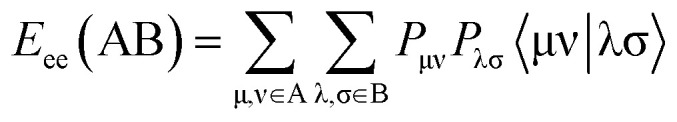
where A, B are two atomic species. *P*_μν_, *P*_λσ_ are the density matrix elements over atomic basis {μνλσ}. 〈μν|λσ〉 is the electron repulsion integrals on atomic basis {μνλσ}.

##### Mycobacterium fortuitum

Two semi-empirical descriptor model is observed for the biologically active agents against *M. fortuitum* (ESI Table S5†). Both of the descriptors positively participate in the BMLR-QSAR model, *i.e.* the higher descriptor value the lower biological properties (ESI Tables S6 and S7†). Electron–electron repulsion energy for a given atomic species can be calculated by eqn (3).^25^3
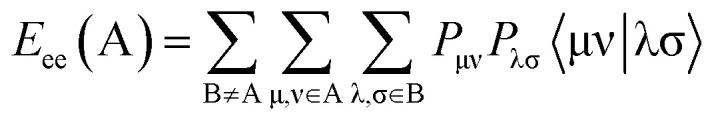
where A, B are two atomic species. *P*_μν_, *P*_λσ_ are the density matrix elements over atomic basis {μνλσ}. 〈μν|λσ〉 is the electron repulsion integrals on atomic basis {μνλσ}.

##### Mycobacterium tuberculosis

Three-descriptor model was observed for the 2D-QSAR study of the tested compounds against *M. tuberculosis* utilizing the log value of % growth inhibition at 30 μg mL^−1^ (ESI Tables S8–S10†). Minimum e–e repulsion for bond H–N and maximum e–e repulsion for bond H–C (semi-empirical descriptors, *t* = 9.543, 7.207, respectively) can be determined by the mentioned eqn (2). Maximum coulombic interaction for bond C–O is also a semi-empirical descriptor (*t* = −14.963). Total interaction energy between two given atomic species can be calculated by eqn (4).^25^

4 *E*_tot_(AB) = *E*_C_(AB) + *E*_exc_(AB)

where, A and B are two atomic species. *E*_C_(AB) and *E*_exc_(AB) are electrostatic interaction and exchange energies between the two atomic species, respectively.

#### Validation of the 2D-QSAR models

**Fig. 3:**
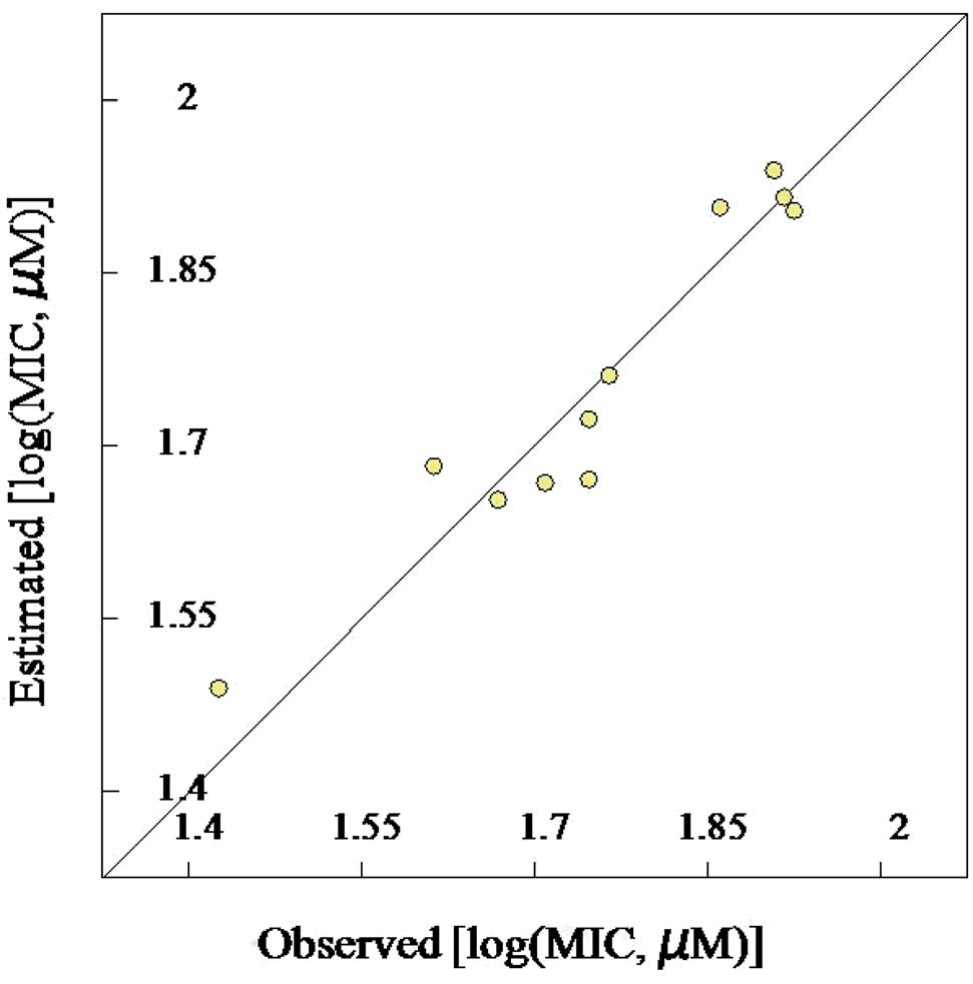
BMLR-QSAR model plot of correlations representing the observed *vs.* predicted log(MIC, μM) values for the tested compounds against *Mycobacterium marinum*.

**Fig. 4:**
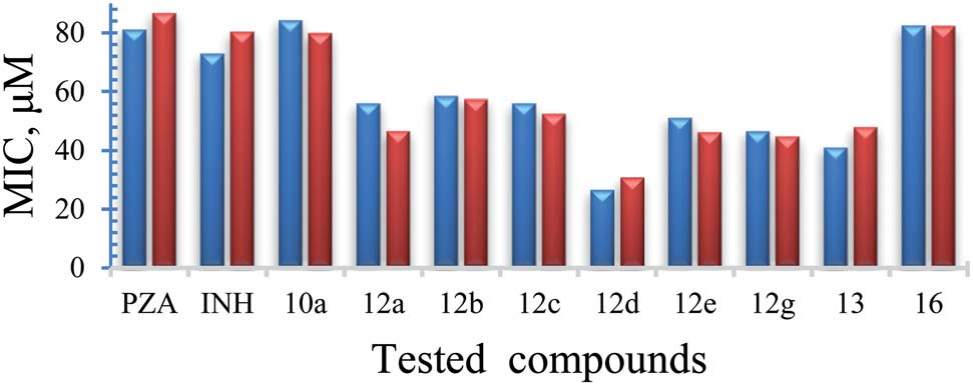
Observed and estimated activity MIC values for the tested compounds against *Mycobacterium marinum* according to the BMLR-QSAR model.

**Fig. 5:**
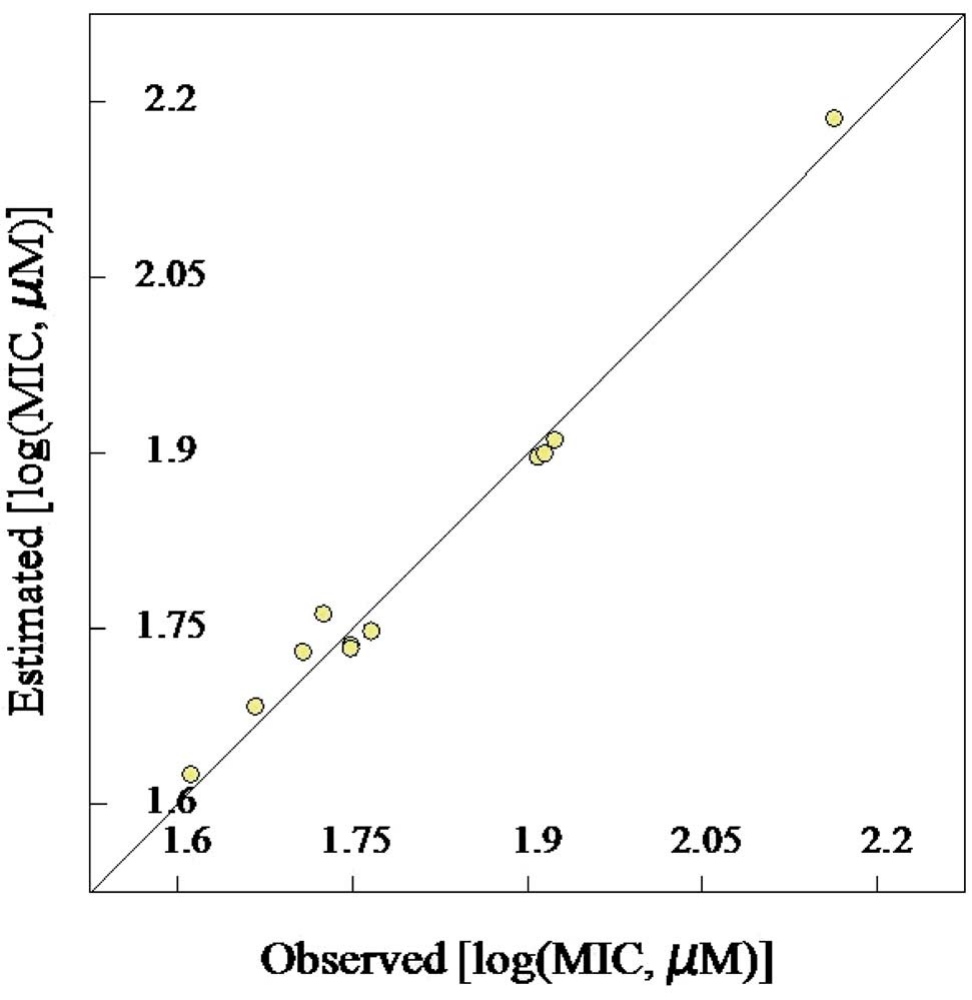
BMLR-QSAR model plot of correlations representing the observed *vs.* predicted log(MIC, μM) values for the tested compounds against *Mycobacterium fortuitum*.

**Fig. 6:**
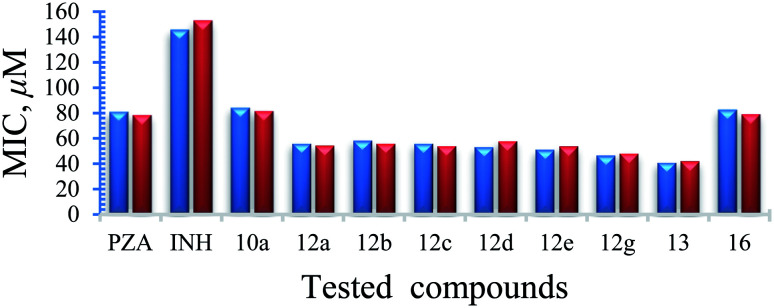
Observed and estimated activity MIC values for the tested compounds against *Mycobacterium fortuitum* according to the BMLR-QSAR model.

**Fig. 7:**
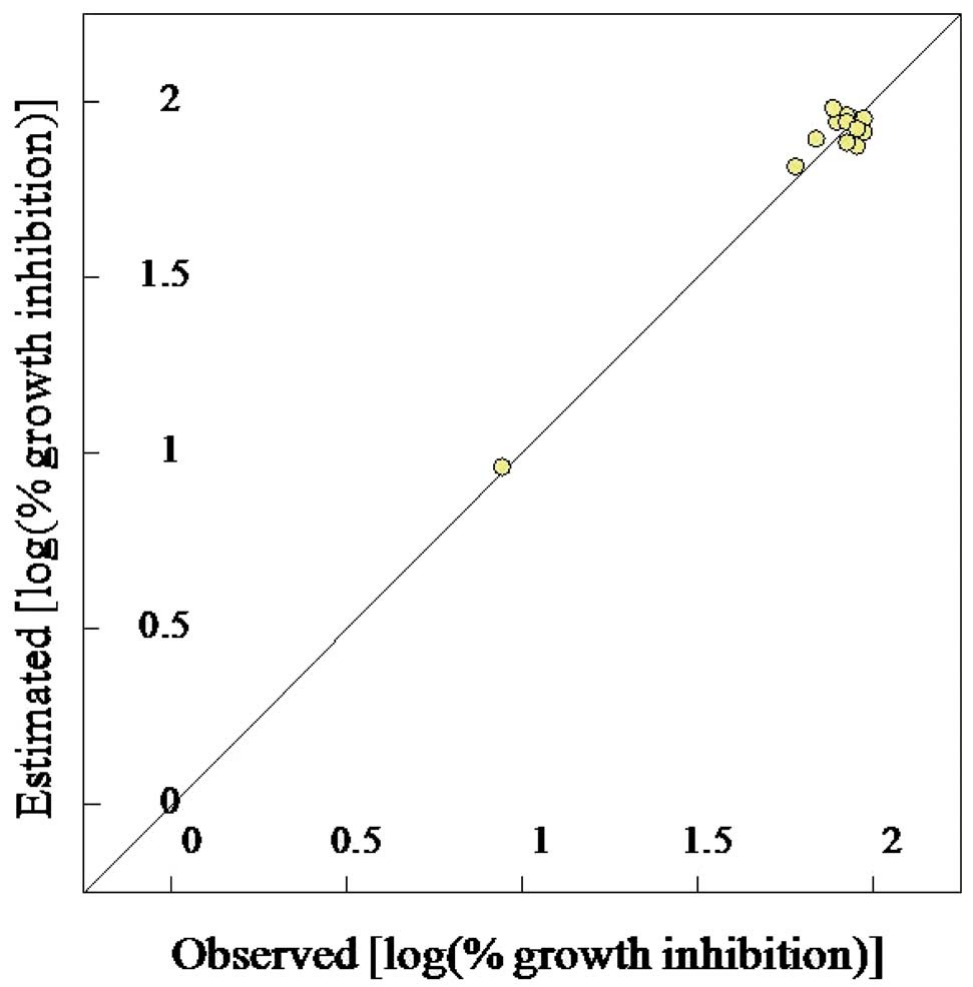
BMLR-QSAR model plot of correlations representing the observed *vs.* predicted log(% growth inhibition at 30 μg mL^−1^) values for the tested compounds against *Mycobacterium tuberculosis*.

**Fig. 8:**
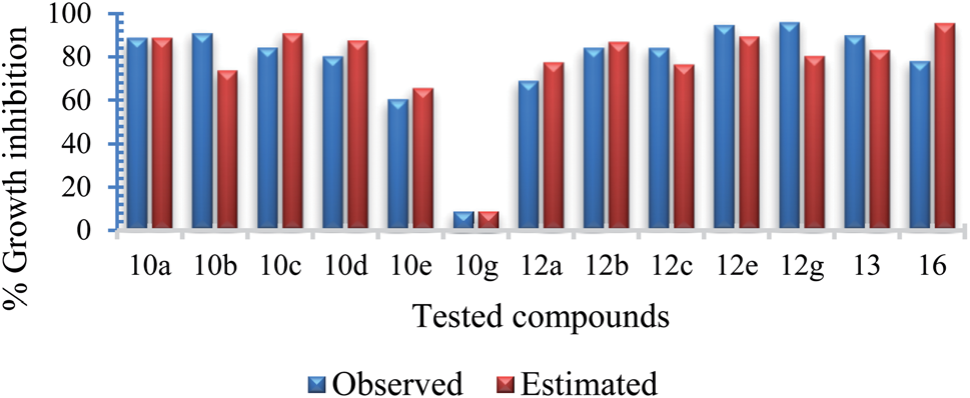
Observed and estimated activity % growth inhibition values at 30 μg mL^−1^ for the tested compounds against *Mycobacterium tuberculosis* according to the BMLR-QSAR model.

Due to the short training set utilized in the 2D-QSAR study, internal validation seems the most appropriate technique.^23^ Goodness of the QSAR models are established by the statistical parameters [including squared correlation coefficient (*R*^2^) and its leave-one-out (*R*^2^cvOO), *F* (Fisher criteria) and *s*^2^ (standard deviation)] (*R*^2^ = 0.908, 0.984, 0.965; *R*^2^cvOO = 0.829, 0.962, 0.892; *F* = 39.543, 240.314, 82.181; and *s*^2^ = 0.003, 0.001, 0.004 for the BMLR-QSAR models of *M. marinum*, *M. fortuitum*, and *M. tuberculosis*, respectively). Estimated properties of the effective agents by the BMLR-QSAR models are comparable to their observed values preserving their potency among each other and to the standard references used (Fig. 3–8; ESI Tables S3, S6 and S9†).

#### 3D-pharmacophore modelling

3D-pharmacophore modeling is an important computational technique explains the biological/pharmacological properties of compound(s) through alignment of the structural elements with chemical features in 3D-array.^26^ The biologically active compounds were undertaken by Discovery Studio 2.5 software searching for the 3D-pharmacophoric modeling in which alignment of the synthesized conjugates describes the observed biological properties.^26^

##### Mycobacterium marinum

3D-pharmacophoric modeling of the biologically active compounds against *Mycobacterium marinum* reveals 3D-array of three chemical features [two hydrogen bonding acceptors (HBA-1, HBA-2) and one hydrogen bonding donor (HBD)] (ESI Fig. S1 and S2†). The estimated/predicted properties of the tested compounds due to alignment in the 3D-pharmacophore are presented in ESI Table S11.† From the observed data it has been noticed that, the N-2 of isonicotinic acid hydrazide is aligned with the pharmacophoric HBD in compounds 12d and 12g, which are effective conjugates synthesized against *M. marinum* (MIC_observed_ = 26.7, 46.6; MIC_estimated_ = 33.4, 38.1 μM for 12d and 12g, respectively). However, slight displacement is observed for compounds 12a, b and 12e, “which are also promising agents relative to the standard reference used (INH)” where, the N-1 of isonicotinic acid hydrazide of these compounds is aligned with the pharmacophoric HBD (MIC_observed_ = 56.1, 58.4, 51.2; MIC_estimated_ = 46.9, 43.7, 39.3 μM for 12a, b and 12e, respectively). The estimated properties of the tested compounds are validated with the experimental observations which help to establish the SAR.

##### Mycobacterium fortuitum

Three chemical features [two hydrogen bonding donors (HBD-1, HBD-2) and one hydrogen bonding acceptor (HBA)] were exhibited by the 3D-pharmacophore due to the tested bioactive agents against *Mycobacterium fortuitum* (ESI Fig. S3 and S4†). Compound 12g, which is one of the high potent hits synthesized against *M. fortuitum* reveals alignment of isonicotinic acid hydrazide N-1 with HBD-1 (MIC_observed_ = 46.6, MIC_estimated_ = 44.7 μM). However, N-1 and N-2 of the hyrazide moiety of compounds 12d and 12a are aligned with HBD-2 and HBD-1, respectively (MIC_observed_ = 53.4, 56.1, MIC_estimated_ = 50.6, 52.7 μM for 12d and 12a, respectively). Meanwhile, the nitrogen atom of pyrazinecarboxamide function of compounds 12e and 12b are aligned with HBD-1 and HBD-2, respectively (MIC_observed_ = 51.2, 58.4, MIC_estimated_ = 48.0, 56.9 μM for 12e and 12b, respectively) (ESI Table S11†).

##### Mycobacterium tuberculosis

Three chemical features (hydrogen bonding donor, acceptor and a hydrophobic) were observed for the biologically active compounds against *M. tuberculosis* in the 3D-pharmacophoric study utilizing their % growth inhibition at 30 μg mL^−1^. All the tested conjugates (12a–c, 12e, and 12g) show alignment of the isonicotinyl N-1 and amino acid carbonyl with the hydrogen bonding donor and acceptor, respectively while the pyridinyl heterocycle is aligned with the hydrophobic function. Compound 13 also reveals a similar alignment with a slight difference where the carbonyl of pyrazinoic acid is aligned with the hydrogen bonding donor. Compounds 10a, c, d, e show alignment of the amino acid carbonyl and its alkyl fragment with the hydrogen bonding acceptor and hydrophobic, respectively while the pyrazinamide nitrogen with the hydrogen bonding donor function. Slight division was observed for compounds 10b, g where the pyrazinyl heterocycle N-1 is aligned with the hydrogen bonding acceptor function. Most of the estimated properties due to the pharmacophoric model preserve the potencies of the tested compounds (ESI Table S12, Fig. S5 and S6†).

From all the above 3D-pharmocophoric model observations it is obvious that, alignment of various nitrogen atoms with the pharmacophoric functions, is the main controlling parameter revealing bio-properties. These are common observations between the two techniques utilized in the present computational study (2D-QSAR and 3D-pharmacophore) where, the 2D-QSAR studies reveal important descriptors dealing with nitrogen atoms governing bio-properties (max. e–n attraction for bond C–N for the QSAR model of *M. marinum*, max. e–e repulsion for atom N, avg. electroph. react. index for atom N for the QSAR model of *M. fortuitum* and min. e–e repulsion for bond H–N for the QSAR model of *M. tuberculosis*).

### ADMET (absorption, distribution, metabolism, excretion and toxicity) studies

Computed ADMET studies were undertaken by Discovery Studio 2.5 software. From the results obtained it has been noticed that most of the tested compounds show optimal aqueous solubility and good to moderate intestinal absorption. Blood brain barrier penetration (BBB) is ranging from low to very low level. Many of the tested compounds show non-hepatotoxicity (Table 3). These computational observations indicate that many of the constructed compounds are good hits to be handled by more sophisticated biological/pharmacological studies for optimizing promising bio-active agents. The predicted ADMET properties are encouraging and we will further study the best conjugates on animal models to generate experimental data. In addition, our synthesized conjugates follow the Lipinski rule of five, which is used to evaluate drug likeness.

**Table 3:** ADMET descriptor values for the tested compounds

Entry	Compd	A.S.^a^	I.A.^b^	BBB^c^	PPB^d^
1	PZA	5	1	3	0
2	INH	4	0	3	1
3	10a	4	0	3	0
4	10b	4	0	3	0
5	10c	4	0	3	0
6	10d	4	0	3	0
7	10e	4	0	3	2
8	10g	3	0	3	2
9	12a	4	1	4	0
10	12b	4	1	4	0
11	12c	4	1	4	0
12	12d	4	1	4	1
13	12e	4	1	4	2
14	12g	3	1	4	2
15	13	4	0	4	2
16	16	4	0	3	2

a A.S. (aqueous solubility) level: 0, 1, 2, 3, 4, 5 and 6 corresponding to extremely low, very low, low; good, optimal, too soluble and unknown, respectively.

b I.A. (intestinal absorption) level: 0, 1, 2 and 3 corresponding to good, moderate, poor and very poor, respectively.

c BBB (blood brain barrier penetration) level: 0, 1,2,3, 4 corresponding to very good, high, medium, low and very low, respectively.

d PPB (plasma protein binding) level: 0, 1, 2 corresponding to <90%, >90% and >95%, respectively.

## Conclusions

In conclusion, a series of novel hybrid conjugates of pyrazinoic acid, isoniazid was synthesized using optimized benzotriazole, and microwave assisted methodology. The synthesized conjugates showed strikingly high activity against various bacterial and mycobacterial strains relative to the parent drugs. The presence of amino acid in the hybrid conjugates also participates in enhancing the activity. The biological data was supported by different computational techniques (3D-pharmacophore and 2D-QSAR). The robust models could be utilized further for developing more potent drug-like molecules. In our future studies, we will explore the possibilities of other linkers as well as dipeptides and tripeptides to unfold the structural activity relationship. In our future study, we will further investigate the molecular mechanism of action by using animal models.

## Experimental section

### Chemistry

#### General methods

Melting points were determined on a capillary point apparatus equipped with a digital thermometer. Reactions were monitored using thin layer chromatography (TLC) on 0.2 mm silica gel F254 plates (Merck). The chemical structures of final products and intermediates were characterized by nuclear magnetic resonance spectra (^1^H NMR, ^13^C NMR) and determined on a Bruker NMR spectrometer (500 MHz, 125 MHz). ^13^C NMR spectra are fully decoupled. Chemical shifts were reported in parts per million (ppm) using a deuterated solvent peak or tetramethylsilane as an internal standard. The chemical structures of final products were confirmed on a high-resolution Biosystems QStar Elite time-of-flight electrospray mass spectrometer. HRMS was performed on an Agilent 6210 instrument using time-of-flight mass spectrometry (TOF-MS) with electrospray ionization (ESI). HPLC studies carried out on Agilent 6120 LCMS instrument using Chirobiotic T column.

#### General procedure: synthesis of pyrazinoic acid–amino acid conjugates 10a–g

A 50 mL round bottom flask containing a small stir bar was charged with benzotriazole activated pyrazinoic acid 8 (0.5 g, 2.22 mM) and amino acid 9a–g (2.22 mM) dissolved in a mixture acetonitrile and water (7 : 3) along with trimethylamine (3.33 mM). The reaction mixture was stirred at room temperature for 3–4 h and TLC monitored the progress of the reaction. After completion of the reaction, the acetonitrile was evaporated under reduced pressure and the residue was treated with 4 N HCl solution. The precipitate formed was filtered, washed with water and dried under vacuum to get the desired products 10a–g.

##### (Pyrazine-2-carbonyl)-l-leucine (10a)

White amorphous, yield (78%), mp 155–156 °C; ^1^H NMR (500 MHz, CDCl_3_) *δ* 9.40 (s, 1H), 8.78 (s, 1H), 8.58 (s, 1H), 8.12 (d, *J* = 8.4 Hz, 1H), 4.89–4.54 (m, 1H), 1.91–1.76 (m, 3H), 1.01 (d, *J* = 4.8 Hz, 6H). ^13^C NMR (125 MHz, CDCl_3_) *δ* 176.7, 163.0, 147.3, 144.3, 144.1, 142.9, 50.8, 41.3, 25.0, 22.9, 21.8. HRMS *m*/*z* for C_11_H_15_N_3_O_3_ [M + H]^+^ calcd 238.1186. Found: 238.1189.

##### (Pyrazine-2-carbonyl)-l-valine (10b)

White amorphous, yield (88%), mp 160–162 °C; ^1^H NMR (500 MHz, CDCl_3_) *δ* 9.40 (s, 1H), 8.78 (s, 1H), 8.59 (s, 1H), 8.26 (d, *J* = 8.8 Hz, 1H), 4.80 (dd, *J* = 8.8, 4.5 Hz, 1H), 2.45–2.37 (m, 1H), 1.07 (t, *J* = 6.0 Hz, 6H). ^13^C NMR (125 MHz, CDCl_3_) *δ* 175.7, 163.1, 147.3, 147.2, 144.3, 144.2, 144.2, 142.9, 57.2, 31.3, 19.2, 17.7. HRMS *m*/*z* for C_10_H_13_N_3_O_3_ [M + H]^+^ calcd 224.1029. Found: 224.1031.

##### (Pyrazine-2-carbonyl)-l-alloisoleucine (10c)

White amorphous, yield (81%), mp 164–166 °C; ^1^H NMR (500 MHz, CDCl_3_) *δ* 9.40 (s, 1H), 8.78 (s, 1H), 8.59 (s, 1H), 8.27 (d, *J* = 8.7 Hz, 1H), 4.84 (dd, *J* = 8.8, 4.6 Hz, 1H), 2.14–2.08 (m, 1H), 1.66–1.55 (m, 1H), 1.38–1.27 (m, 1H), 1.05 (d, *J* = 6.8 Hz, 3H), 0.99 (t, *J* = 7.4 Hz, 3H). ^13^C NMR (125 MHz, CDCl_3_) *δ* 175.8, 162.9, 147.3, 144.3, 142.9, 56.6, 37.9, 25.1, 15.7, 11.6. HRMS *m*/*z* for C_11_H_15_N_3_O_3_ [M + H]^+^ calcd 238.1186. Found: 238.1182.

##### (Pyrazine-2-carbonyl)-l-methionine (10d)

White amorphous, yield (76%), mp 136–138 °C; ^1^H NMR (500 MHz, CDCl_3_) *δ* 10.27 (bs, 1H), 9.40 (s, 1H), 8.80 (s, 1H), 8.60 (s, 1H), 8.40 (d, *J* = 8.0 Hz, 1H), 4.99 (dd, *J* = 12.6, 7.5 Hz, 1H), 2.64 (t, *J* = 7.3 Hz, 2H), 2.43–2.33 (m, 1H), 2.27–2.16 (m, 1H), 2.12 (s, 3H). ^13^C NMR (125 MHz, CDCl_3_) *δ* 175.4, 163.1, 147.3, 144.2, 143.0, 51.6, 31.5, 30.1, 15.5. HRMS *m*/*z* for C_10_H_13_N_3_O_3_S [M + H]^+^ calcd 256.0750. Found: 256.0755.

##### (Pyrazine-2-carbonyl)-l-phenylalanine (10e)

Yellow amorphous, yield 0.15 g (98%), mp 165–166 °C; ^1^H NMR (500 MHz, DMSO-*d*_6_) *δ* 13.01 (bs, 1H), 9.17 (s, 1H), 8.88 (s, 1H), 8.87 (s, 1H), 8.74 (s, 1H), 7.27 (s, 4H), 7.21–7.16 (m, 1H), 4.99 (dd, *J* = 12.6, 7.8 Hz, 1H), 3.30–3.20 (m, 2H). ^13^C NMR (125 MHz, DMSO-*d*_6_) *δ* 172.4, 162.6, 147.9, 144.1, 143.5, 137.5, 129.1, 128.3, 126.5, 53.5, 36.1. HRMS *m*/*z* for C_14_H_13_N_3_O_3_ [M + H]^+^ calcd 272.1029. Found: 272.1032.

##### (Pyrazine-2-carbonyl)-dl-phenylalanine (10f)

Yellow amorphous, yield (94%), mp 155–157 °C; ^1^H NMR (500 MHz, DMSO-*d*_6_) *δ* 9.15 (s, 1H), 8.88 (s, 1H), 8.86 (s, 1H), 8.74 (s, 1H), 7.25 (s, 4H), 7.23–7.14 (m, 1H), 4.81–4.73 (m, 1H), 3.29–3.18 (m, 2H). ^13^C NMR (125 MHz, DMSO-*d*_6_) *δ* 172.4, 162.7, 147.9, 144.1, 143.5, 137.5, 129.1, 128.3, 126.5, 53.5, 36.1. HRMS *m*/*z* for C_14_H_13_N_3_O_3_ [M + H]^+^ calcd 272.1029. Found: 272.1030.

##### (Pyrazine-2-carbonyl)-l-tryptophan (10g)

Yellow amorphous, yield (93%), mp 127–129 °C; ^1^H NMR (500 MHz, DMSO-*d*_6_) *δ* 13.01 (bs, 1H), 10.85 (s, 1H), 9.17 (s, 1H), 8.88 (s, 1H), 8.72 (d, *J* = 7.7 Hz, 1H), 8.70 (s, 1H), 7.52 (d, *J* = 8.0 Hz, 1H), 7.32 (d, *J* = 8.0 Hz, 1H), 7.16 (s, 1H), 7.04 (t, *J* = 7.4 Hz, 1H), 6.92 (*t*, *J* = 7.1 Hz, 1H), 4.82–4.75 (m, 1H), 3.36 (s, 2H). ^13^C NMR (125 MHz, DMSO-*d*_6_) *δ* 172.6, 162.4, 147.7, 143.9, 143.3, 136.0, 127.1, 123.6, 120.9, 118.1, 111.3, 109.4, 52.8, 26.5. HRMS *m*/*z* for C_16_H_14_N_4_O_3_ [M + H]^+^ calcd 311.1138. Found: 311.1145.

#### General procedure: synthesis of pyrazinoic acid–aminoacyl benzotriazolides 11a–g

1*H*-Benzotriazole 7 (4.0 mM) was dissolved in anhydrous methylene chloride (30 mL). Thionyl chloride (1.2 mM) was added and stirred for 30 min. Reduced the reaction mixture temperature to −15 °C and then corresponding pyrazinoic acid–amino acid conjugates (10a–g, 1.0 mM) was added. The reaction mixture was stirred for 4–5 h at −15 °C. Upon completion of the reaction, the reaction mixture was filtered and the filtrate was evaporated under reduced pressure. The residue was treated with 10% solution of sodium carbonate and the precipitate obtained was filtered, washed with water and dried to yield the desired product 11a–g.

##### (*S*)-*N*-(1-(1*H*-Benzo[*d*][1,2,3]triazol-1-yl)-4-methyl-1-oxopentan-2-yl)pyrazine-2-carboxamide (11a)

White amorphous, yield (88%), mp 144–146 °C; ^1^H NMR (500 MHz, CDCl_3_) *δ* 9.37 (s, 1H), 8.78 (s, 1H), 8.59 (s, 1H), 8.46 (d, *J* = 8.2 Hz, 1H), 8.23 (d, *J* = 8.2 Hz, 1H), 8.12 (d, *J* = 8.2 Hz, 1H), 7.64 (t, *J* = 7.8 Hz, 1H), 7.51 (t, *J* = 7.8 Hz, 1H), 6.28–6.21 (m, 1H), 2.09–1.84 (m, 3H), 1.12 (d, *J* = 6.6 Hz, 3H), 0.99 (d, *J* = 6.6 Hz, 3H). ^13^C NMR (125 MHz, CDCl_3_) *δ* 171.6, 163.2, 147.7, 146.1, 144.5, 143.9, 142.8, 131.2, 130.8, 126.5, 120.4, 114.4, 51.8, 41.8, 25.5, 23.2, 21.4. HRMS *m*/*z* for C_17_H_18_N_6_O_2_ [M + H]^+^ calcd 339.1564. Found: 339.1570.

##### (*S*)-*N*-(1-(1*H*-Benzo[*d*][1,2,3]triazol-1-yl)-3-methyl-1-oxobutan-2-yl)pyrazine-2-carboxamide (11b)

White amorphous, yield (77%), mp 133–135 °C; ^1^H NMR (500 MHz, CDCl_3_) *δ* 9.41 (s, 1H), 8.80 (s, 1H), 8.63 (s, 1H), 8.57 (d, *J* = 8.2 Hz, 1H), 8.29 (d, *J* = 8.2 Hz, 1H), 8.16 (d, *J* = 8.2 Hz, 1H), 7.69 (t, *J* = 7.5 Hz, 1H), 7.55 (t, *J* = 7.5 Hz, 1H), 6.18 (dd, *J* = 8.7, 5.2 Hz, 1H), 2.74–2.64 (m, 1H), 1.19 (d, *J* = 6.8 Hz, 3H), 1.10 (d, *J* = 6.8 Hz, 3H). ^13^C NMR (125 MHz, CDCl_3_) *δ* 170.9, 163.3, 147.7, 146.1, 144.6, 144.0, 142.8, 131.1, 130.8, 126.6, 120.5, 114.4, 57.7, 31.9, 19.9, 17.3. HRMS *m*/*z* for C_16_H_16_N_6_O_2_ [M + H]^+^ calcd 325.1407. Found: 325.1411.

##### *N*-((2*S*,3*S*)-1-(1*H*-Benzo[*d*][1,2,3]triazol-1-yl)-3-methyl-1-oxopentan-2-yl)pyrazine-2-carboxamide (11c)

White amorphous, yield (69%), mp 186–188 °C; ^1^H NMR (500 MHz, CDCl_3_) *δ* 9.40 (s, 1H), 8.80 (s, 1H), 8.62 (s, 1H), 8.56 (d, *J* = 8.5 Hz, 1H), 8.29 (d, *J* = 8.2 Hz, 1H), 8.16 (d, *J* = 8.2 Hz, 1H), 7.69 (t, *J* = 7.5 Hz, 1H), 7.55 (t, *J* = 7.5 Hz, 1H), 6.18 (dd, *J* = 8.7, 5.2 Hz, 1H), 2.50–2.38 (m, 1H), 1.74–1.64 (m, 1H), 1.43–1.31 (m, 1H), 1.15 (d, *J* = 6.8 Hz, 3H), 0.95 (t, *J* = 7.4 Hz, 3H). ^13^C NMR (125 MHz, CDCl_3_) *δ* 170.9, 163.2, 147.7, 146.1, 144.6, 143.9, 142.8, 131.1, 130.8, 126.6, 120.5, 114.4, 57.4, 38.4, 24.4, 16.2, 11.3. HRMS *m*/*z* for C_17_H_18_N_6_O_2_ [M + H]^+^ calcd 339.1564. Found: 339.1573.

##### (*S*)-*N*-(1-(1*H*-Benzo[*d*][1,2,3]triazol-1-yl)-4-(methylthio)-1-oxobutan-2-yl)pyrazine-2-carboxamide (11d)

White amorphous, yield (74%), mp 171–173 °C; ^1^H NMR (500 MHz, CDCl_3_) *δ* 9.42 (s, 1H), 8.82 (s, 1H), 8.69 (d, *J* = 7.9 Hz, 1H), 8.64 (s, 1H), 8.29 (d, *J* = 8.2 Hz, 1H), 8.18 (d, *J* = 8.2 Hz, 1H), 7.71 (t, *J* = 7.5 Hz, 1H), 7.57 (t, *J* = 7.5 Hz, 1H), 6.34 (dd, *J* = 16.5, 45.2 Hz, 1H), 2.77 (t, *J* = 7.2 Hz, 2H), 2.69–2.59 (m, 1H), 2.45–2.34 (m, 1H), 2.12 (s, 3H). ^13^C NMR (125 MHz, CDCl_3_) *δ* 170.6, 163.2, 147.8, 146.1, 142.8, 131.2, 131.0, 126.7, 120.5, 114.4, 52.7, 32.4, 30.3, 15.5. HRMS *m*/*z* for C_16_H_16_N_6_O_2_S [M + H]^+^ calcd 357.1128. Found: 357.1125.

##### (*S*)-*N*-(1-(1*H*-Benzo[*d*][1,2,3]triazol-1-yl)-1-oxo-3-phenylpropan-2-yl)pyrazine-2-carboxamide (11e)

Light yellow amorphous, yield (90%), mp 180–182 °C; ^1^H NMR (500 MHz, DMSO-*d*_6_) *δ* 9.65 (d, *J* = 6.4 Hz, 1H), 9.11 (s, 1H), 8.91 (s, 1H), 8.80 (s, 1H), 8.29 (d, *J* = 8.2 Hz, 1H), 8.22 (d, *J* = 8.2 Hz, 1H), 7.82 (t, *J* = 7.4 Hz, 1H), 7.65 (t, *J* = 7.4 Hz, 1H), 7.36 (d, *J* = 7.0 Hz, 2H), 7.25 (t, *J* = 6.8 Hz, 2H), 7.21–7.14 (m, 1H), 6.20–6.12 (m, 1H), 3.49 (d, *J* = 6.3 Hz, 2H). ^13^C NMR (125 MHz, DMSO-*d*_6_) *δ* 170.5, 163.6, 148.1, 145.4, 143.9, 143.6, 136.8, 131.2, 130.6, 129.1, 128.4, 126.8, 120.3, 113.9, 54.7, 36.1. HRMS *m*/*z* for C_20_H_16_N_6_O_2_ [M + H]^+^ calcd 373.1408. Found: 373.1412.

##### *N*-(1-(1*H*-Benzo[*d*][1,2,3]triazol-1-yl)-1-oxo-3-phenylpropan-2-yl)pyrazine-2-carboxamide (11f)

Light yellow amorphous, yield (87%), mp 172–174 °C; ^1^H NMR (500 MHz, DMSO-*d*_6_) *δ* 9.66 (d, *J* = 6.5 Hz, 1H), 9.12 (s, 1H), 8.92 (s, 1H), 8.81 (s, 1H), 8.29 (d, *J* = 8.2 Hz, 1H), 8.23 (d, *J* = 8.2 Hz, 1H), 7.82 (t, *J* = 7.4 Hz, 1H), 7.65 (t, *J* = 7.4 Hz, 1H), 7.36 (d, *J* = 7.0 Hz, 2H), 7.25 (t, *J* = 6.8 Hz, 2H), 7.21–7.14 (m, 1H), 6.21–6.13 (m, 1H), 3.50 (d, *J* = 6.4 Hz, 2H). ^13^C NMR (125 MHz, DMSO-*d*_6_) *δ* 170.5, 163.6, 148.1, 145.4, 143.9, 143.6, 136.8, 131.2, 130.6, 129.1, 128.4, 126.8, 120.3, 113.9, 54.6, 36.0. HRMS *m*/*z* for C_20_H_16_N_6_O_2_ [M + H]^+^ calcd 373.1408. Found: 373.1410.

##### (*S*)-*N*-(1-(1*H*-Benzo[*d*][1,2,3]triazol-1-yl)-3-(1*H*-indol-3-yl)-1-oxopropan-2-yl)pyrazine-2-carboxamide (11g)

Light yellow amorphous, yield (86%), mp 169–171 °C; ^1^H NMR (500 MHz, DMSO-*d*_6_) *δ* 10.87 (s, 1H), 9.47 (d, *J* = 6.8 Hz, 1H), 9.12 (s, 1H), 8.91 (s, 1H), 8.79 (s, 1H), 8.29 (d, *J* = 8.2 Hz, 1H), 8.21 (d, *J* = 8.2 Hz, 1H), 7.82 (t, *J* = 7.4 Hz, 1H), 7.69–7.61 (m, 2H), 7.30 (s, 1H), 7.05 (t, *J* = 7.4 Hz, 1H), 6.94 (d, *J* = 7.4 Hz, 1H), 7.16 (s, 1H), 7.04 (t, *J* = 7.4 Hz, 1H), 6.92 (*t*, *J* = 7.1 Hz, 1H), 6.20 (dd, *J* = 13.3, 6.4 Hz, 1H), 5.76 (s, 1H), 3.64–3.58 (m, 2H). ^13^C NMR (125 MHz, DMSO-*d*_6_) *δ* 172.8, 162.4, 147.7, 143.8, 143.3, 143.6, 136.0, 131.1, 130.5, 127.1, 123.5, 121.0, 120.3, 118.1, 113.7, 111.3, 109.4, 52.8, 26.5. HRMS *m*/*z* for C_22_H_17_N_7_O_2_ [M + H]^+^ calcd 412.1516. Found: 412.1524.

#### General procedure for the synthesis of pyrazinoic acid–isoniazid hybrid conjugates 12a–g

A dried heavy-walled Pyrex tube containing a small stir bar was charged with benzotriazole intermediate 11a–g (0.7 mM) and isoniazid 4 (0.7 mM) dissolved in THF (3 mL) along with trimethylamine (1.1 mM). The reaction mixture was exposed to microwave irradiation (50 W) at a temperature of 70 °C for 1 h. Each mixture was allowed to cool through an inbuilt system until the temperature had fallen below 30 °C (*ca.* 10 min). The reaction mixture was quenched with ice cold water and the solid obtained was filtered and washed with 10% Na_2_CO_3_ and water to give the desired compounds 12a–g.

##### (*S*)-*N*-(1-(2-Isonicotinoylhydrazineyl)-4-methyl-1-oxopentan-2-yl)pyrazine-2-carboxamide (12a)

White amorphous, yield (85%), mp 160–162 °C; ^1^H NMR (500 MHz, DMSO-*d*_6_) *δ* 10.74 (s, 1H), 10.39 (s, 1H), 9.22 (s, 1H), 8.91 (s, 1H), 8.82–8.67 (m, 3H), 8.70 (d, *J* = 8.7 Hz, 1H), 7.78 (d, *J* = 4.3 Hz, 2H), 4.80–4.72 (m, 1H), 1.86–1.64 (m, 3H), 0.96 (d, *J* = 4.7 Hz, 3H), 0.94 (d, *J* = 4.7 Hz, 3H). ^13^C NMR (125 MHz, DMSO-*d*_6_) *δ* 170.9, 163.9, 162.6, 150.4, 147.8, 144.2, 143.5, 139.3, 121.3, 50.0, 41.2, 24.3, 22.9, 21.6. HRMS *m*/*z* for C_17_H_20_N_6_O_3_ [M + H]^+^ calcd 357.1670. Found: 357.1678.

##### (*S*)-*N*-(1-(2-Isonicotinoylhydrazineyl)-3-methyl-1-oxobutan-2-yl)pyrazine-2-carboxamide (12b)

White amorphous, yield (87%), mp 175–177 °C; ^1^H NMR (500 MHz, DMSO-*d*_6_) *δ* 10.74 (s, 1H), 10.44 (s, 1H), 9.23 (s, 1H), 8.92 (s, 1H), 8.82–8.72 (m, 3H), 8.50 (d, *J* = 10.6 Hz, 1H), 7.77 (d, *J* = 4.6 Hz, 2H), 4.59–4.52 (m, 1H), 2.25–2.15 (m, 1H), 1.04 (d, *J* = 6.6 Hz, 3H), 0.98 (d, *J* = 6.6 Hz, 3H). ^13^C NMR (125 MHz, DMSO-*d*_6_) *δ* 170.1, 164.39, 162.8, 150.9, 148.5, 144.4, 144.0, 139.7, 121.8, 57.0, 40.5, 40.4, 40.2, 40.0, 39.8, 39.7, 39.5, 31.7, 19.6, 18.7. HRMS *m*/*z* for C_16_H_18_N_6_O_3_ [M + H]^+^ calcd 343.1513. Found: 343.1524.

##### *N*-((2*S*,3*S*)-1-(2-Isonicotinoylhydrazineyl)-3-methyl-1-oxopentan-2-yl)pyrazine-2-carboxamide (12c)

White amorphous, yield (80%), mp 168–170 °C; ^1^H NMR (500 MHz, DMSO-*d*_6_) *δ* 10.75 (s, 1H), 10.46 (s, 1H), 9.23 (s, 1H), 8.92 (s, 1H), 8.82–8.72 (m, 3H), 8.52 (d, *J* = 9.2 Hz, 1H), 7.78 (d, *J* = 4.0 Hz, 2H), 4.63–4.53 (m, 1H), 2.09–1.92 (m, 1H), 1.66–1.50 (m, 1H), 1.30–1.12 (m, 1H), 1.10–0.85 (m, 6H). ^13^C NMR (125 MHz, DMSO-*d*_6_) *δ* 170.2, 164.4, 162.8, 150.9, 148.5, 144.4, 144.0, 139.8, 121.8, 56.1, 55.1, 38.2, 37.7, 26.2, 25.0, 15.7, 14.9, 12.0, 11.4. HRMS *m*/*z* for C_17_H_20_N_6_O_3_ [M + H]^+^ calcd 357.1670. Found: 357.1680.

##### (*S*)-*N*-(1-(2-Isonicotinoylhydrazineyl)-4-(methylthio)-1-oxobutan-2-yl)pyrazine-2-carboxamide (12d)

White amorphous, yield (78%), mp 145–147 °C; ^1^H NMR (500 MHz, DMSO-*d*_6_) *δ* 10.75 (s, 1H), 10.40 (s, 1H), 9.20 (s, 1H), 8.89 (s, 1H), 8.87 (d, *J* = 8.3 Hz, 1H), 8.80–8.71 (m, 3H), 7.77 (d, *J* = 4.7 Hz, 2H), 4.77 (dd, *J* = 14.5, 7.1 Hz, 1H), 2.66–2.53 (m, 2H), 2.19–2.11 (m, 2H), 2.07 (s, 3H). ^13^C NMR (125 MHz, DMSO-*d*_6_) *δ* 170.1, 164.0, 162.9, 150.4, 147.8, 144.3, 143.4, 139.3, 121.3, 51.0, 31.8, 29.4, 14.6. HRMS *m*/*z* for C_16_H_18_N_6_O_3_S [M + H]^+^ calcd 375.1234. Found: 375.1233.

##### (*S*)-*N*-(1-(2-Isonicotinoylhydrazineyl)-1-oxo-3-phenylpropan-2-yl)pyrazine-2-carboxamide (12e)

Light yellow amorphous, yield (95%), mp 177–179 °C; ^1^H NMR (500 MHz, DMSO-*d*_6_) *δ* 10.81 (s, 1H), 10.51 (s, 1H), 9.12 (s, 1H), 8.87 (s, 1H), 8.83–8.69 (m, 4H), 7.79 (s, 2H), 7.38–7.14 (m, 5H), 5.01–4.98 (m, 1H), 3.29–3.15 (m, 2H). ^13^C NMR (125 MHz, DMSO-*d*_6_) *δ* 170.0, 163.9, 162.5, 150.4, 147.8, 144.1, 143.4, 139.3, 137.3, 129.3, 128.2, 126.5, 121.3, 52.8, 37.5. HRMS *m*/*z* for C_20_H_18_N_6_O_3_ [M + H]^+^ calcd 391.1513. Found: 391.1510.

##### *N*-(1-(2-Isonicotinoylhydrazineyl)-1-oxo-3-phenylpropan-2-yl)pyrazine-2-carboxamide (12f)

Light yellow amorphous, yield (94%), mp 143–145 °C; ^1^H NMR (500 MHz, DMSO-*d*_6_) *δ* 10.81 (s, 1H), 10.52 (s, 1H), 9.12 (s, 1H), 8.88 (s, 1H), 8.84–8.69 (m, 4H), 7.79 (s, 2H), 7.38–7.11 (m, 5H), 4.99–4.89 (m, 1H), 3.29–3.15 (m, 2H). ^13^C NMR (125 MHz, DMSO-*d*_6_) *δ* 170.1, 164.0, 162.6, 150.5, 147.8, 144.1, 143.5, 139.3, 137.4, 129.3, 128.2, 126.5, 121.4, 52.9, 37.5. HRMS *m*/*z* for C_20_H_18_N_6_O_3_ [M + H]^+^ calcd 391.1513. Found: 391.1508.

##### (*S*)-*N*-(3-(1*H*-Indol-3-yl)-1-(2-isonicotinoylhydrazineyl)-1-oxopropan-2-yl)pyrazine-2-carboxamide (12g)

Light yellow amorphous, yield (78%), mp 243–245 °C; ^1^H NMR (500 MHz, DMSO-*d*_6_) *δ* δ 10.80 (s, 1H), 10.52 (s, 1H), 9.25 (s, 1H), 8.84 (s, 1H), 8.70–8.59 (m, 3H), 7.74–7.60 (m, 6H), 7.35–7.08 (m, 4H), 4.64–4.59 (m, 1H), 3.24–3.17 (m, 2H). ^13^C NMR (125 MHz, DMSO-*d*_6_) *δ* 170.6, 165.4, 162.8, 150.1, 148.1, 146.0, 144.0, 144.0, 136.7, 134.0, 127.4, 123.4, 121.9, 120.0, 119.8, 118.6, 112.3, 110.0, 55.8, 27.8. HRMS *m*/*z* for C_22_H_19_N_7_O_3_ [M + H]^+^ calcd 430.1622. Found: 430.1638.

#### *N*′-Isonicotinoylpyrazine-2-carbohydrazide (13)

A dried heavy-walled Pyrex tube containing a small stir bar was charged with benzotriazole activated pyrazinoic acid 8 (0.7 mM) and isoniazid 4 (0.7 mM) dissolved in THF (3 mL) along with trimethylamine (1.1 mM). The reaction mixture was exposed to microwave irradiation (50 W) at a temperature of 70 °C for 1 h. The mixture was allowed to cool through an inbuilt system until the temperature had fallen below 30 °C (*ca.* 10 min). Upon completion the reaction mixture was quenched with ice cold water and the solid obtained was filtered and washed with 10% Na_2_CO_3_ and water to give the desired compound. White amorphous, yield (88%), mp 208–210 °C;^17^^1^H NMR (500 MHz, DMSO-*d*_6_) *δ* 10.96 (bs, 1H), 10.91 (s, 1H), 9.22 (s, 1H), 8.94 (s, 1H), 8.82–8.77 (m, 3H), 7.82 (d, *J* = 4.7 Hz, 2H). ^13^C NMR (125 MHz, DMSO-*d*_6_) *δ* 164.0, 162.3, 150.5, 148.1, 144.1, 143.8, 143.7, 139.4, 121.3. HRMS *m*/*z* for C_11_H_9_N_5_O_2_ [M + H]^+^ calcd 244.0829. Found: 244.0831.

#### *N′-*Isonicotinoylisonicotinohydrazide (16)

A dried heavy-walled Pyrex tube containing a small stir bar was charged with benzotriazole activated isonicitinic acid 15 (0.7 mM) and isoniazid 4 (0.7 mM) dissolved in THF (3 mL) along with trimethylamine (1.1 mM). The reaction mixture was exposed to microwave irradiation (50 W) at a temperature of 70 °C for 1 h. The mixture was allowed to cool through an inbuilt system until the temperature had fallen below 30 °C (*ca.* 10 min). Upon completion, the reaction mixture was quenched with ice-cold water and the solid obtained was filtered, washed with 10% Na_2_CO_3_ and water to give the desired compound 16. White amorphous, yield (91%), mp 212–214 °C;^18–20^^1^H NMR (500 MHz, DMSO-*d*_6_) *δ* 10.95 (bs, 2H), 8.80 (d, *J* = 4.6 Hz, 2H), 7.82 (d, *J* = 4.6 Hz, 2H). ^13^C NMR (125 MHz, DMSO-*d*_6_) *δ* 164.3, 150.5, 139.3, 121.3. HRMS *m*/*z* for C_12_H_10_N_4_O_2_ [M + H]^+^ calcd 243.0877. Found: 243.0886.

### Biological studies

#### Aerobic antibacterial studies

Antibacterial properties were investigated for the synthesized compounds against a variety of Gram-positive (*Staphylococcus aureus*, *Enterococcus faecalis*) and Gram-negative (*Klebsiella pneumonia*, *Proteus vulgaris*, *Pseudomonas aeruginosa*) clinical isolate bacteria utilizing the standard technique.^21^

#### Antimycobacterial studies

Antimycobacterial properties of the synthesized compounds and standards were screened against clinical isolate tuberculous (*Mycobacterium bovis*) and non-tuberculous (*Mycobacterium marinum*, *Mycobacterium fortuitum*) strains using the standard technique^21,22^ and against *Mycobacterium tuberculosis* by the following described method. Highly virulent *M. tuberculosis* Erdman strain expressing luciferase (Mtb-Lux Erdman) was grown in 7H9 Middlebrook media supplemented with oleic acid, dextrose, albumin, catalase (OADC) and 0.05% tween 80 at 37 °C. PH of the culture media was maintained at neutral pH (pH = 7.0). All the antibiotics were dissolved in 100% DMSO at a final concentration of 10 mg mL^−1^ 10^6^ bacteria per mL were incubated with increasing concentration of antibiotics for 7 days in a round bottom 96 well plate without shaking in a final volume of 300 μl. Bacterial growth was determined by measuring the luciferase expression (expressed in counts) in a PerkinElmer IVIS-spectrum imaging system kept at the BSL-3 facility of Albany Medical College, Albany, NY. Antibiotics concentrations manifesting >85% growth inhibition as measured by a decline in luciferase counts was considered as effective concentration for antimycobacterial activity.

#### Antiproliferative properties

Antiproliferative properties of the synthesized compounds were tested against RPE1 (normal human immortalized retinal epithelial) cell line at 100 μM to determine the toxicity/selectivity towards normal cell by the standard MTT technique.^23^

### Computational studies

#### 2D-QSAR modelling

2D-QSAR modeling was conducted by the standard technique utilizing CODESSA-Pro software.^24^

#### 3D-pharmacophore modeling

3D-pharmacophore modeling was undertaken by the standard technique utilizing Discovery Studio 2.5 software.^26^

### ADMET studies

Computed ADMET studies were conducted by the standard technique utilizing Discovery Studio 2.5 software.^23^

## Conflicts of interest

The authors have declared no conflict of interest.

## Supplementary Material

RA-009-C9RA03380G-s001
