# Supplementary material for: Synthesis, computational studies, antimycobacterial and antibacterial properties of pyrazinoic acid–isoniazid hybrid conjugates
Source: RSC Adv. 2019 Jul 1;9(35):20450–62. doi: 10.1039/c9ra03380g (PMC9065575; doi:10.1039/c9ra03380g)

Electronic Supplementary Material (ESI) for RSC Advances.
This journal is © The Royal Society of Chemistry 2019

Supplementary material

Synthesis, computational studies, antimycobacterial and antibacterial properties of pyrazinoic acid-isoniazid hybrid conjugates

Siva S. Panda,*[a] Adel S. Girgis,*[b] Bibhuti B. Mishra,[c] Mohamed Elagawany,[ad] Venkatasai Devarapalli,[a] William F. Littlefield,[a] Ahmed Samir,[e] Walid Fayad,[f] Nehmedo G. Fawzy,[b] Aladdin M. Srour[g] and Riham M. Bokhtia[ah]

[a]*Department of Chemistry & Physics, Augusta University, Augusta, GA 30912, USA.*

[b]*Department of Pesticide Chemistry, National Research Centre, Dokki, Giza 12622, Egypt.*

[c]*Department of Immunology and Microbial Disease, Albany Medical College, 47 New Scotland Avenue, Albany, NY-12208, USA.*

[d]*Department of Pharmaceutical Chemistry, Faculty of Pharmacy, Damanhour University, Damanhour, Egypt.*

[e]*Microbiology Department, Faculty of Veterinary Medicine, Cairo University, Cairo, Egypt.*

[f]*Drug Bioassay-Cell Culture Laboratory, Pharmacognosy Department, National Research Centre, Dokki, Giza, 12622. Egypt.*

[g]*Department of Therapeutic Chemistry, National Research Centre, Dokki, Giza 12622, Egypt.*

[h]*Department of Pharmaceutical Organic Chemistry, Faculty of Pharmacy, Zagazig University, Zagazig, 44519, Egypt.*

* Corresponding authors. E-mail: sspanda12@gmail.com, girgisas10@yahoo.com

Table titles

Table S1. Anti-proliferative properties of the synthesized compounds against RPE1 cell line.

Table S2. Descriptor of the BMLR-QSAR model for the tested compounds against *Mycobacterium marinum*.

Table S3. Observed and estimated MIC values for the tested compounds against *Mycobacterium marinum* according to the BMLR-QSAR model.

Table S4. Molecular descriptor values of the BMLR-QSAR model for the tested compounds against *Mycobacterium marinum* according to the BMLR-QSAR model.

Table S5. Descriptor of the BMLR-QSAR model for the tested compounds against *Mycobacterium fortuitum*.

Table S6. Observed and estimated MIC values for the tested compounds against *Mycobacterium fortuitum* according to the BMLR-QSAR model.

Table S7. Molecular descriptor values of the BMLR-QSAR model for the tested compounds against *Mycobacterium fortuitum* according to the BMLR-QSAR model.

Table S8. Descriptor of the BMLR-QSAR model for the tested compounds against *Mycobacterium tuberculosis*.

Table S9. Observed and estimated % growth inhibition at 30 μg/mL values for the tested compounds against *Mycobacterium tuberculosis* according to the BMLR-QSAR model.

Table S10. Molecular descriptor values of the BMLR-QSAR model for the tested compounds against *Mycobacterium tuberculosis* according to the BMLR-QSAR model.

Table S11. Estimated/predicted activity values for the tested compounds against *M. marinum* and *M. fortuitum* mapped with the generated 3D-pharmacophore models.

Table S11. Estimated/predicted activity values for the tested compounds against *M. tuberculosis* mapped with the generated 3D-pharmacophore model.

Figure captions

Fig. S1. (A) Constraint distances "HBA-1 – HBA-2 = 4.800, HBA-1 – HBD = 3.026, HBA-2 – HBD = 4.909 Å" and (B) constraint angles "HBA-1 – HBA-2 – HBD = 36.29 °" of the generated 3D-pharmacophore for the synthesized bio-active compounds against

Mycobacterium marinum which contains two hydrogen bonding acceptors (HBA-1, HBA-2; green) and one hydrogen bonding donor (HBD; purple).

Fig. S2. 3D-pharmacophore mapped on the synthesized bio-active compounds against *Mycobacterium marinum*.

Fig. S3. (A) Constraint distances "HBD-1 – HBD-2 = 8.500, HBD-1 – HBA = 4.271, HBD-2 – HBA = 5.844 Å" and (B) constraint angles "HBD-1 – HBD-2 – HBA = 27.45 °" of the generated 3D-pharmacophore for the synthesized bio-active compounds against *Mycobacterium fortuitum* which contains two hydrogen bonding donors (HBD-1, HBD-2; purple) and one hydrogen bonding acceptor (HBA; green).

Fig. S4. 3D-pharmacophore mapped on the synthesized bio-active compounds against *Mycobacterium fortuitum*.

Fig. S5. (A) Constraint distances "HBA – HBD = 3.292, HBA – H = 6.327, HBD – H = 3.343 Å" and (B) constraint angles "HBA – HBD – H = 144.97 °" of the generated 3D-pharmacophore for the synthesized bio-active compounds against *Mycobacterium tuberculosis* which contains hydrogen bonding acceptor (HBA; green), hydrogen bonding donor (HBD; purple) and hydrophobic (H; light blue).

Fig. S6. 3D-pharmacophore mapped on the synthesized bio-active compounds against *Mycobacterium tuberculosis*.

^{1}H NMR, ^{13}C NMR and HRMS spectral charts of the synthesized compounds.

HPLC spectra of compounds **12e** and **12f**

Table S1. Anti-proliferative properties of the synthesized compounds against RPE1 cell line.

Entry	Compound	Percentage of cell proliferation ± SD
1	**10a**	84.6 ± 0.7
2	**10b**	82.1 ± 3.0
3	**10c**	81.8 ± 5.5
4	**10d**	82.9 ± 8.5
5	**10e**	90.4 ± 3.3
6	**10f**	86.5 ± 4.2
7	**10g**	80.1 ± 7.4
8	**12a**	80.1 ± 4.2
9	**12b**	96.4 ± 2.7
10	**12c**	84.6 ± 3.7
11	**12e**	83.2 ± 5.7
12	**12f**	95.5 ± 8.6
13	**12g**	92.2 ± 1.2
14	**13**	88.2 ± 5.1
15	**16**	83.5 ± 7.4

Table S2. Descriptor of the BMLR-QSAR model for the tested compounds against *Mycobacterium marinum*.

Entry	ID	Coefficient	*s*	*t*	Descriptor
1	0	74.8101	12.160	6.152	Intercept
2	D_1	-0.176724	0.037	-4.762	Max. e-n attraction for bond C-N
3	D_2	-0.113573	0.017	-6.809	Max. e-e repulsion for bond C-C

$N = 11$, $n = 2$, $R^2 = 0.908$, R^2cvOO = 0.829, $F = 39.543$, $s^2 = 0.003$

Log(MIC, μM) = 74.8101 – (0.176724 x D_1) – (0.113573 x D_2)

Table S3. Observed and estimated MIC values for the tested compounds against *Mycobacterium marinum* according to the BMLR-QSAR model.

Entry	Compd.	Observed MIC, mM	Estimated MIC, μM	Error[a]
1	**PZA**	81.2	86.8	-5.6
2	**INH**	72.9	80.5	-7.6
3	**10a**	84.3	79.8	4.5
4	**12a**	56.1	46.7	9.4
5	**12b**	58.4	57.4	1.0
6	**12c**	56.1	52.5	3.6
7	**12d**	26.7	30.8	-4.1
8	**12e**	51.2	46.3	4.9
9	**12g**	46.6	44.8	1.8
10	**13**	41.1	48.0	-6.9
11	**16**	82.6	82.3	0.3

[a] Error is the difference between the observed and estimated biologically activity (MIC) values.

Table S4. Molecular descriptor values of the BMLR-QSAR model for the tested compounds against *Mycobacterium marinum* according to the BMLR-QSAR model.

Entry	Compd.	Descriptors[a]	
		D_1	D_2
1	**PZA**	330.1631	127.8817
2	**INH**	329.9877	128.4424
3	**10a**	330.4742	127.7163
4	**12a**	330.664	129.4675
5	**12b**	330.6187	128.7496
6	**12c**	330.4774	129.3105
7	**12d**	330.7008	131.0057
8	**12e**	330.426	129.8765
9	**12g**	330.3036	130.193
10	**13**	330.8481	129.0796
11	**16**	329.2578	129.4907

[a] D_1 = Max. e-n attraction for bond C-N, D_2 = Max. e-e repulsion for bond C-C.

Table S5. Descriptor of the BMLR-QSAR model for the tested compounds against *Mycobacterium fortuitum*.

Entry	ID	Coefficient	*s*	*t*	Descriptor
1	0	-2.6795	0.388	-6.913	Intercept
2	D_1	0.0298467	0.003	11.054	Max. e-e repulsion for atom N
3	D_2	80.0736	3.734	21.444	Avg. electroph. react. index for atom N

$N = 11$, $n = 2$, $R^2 = 0.984$, R^2cvOO = 0.962, $F = 240.314$, $s^2 = 0.001$

Log(MIC, μM) = -2.6795 + (0.0298467 x D_1) – (80.0736 x D_2)

Table S6. Observed and estimated MIC values for the tested compounds against *Mycobacterium fortuitum* according to the BMLR-QSAR model.

Entry	Compd.	Observed MIC, mM	Estimated MIC, μM	Error[a]
1	**PZA**	81.2	78.4	2.8
2	**INH**	145.8	152.9	-7.1
3	**10a**	84.3	81.5	2.8
4	**12a**	56.1	54.3	1.8
5	**12b**	58.4	55.9	2.5
6	**12c**	56.1	54.0	2.1
7	**12d**	53.4	57.9	-4.5
8	**12e**	51.2	53.6	-2.4
9	**12g**	46.6	48.2	-1.6
10	**13**	41.1	42.0	-0.9
11	**16**	82.6	79.0	3.6

[a] Error is the difference between the observed and estimated biologically activity (MIC) values.

Table S7. Molecular descriptor values of the BMLR-QSAR model for the tested compounds against *Mycobacterium fortuitum* according to the BMLR-QSAR model.

Entry	Compd.	Descriptors[a]	
		D_1	D_2
1	**PZA**	147.356	0.00219
2	**INH**	142.5146	0.00762
3	**10a**	142.6322	0.00416
4	**12a**	142.502	0.00201
5	**12b**	142.4075	0.00221
6	**12c**	142.064	0.00215
7	**12d**	142.7317	0.00227
8	**12e**	142.3404	0.002
9	**12g**	144.0619	0.00078
10	**13**	137.0116	0.00266
11	**16**	137.8948	0.00576

[a] D_1 = Max. e-e repulsion for atom N, D_2 = Avg. electroph. react. index for atom N.

Table S8. Descriptor of the BMLR-QSAR model for the tested compounds against *Mycobacterium tuberculosis*.

Entry	ID	Coefficient	*s*	*t*	Descriptor
1	0	7.80777	1.172	6.660	Intercept
2	D_1	0.282997	0.030	9.543	Min. e-e repulsion for bond H-N
3	D_2	0.102738	0.014	7.207	Max. e-e repulsion for bond H-C
4	D_3	-1.69416	0.113	-14.963	Max. coulombic interaction for bond C-O

$N = 13$, $n = 3$, $R^2 = 0.965$, R^2cvOO = 0.892, $F = 82.181$, $s^2 = 0.004$

Log(% growth inhibition at 30 μg/mL) = 7.80777 + (0.282997 x D_1) + (0.102738 x D_2) – (1.69416 x D_3)

Table S9. Observed and estimated % growth inhibition at 30 μg/mL values for the tested compounds against *Mycobacterium tuberculosis* according to the BMLR-QSAR model.

Entry	Compd.	Observed % growth inhibition at 30 μg/mL	Estimated % growth inhibition at 30 μg/mL	Error[a]
1	**10a**	88.8	88.8	0.0
2	**10b**	91	73.8	17.2
3	**10c**	84.3	91.0	-6.7
4	**10d**	80.2	87.7	-7.5
5	**10e**	60.4	65.4	-5.0
6	**10g**	8.8	9.0	-0.2
7	**12a**	68.9	77.5	-8.6
8	**12b**	84.2	86.9	-2.7
9	**12c**	84.1	76.4	7.7
10	**12e**	94.8	89.5	5.3
11	**12g**	96	80.4	15.6
12	**13**	90.1	83.2	6.9
13	**16**	78.1	95.6	-17.5

[a] Error is the difference between the observed and estimated biologically activity (% growth inhibition at 30µg/mL) values.

Table S10. Molecular descriptor values of the BMLR-QSAR model for the tested compounds against *Mycobacterium tuberculosis* according to the BMLR-QSAR model.

Entry	Compd.	Descriptors[a]		
		D_1	D_2	D_3
1	**10a**	38.6098	34.3797	11.9929
2	**10b**	38.1408	34.23	11.9529
3	**10c**	38.6149	34.3592	11.9862
4	**10d**	38.1846	34.7702	11.9488
5	**10e**	38.9176	32.7731	12.0254
6	**10g**	37.3978	32.1811	12.2441
7	**12a**	36.8163	34.4923	11.735
8	**12b**	36.5537	34.548	11.6651
9	**12c**	38.0372	34.6277	11.9508
10	**12e**	37.7514	32.6895	11.7449
11	**12g**	37.3659	32.5331	11.6986
12	**13**	37.439	31.3733	11.6316
13	**16**	37.6267	31.677	11.6459

[a] D_1 = Min. e-e repulsion for bond H-N, D_2 = Max. e-e repulsion for bond H-C, D_3 = Max. coulombic interaction for bond C-O.

Table S11. Estimated/predicted activity values for the tested compounds against *M. marinum* and *M. fortuitum* mapped with the generated 3D-pharmacophore models.

Entry	Compd.	*Mycobacterium marinum*		*Mycobacterium fortuitum*	
		Observed MIC, μM	Estimated MIC, μM	Observed MIC, μM	Estimated MIC, μM
1	**PZA**	81.2	138.5	81.2	88.2
2	**INH**	72.9	77.4	145.8	96.7
3	**10a**	84.3	83.2	84.3	91.5
4	**12a**	56.1	46.9	56.1	52.7
5	**12b**	58.4	43.7	58.4	56.9
6	**12c**	56.1	45.2	56.1	52.3
7	**12d**	26.7	33.4	53.4	50.6
8	**12e**	51.2	39.3	51.2	48.0
9	**12g**	46.6	38.1	46.6	44.7
10	**13**	41.1	67.4	41.1	75.7
11	**16**	82.6	65.2	82.6	75.8

Table S12. Estimated/predicted activity values for the tested compounds against *M. tuberculosis* mapped with the generated 3D-pharmacophore model.

Entry	Compd.	Observed (% growth inhibition at 30 μg/mL)	Estimated (% growth inhibition at 30 μg/mL)
1	**10a**	88.8	61.6
2	**10b**	91.0	59.2
3	**10c**	84.3	81.2
4	**10d**	80.2	63.6
5	**10e**	60.4	50.7
6	**10g**	8.8	20.2
7	**12a**	68.9	88.3
8	**12b**	84.2	92.4
9	**12c**	84.1	88.7
10	**12e**	94.8	92.0
11	**12g**	96.0	95.8
12	**13**	90.1	89.6
13	**16**	78.1	89.4

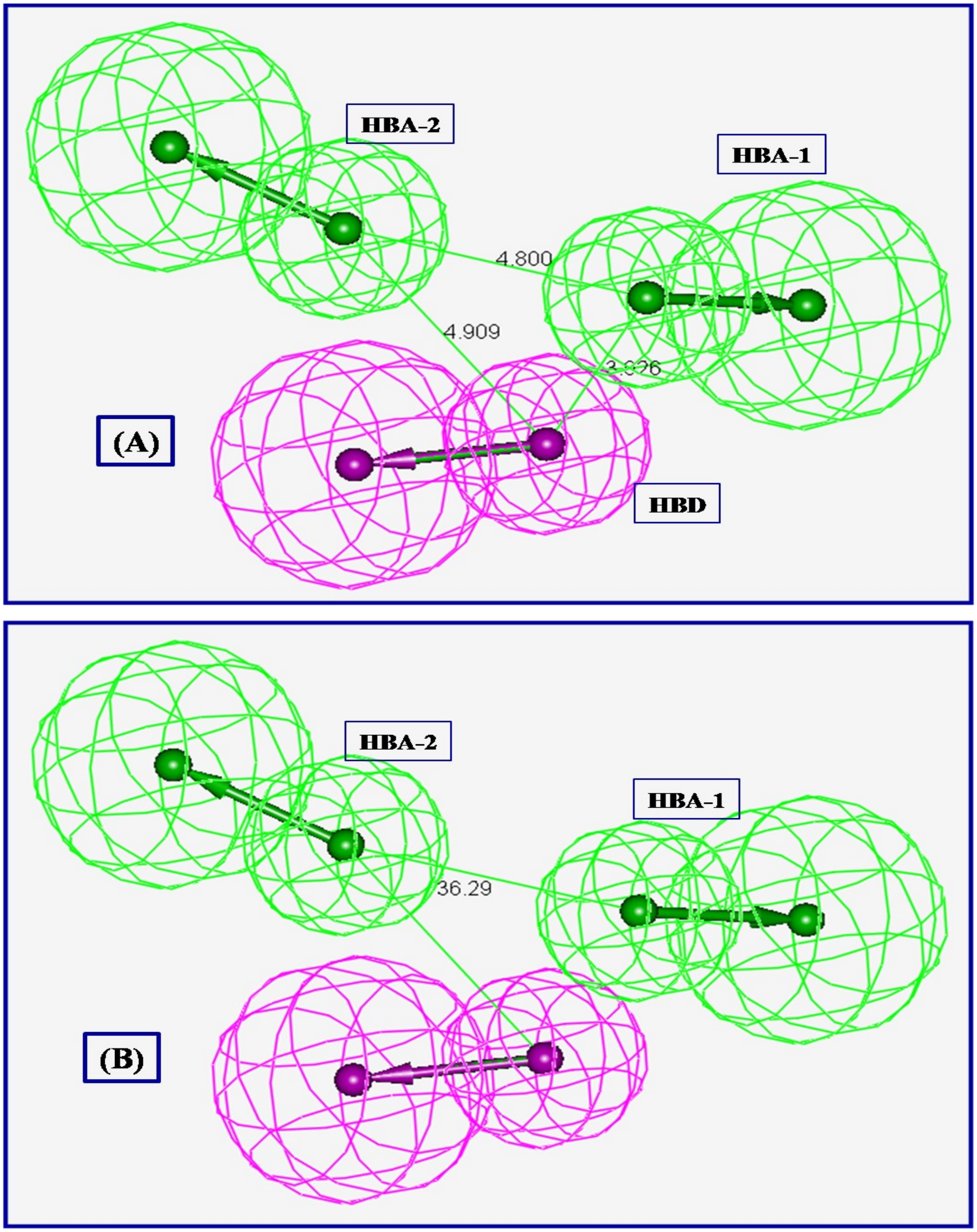

Fig. S1. (A) Constraint distances “HBA-1 – HBA-2 = 4.800, HBA-1 – HBD = 3.026, HBA-2 – HBD = 4.909 Å” and (B) constraint angles “HBA-1 – HBA-2 – HBD = 36.29 °” of the generated 3D-pharmacophore for the synthesized bio-active compounds against *Mycobacterium marinum* which contains two hydrogen bonding acceptors (HBA-1, HBA-2; green) and one hydrogen bonding donor (HBD; purple).

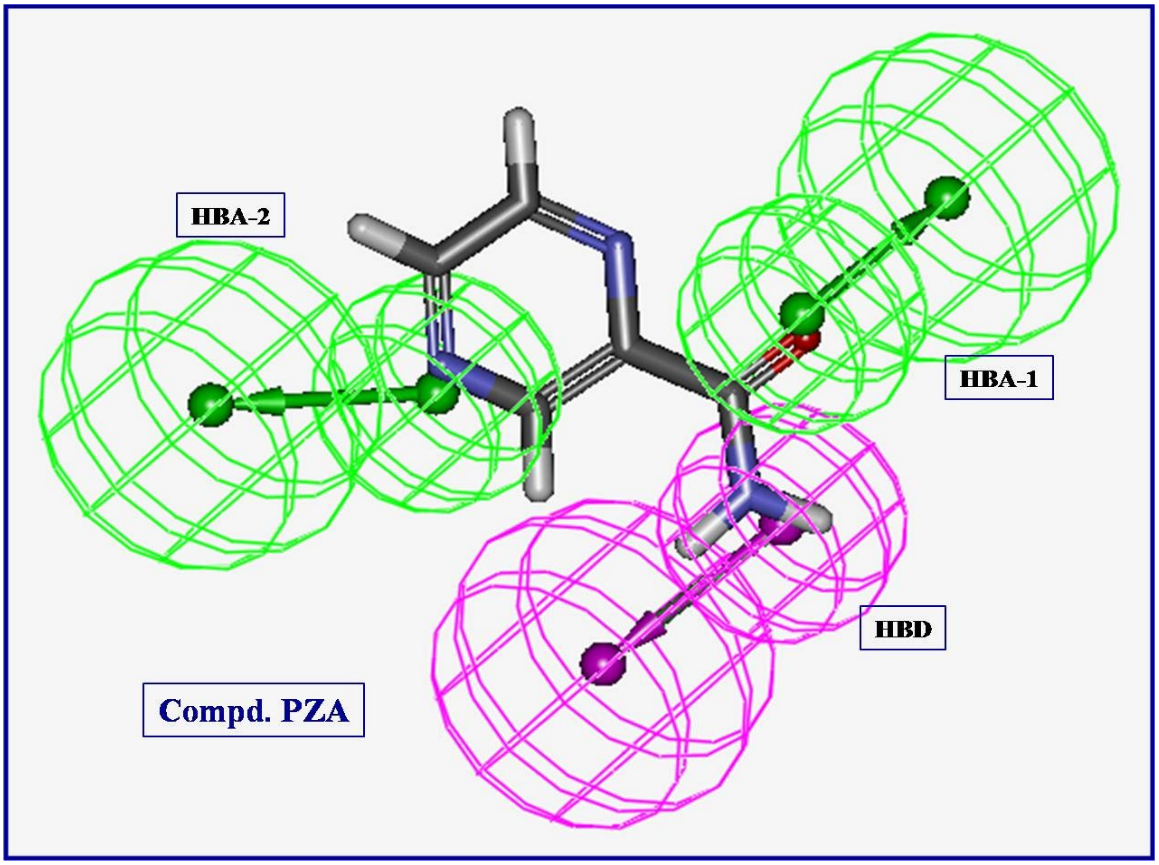
HBA-2
HBA-1
HBD
Compd. PZA

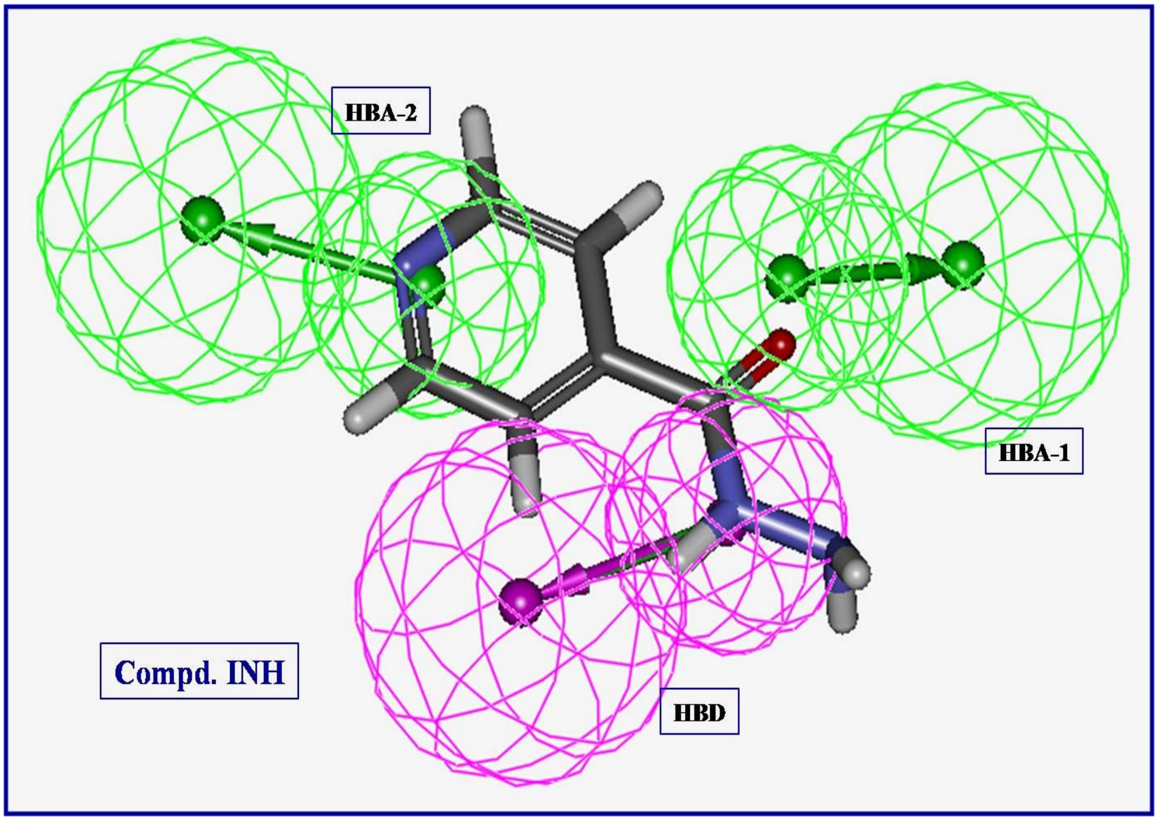
HBA-2
HBA-1
HBD
Compd. INH

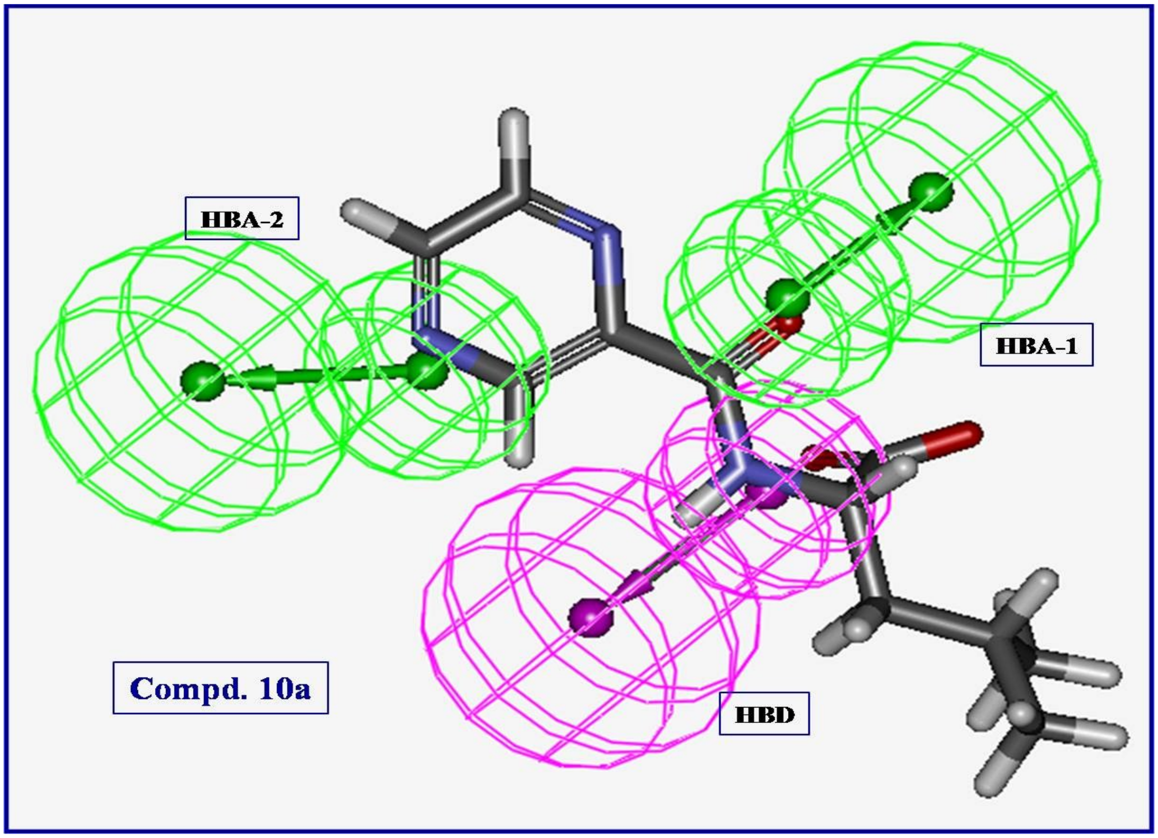
HBA-2
HBA-1
HBD
Compd. 10a

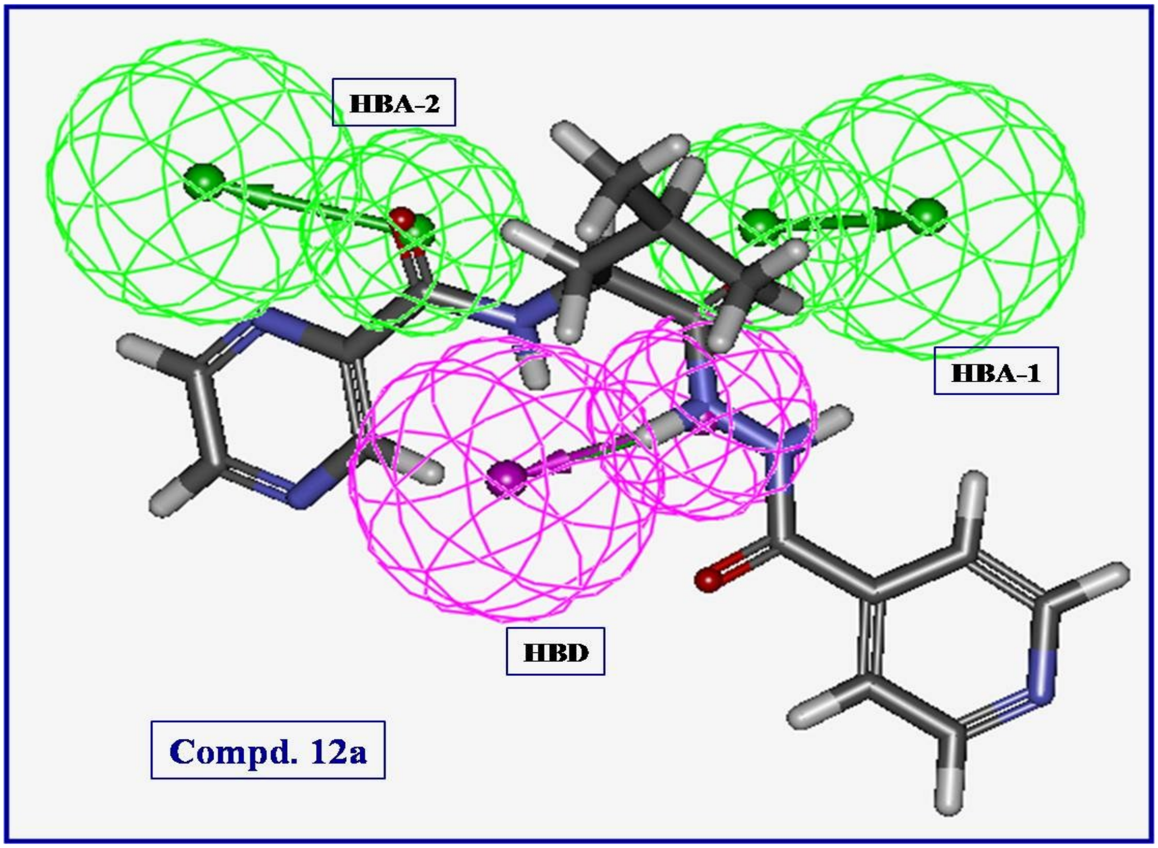
HBA-2
HBA-1
HBD
Compd. 12a

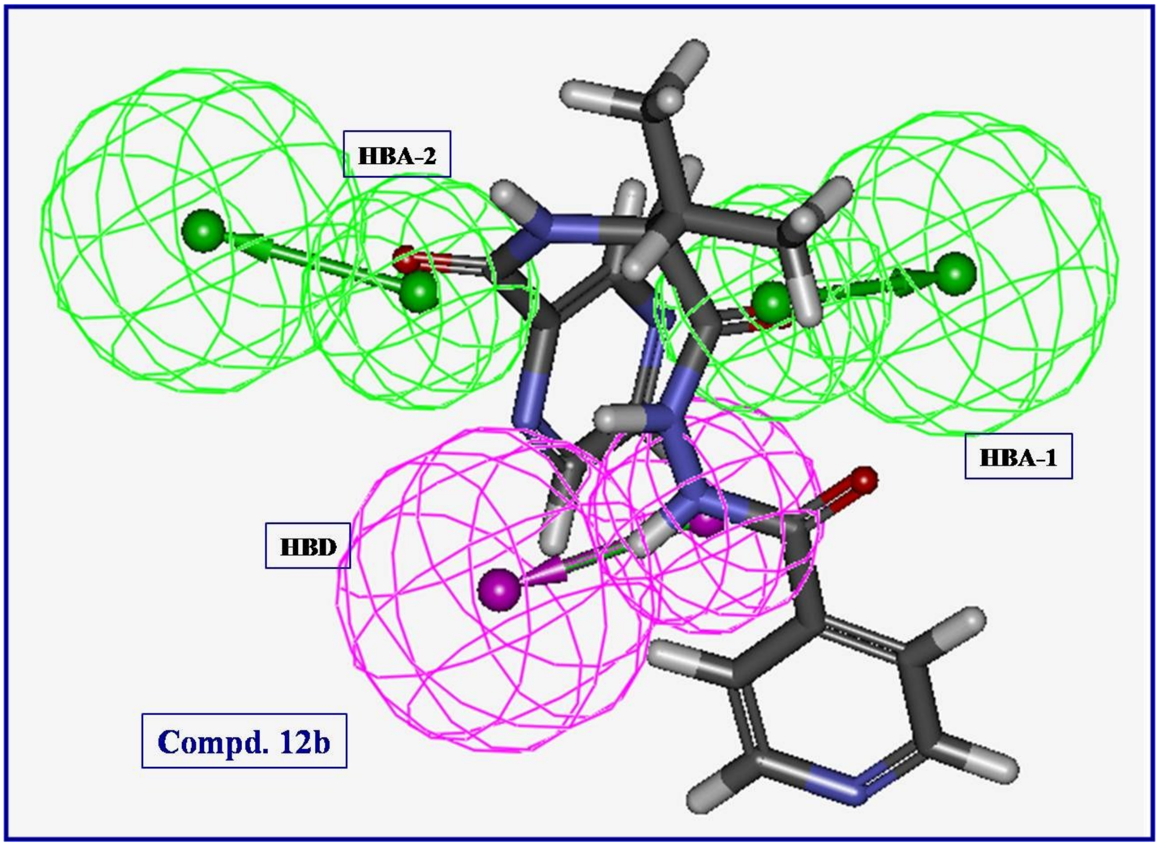
HBA-2
HBA-1
HBD
Compd. 12b

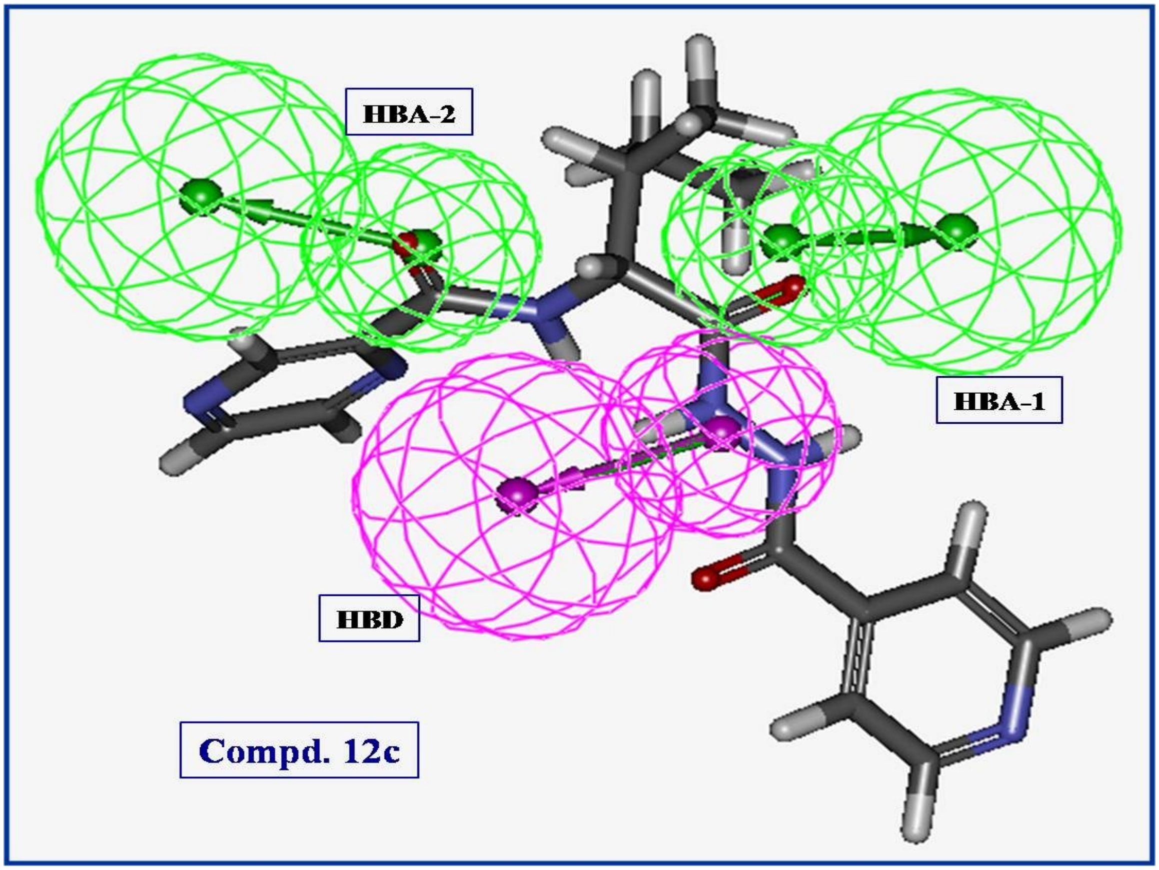
HBA-2
HBA-1
HBD
Compd. 12c

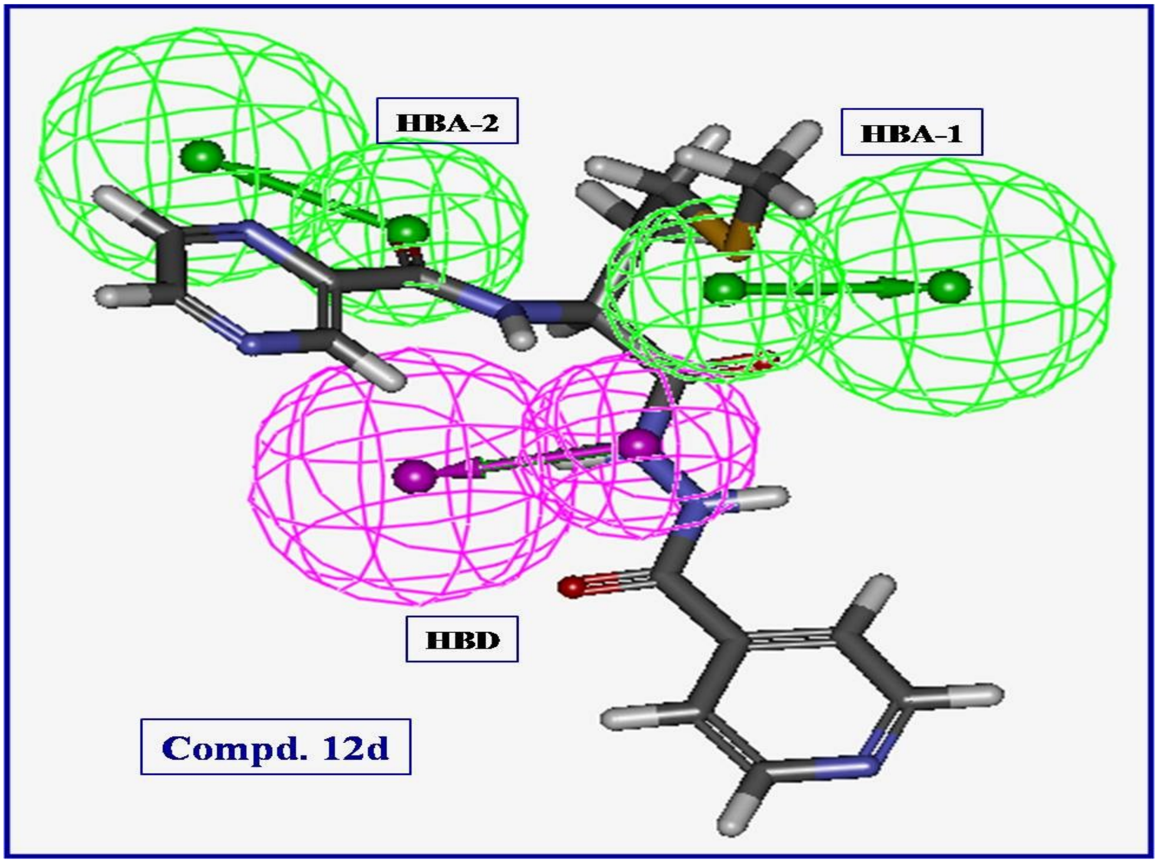
HBA-2
HBA-1
HBD
Compd. 12d

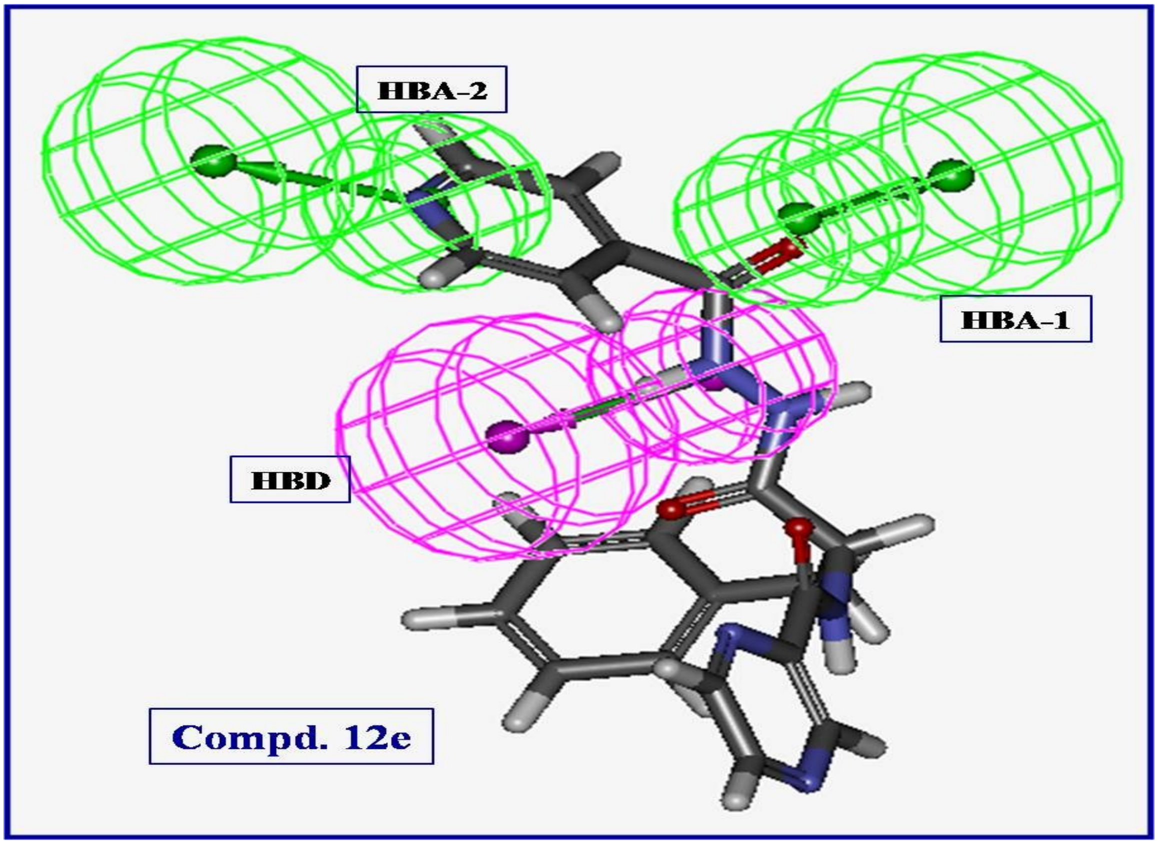
HBA-2
HBA-1
HBD
Compd. 12e

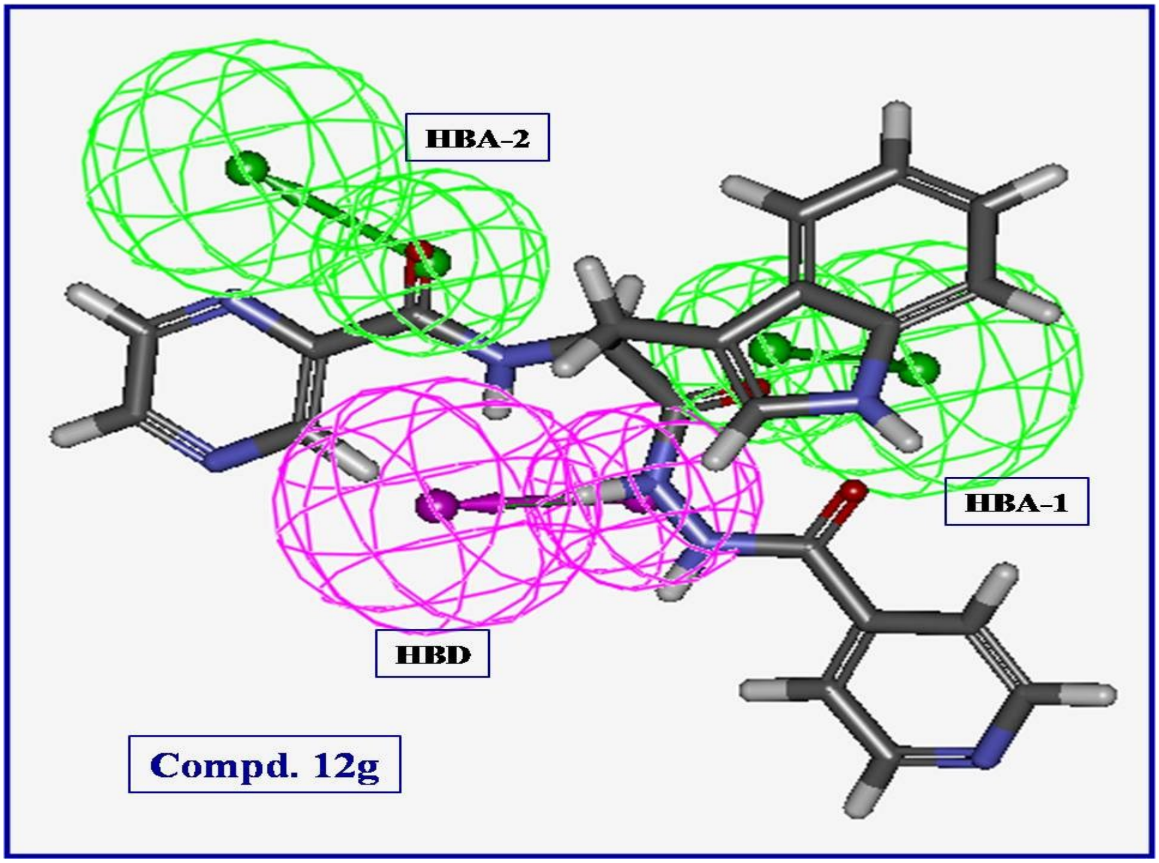
HBA-2
HBA-1
HBD
Compd. 12g

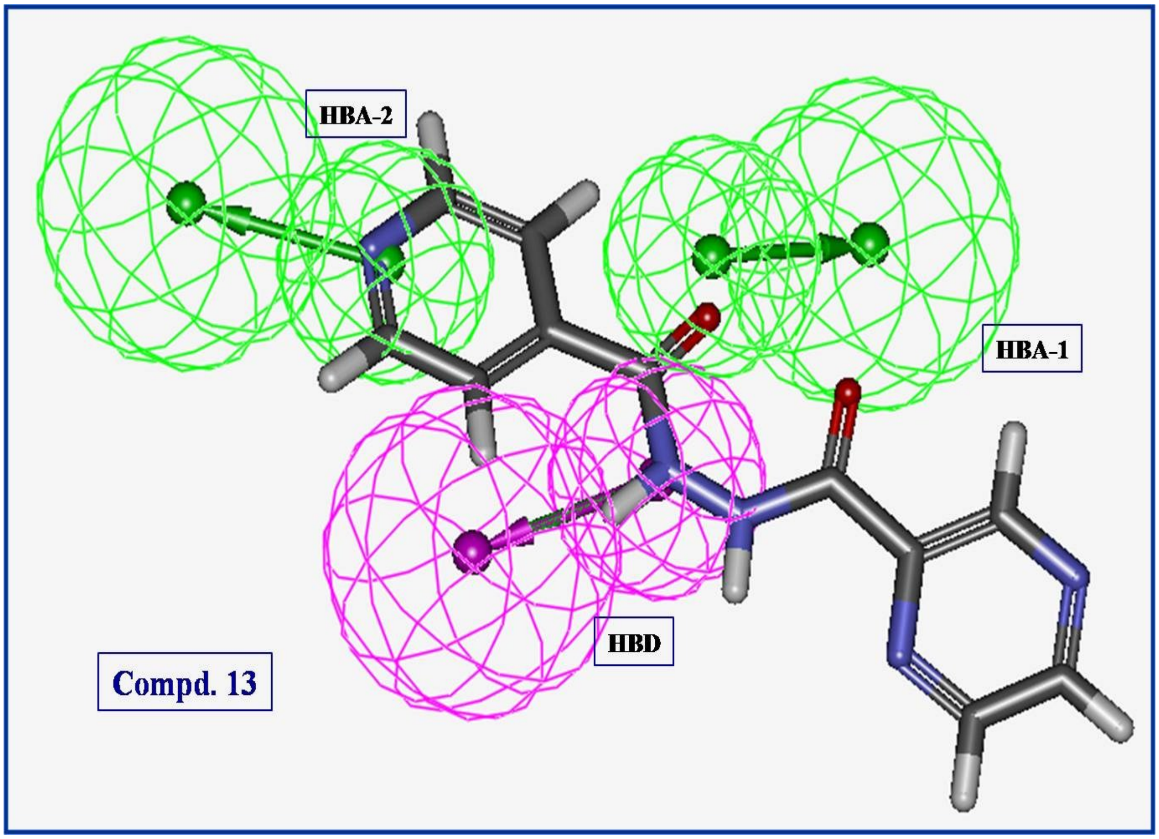
HBA-2
HBA-1
HBD
Compd. 13

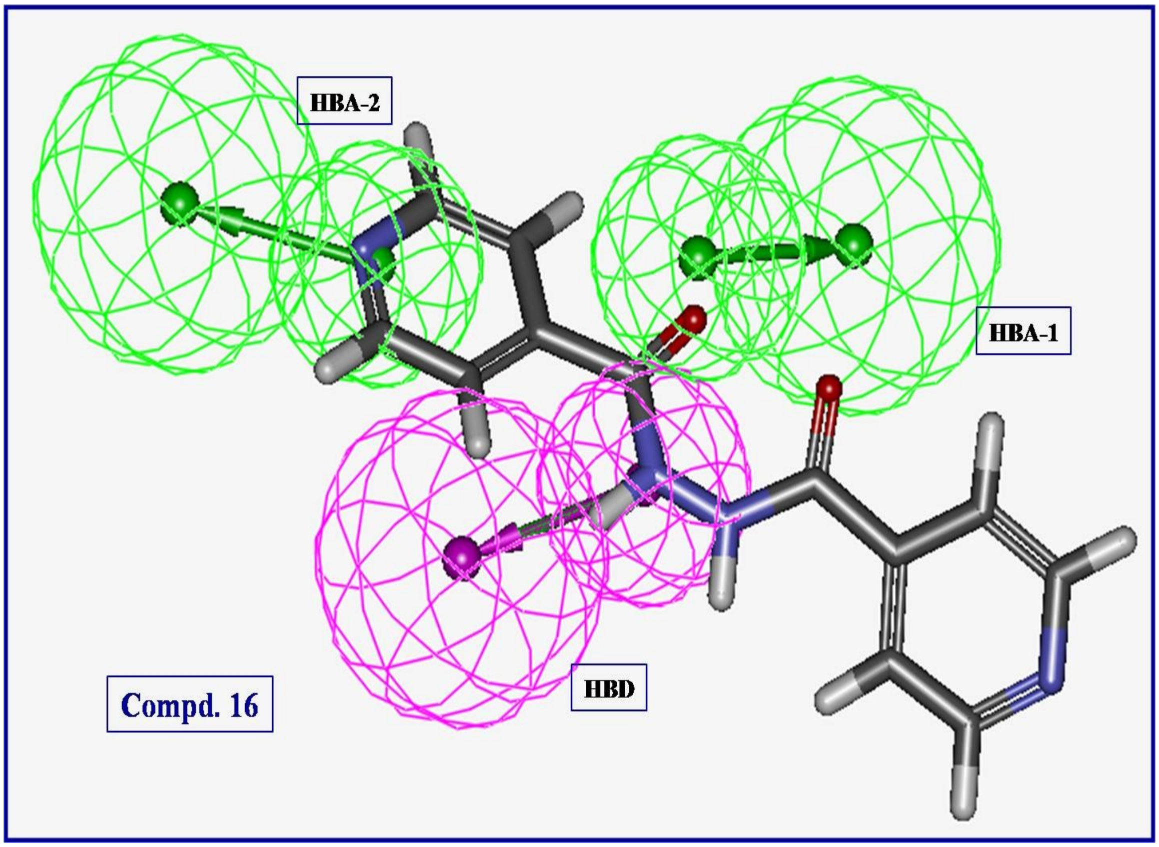

Fig. S2. 3D-pharmacophore mapped on the synthesized bio-active compounds against *Mycobacterium marinum*.

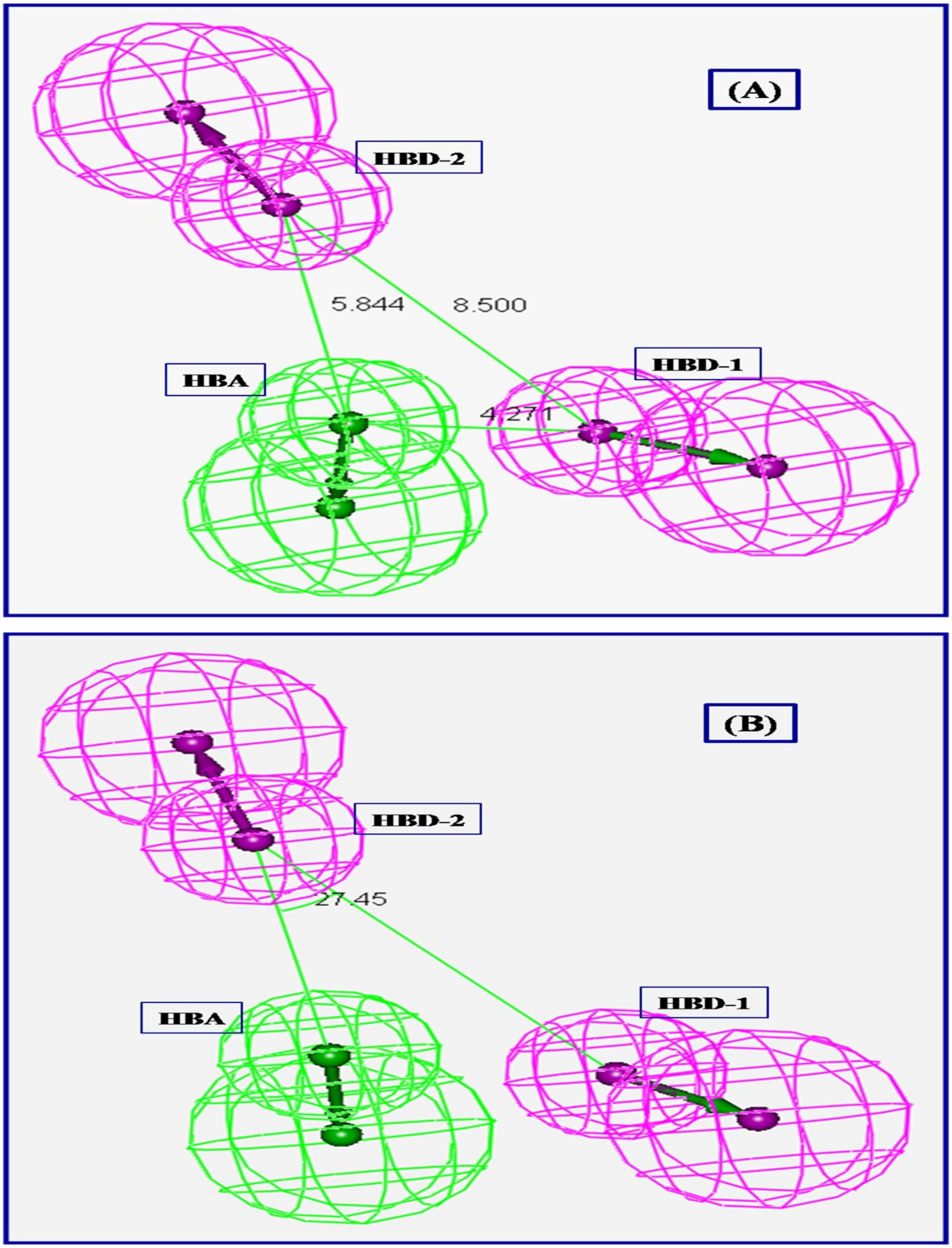

Fig. S3. (A) Constraint distances "HBD-1 – HBD-2 = 8.500, HBD-1 – HBA = 4.271, HBD-2 – HBA = 5.844 Å" and (B) constraint angles "HBD-1 – HBD-2 – HBA = 27.45 °" of the generated 3D-pharmacophore for the synthesized bio-active compounds against *Mycobacterium fortuitum* which contains two hydrogen bonding donors (HBD-1, HBD-2; purple) and one hydrogen bonding acceptor (HBA; green).

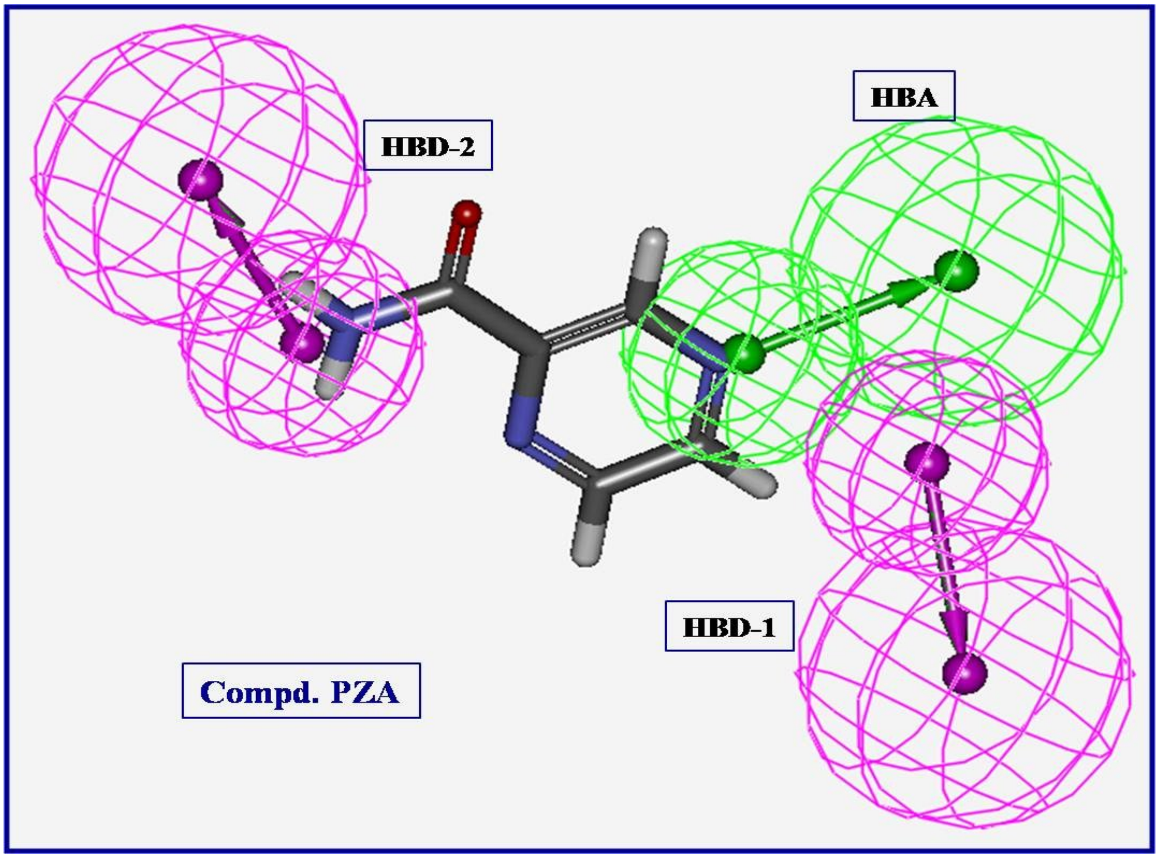
HBA
HBD-2
HBD-1
Compd. PZA

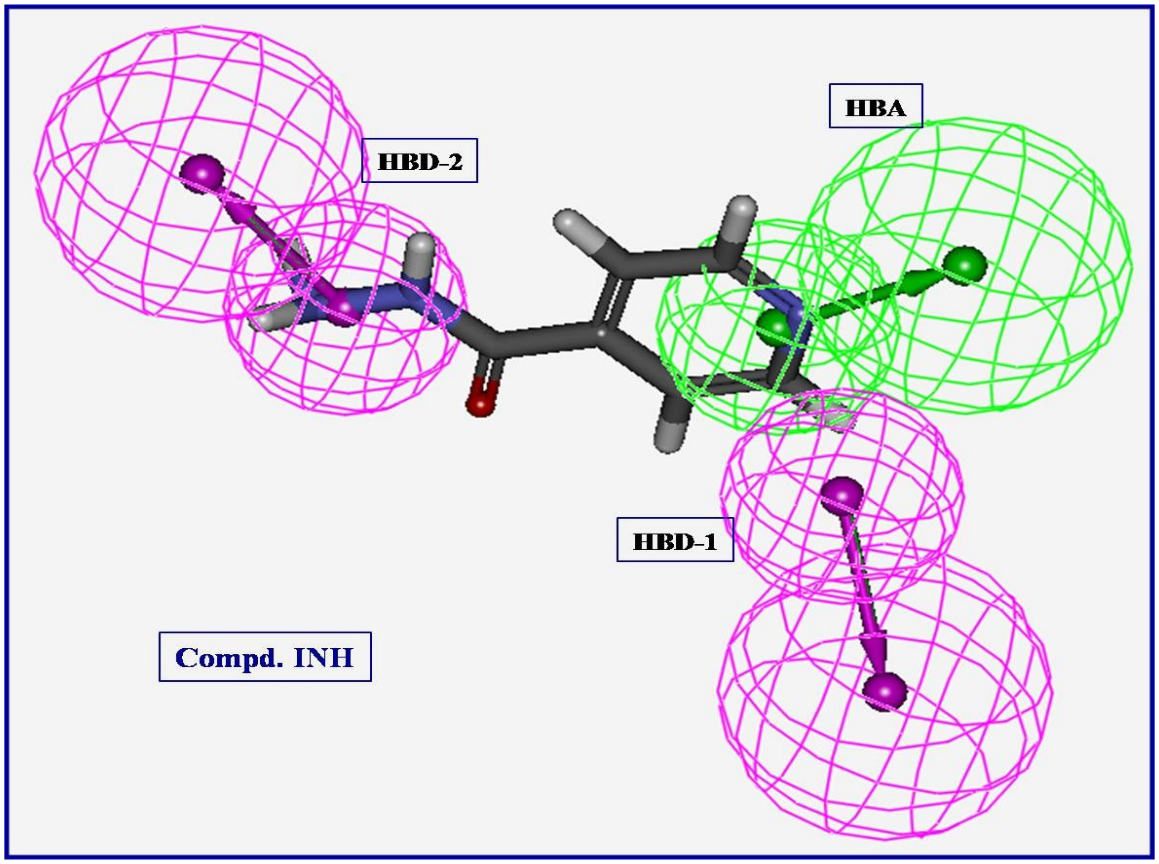
HBA
HBD-2
HBD-1
Compd. INH

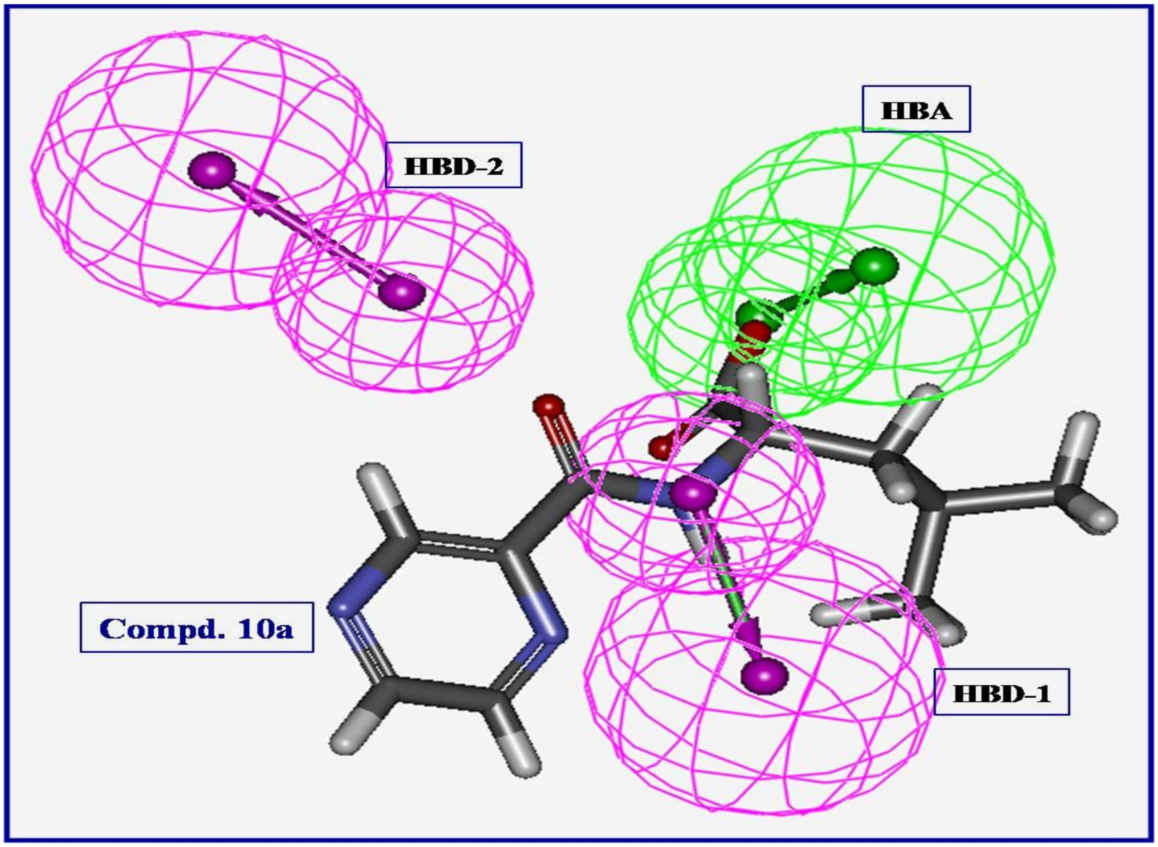
HBA
HBD-2
Compd. 10a
HBD-1

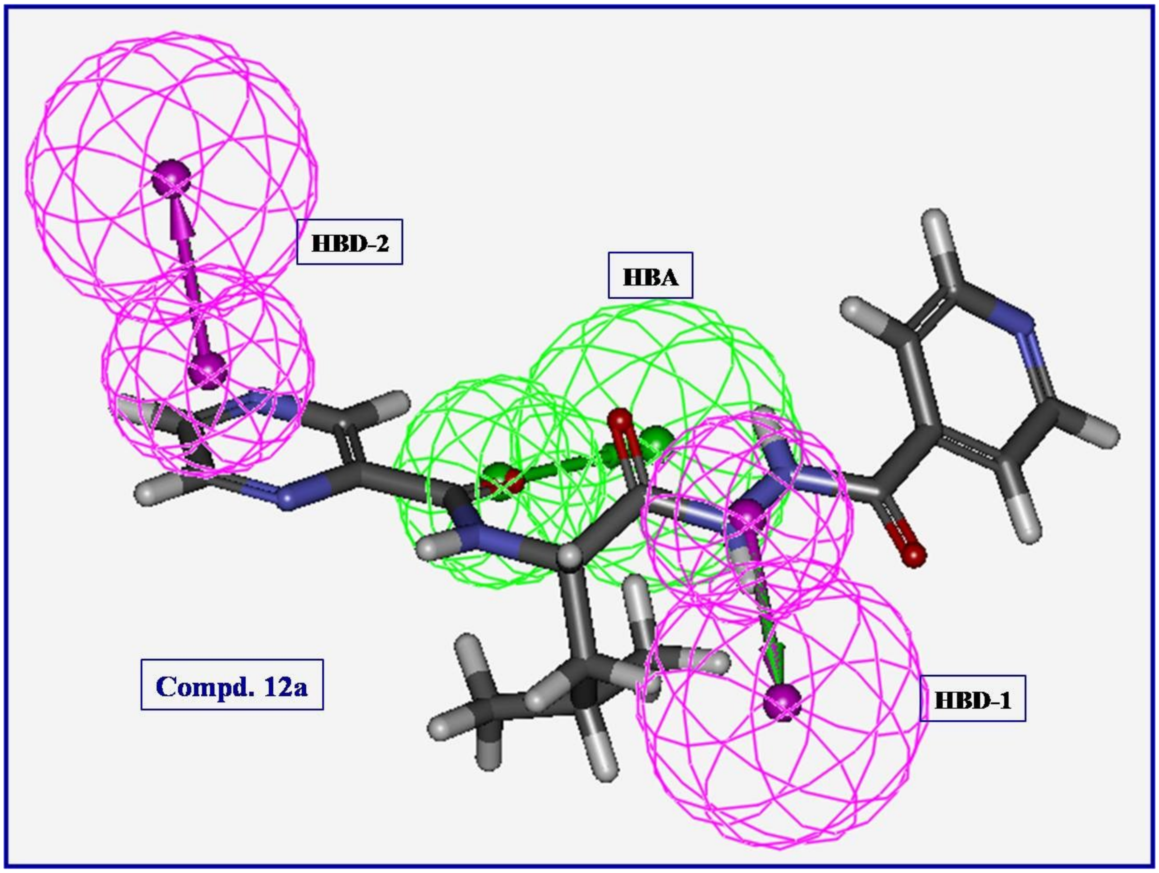
HBD-2
HBA
Compd. 12a
HBD-1

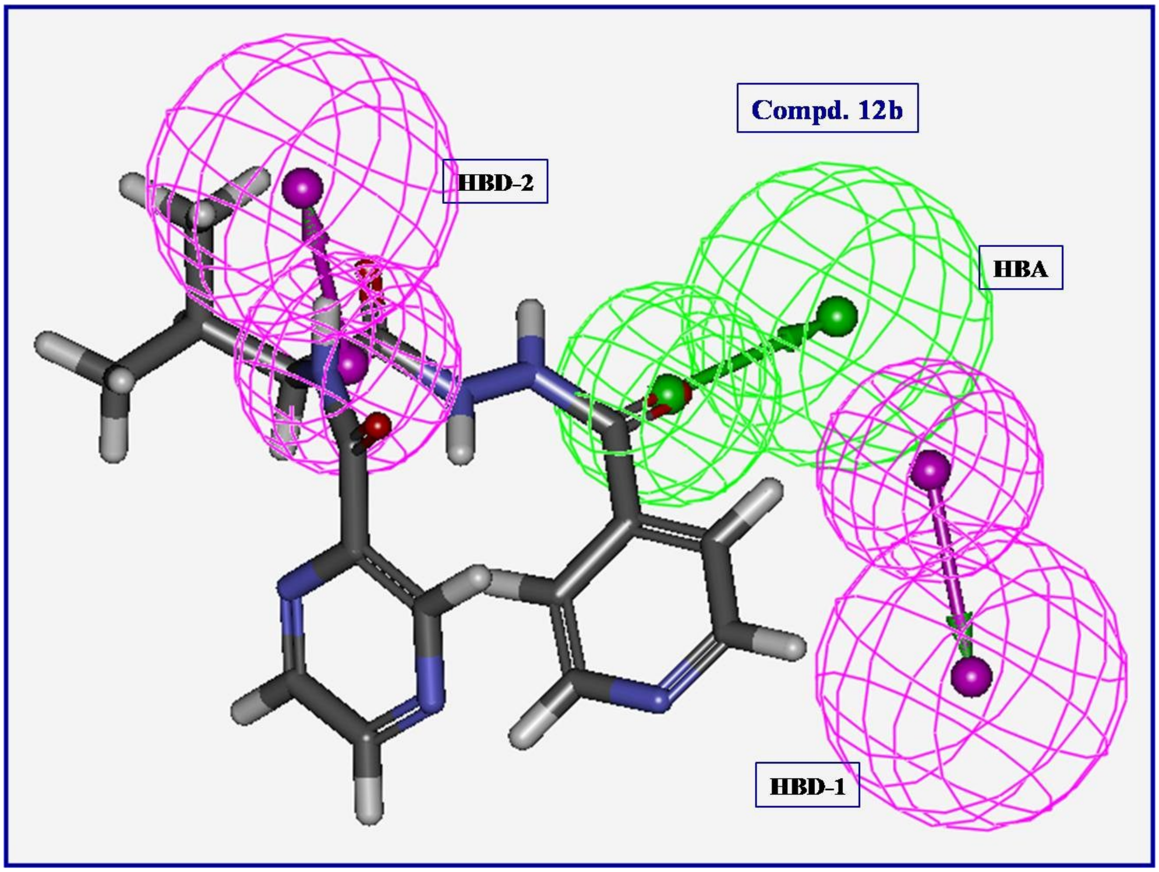
Compd. 12b
HBD-2
HBA
HBD-1

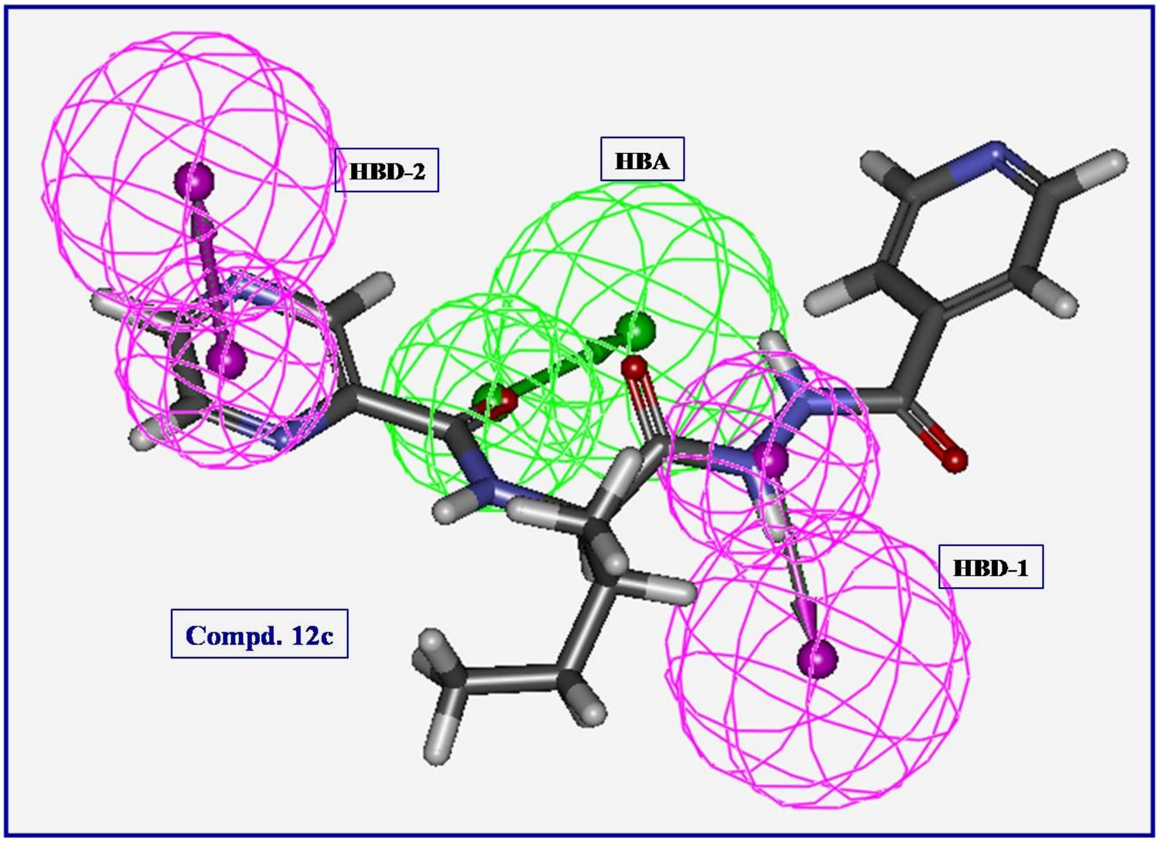
HBD-2
HBA
HBD-1
Compd. 12c

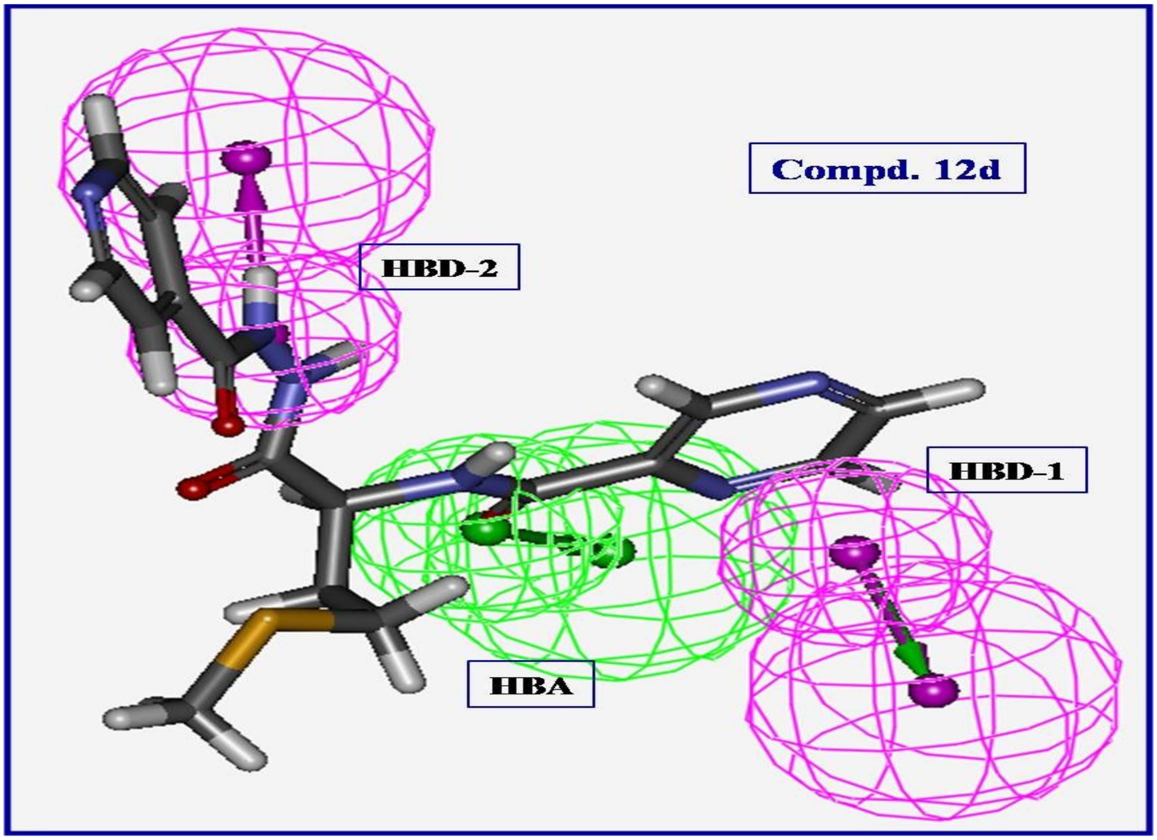
Compd. 12d
HBD-2
HBD-1
HBA

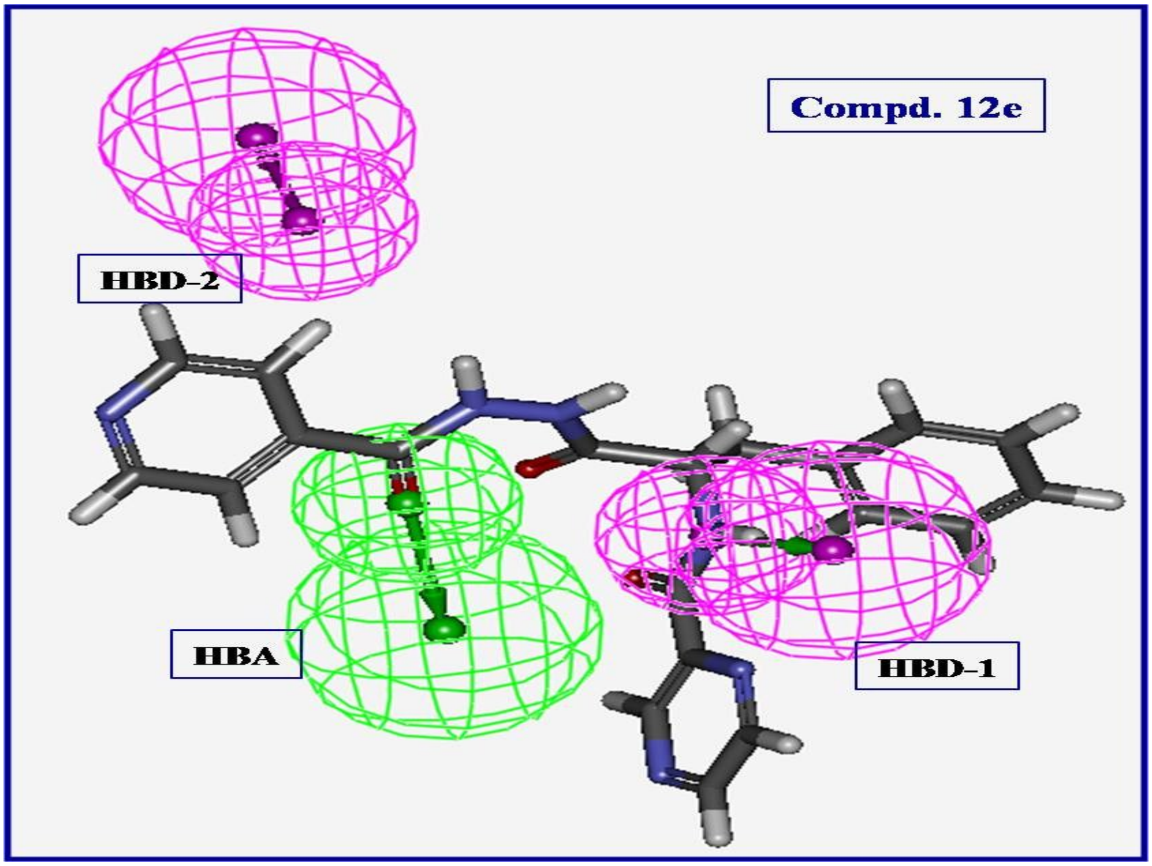
Compd. 12e
HBD-2
HBA
HBD-1

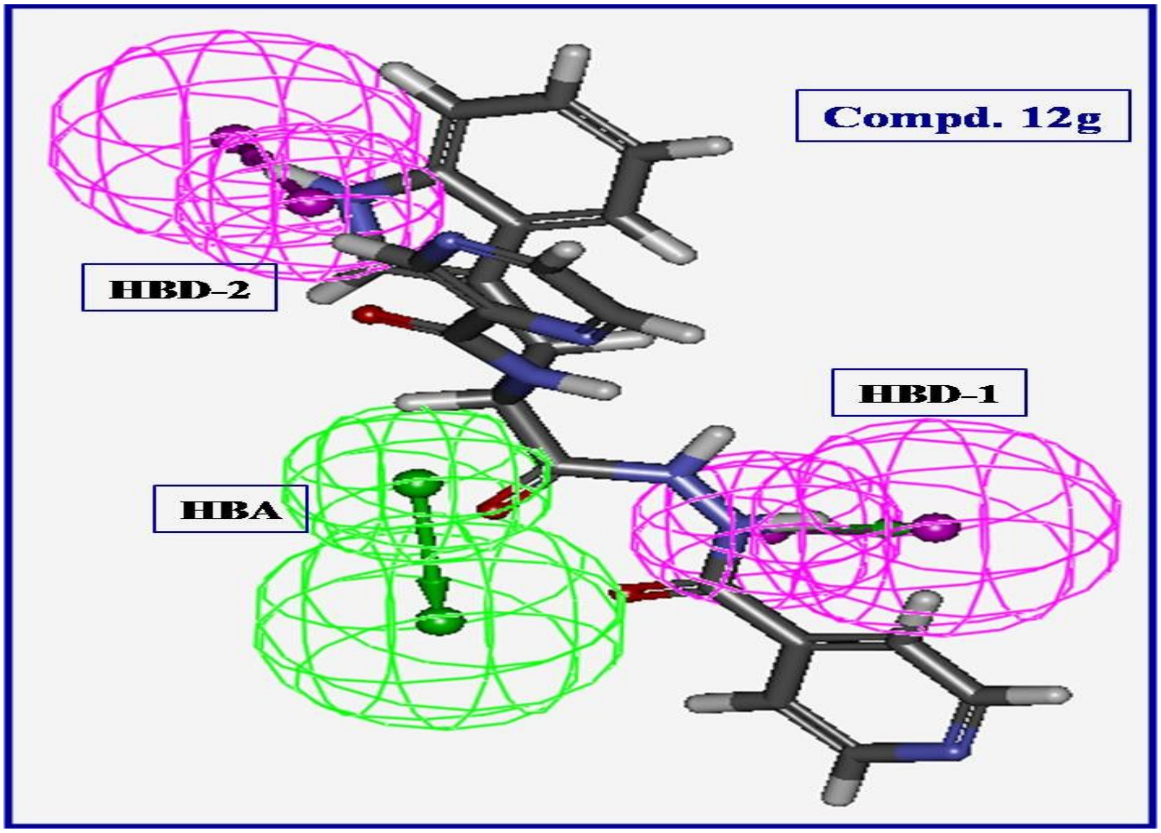
Compd. 12g
HBD-2
HBD-1
HBA

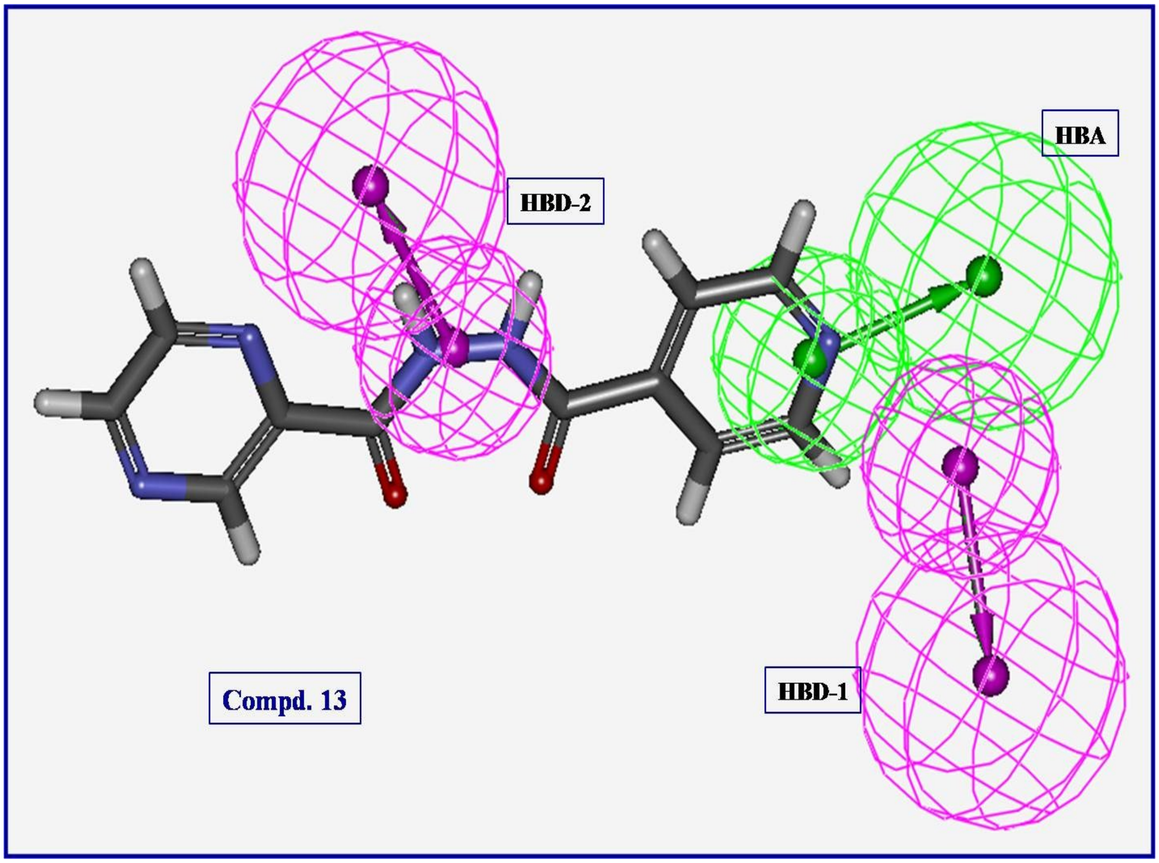
HBA
HBD-2
Compd. 13
HBD-1

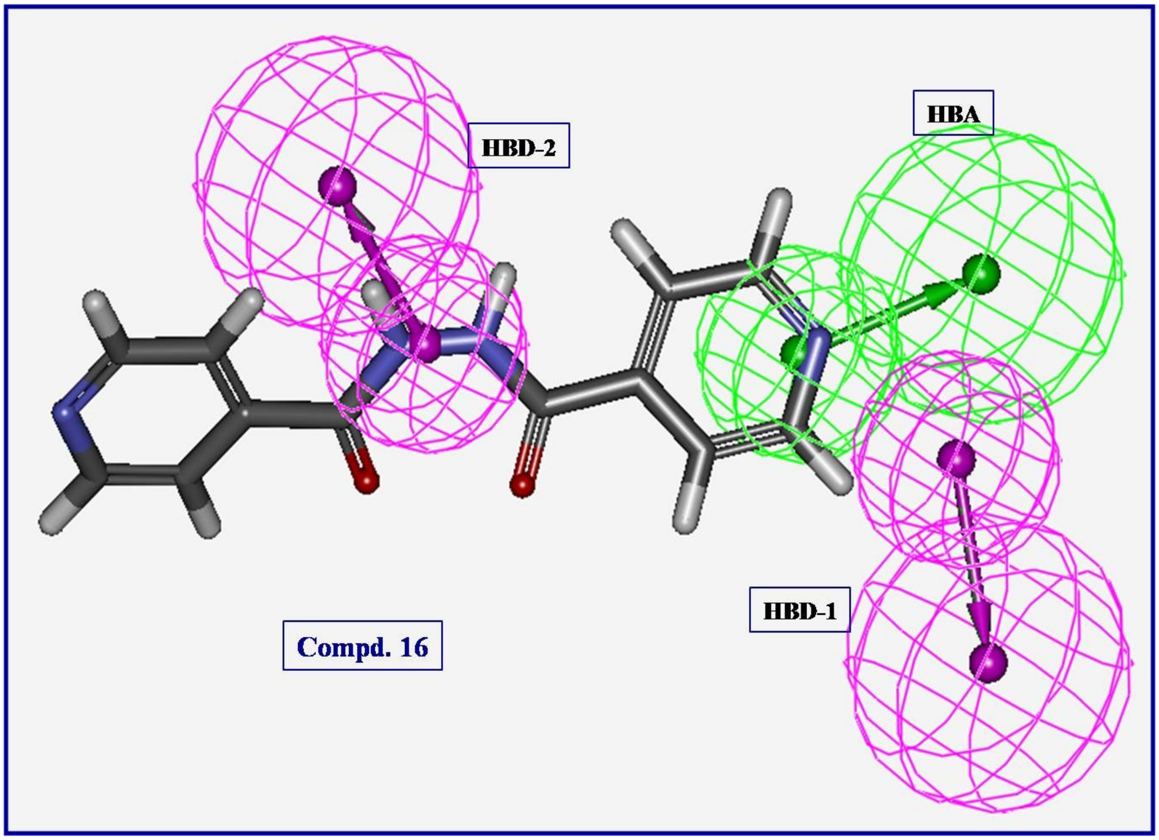

Fig. S4. 3D-pharmacophore mapped on the synthesized bio-active compounds against *Mycobacterium fortuitum*.

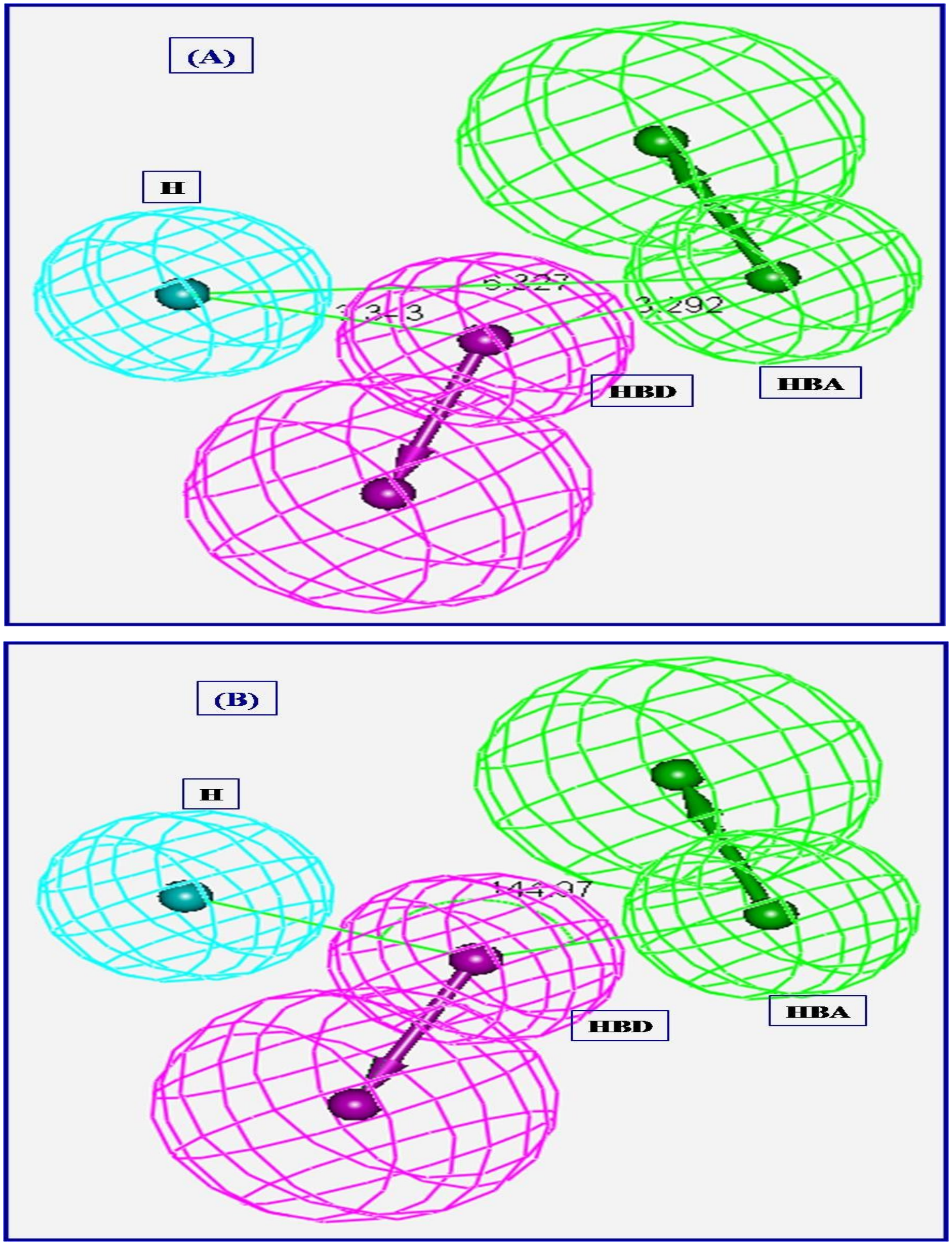

Fig. S5. (A) Constraint distances “HBA – HBD = 3.292, HBA – H = 6.327, HBD – H = 3.343 Å” and (B) constraint angles “HBA – HBD – H = 144.97 °” of the generated 3D-pharmacophore for the synthesized bio-active compounds against *Mycobacterium tuberculosis* which contains hydrogen bonding acceptor (HBA; green), hydrogen bonding donor (HBD; purple) and hydrophobic (H; light blue).

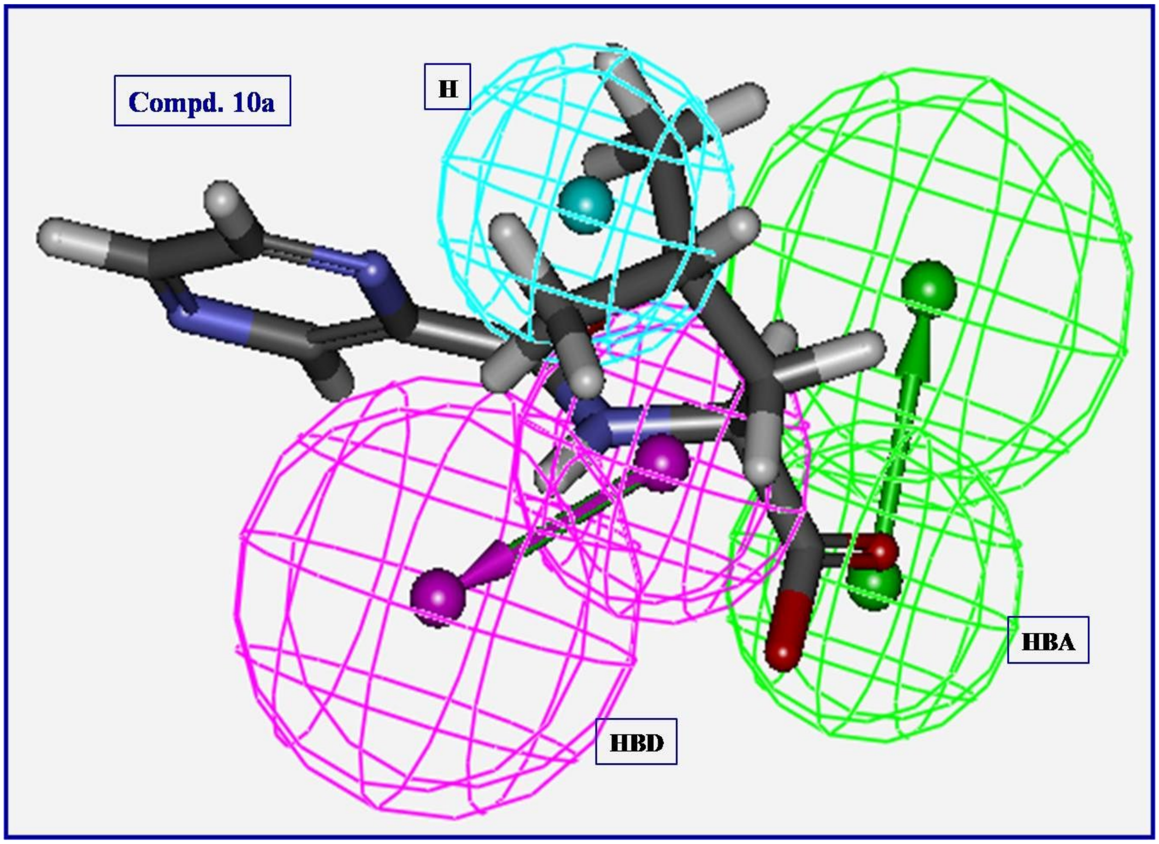
Compd. 10a
H
HBA
HBD

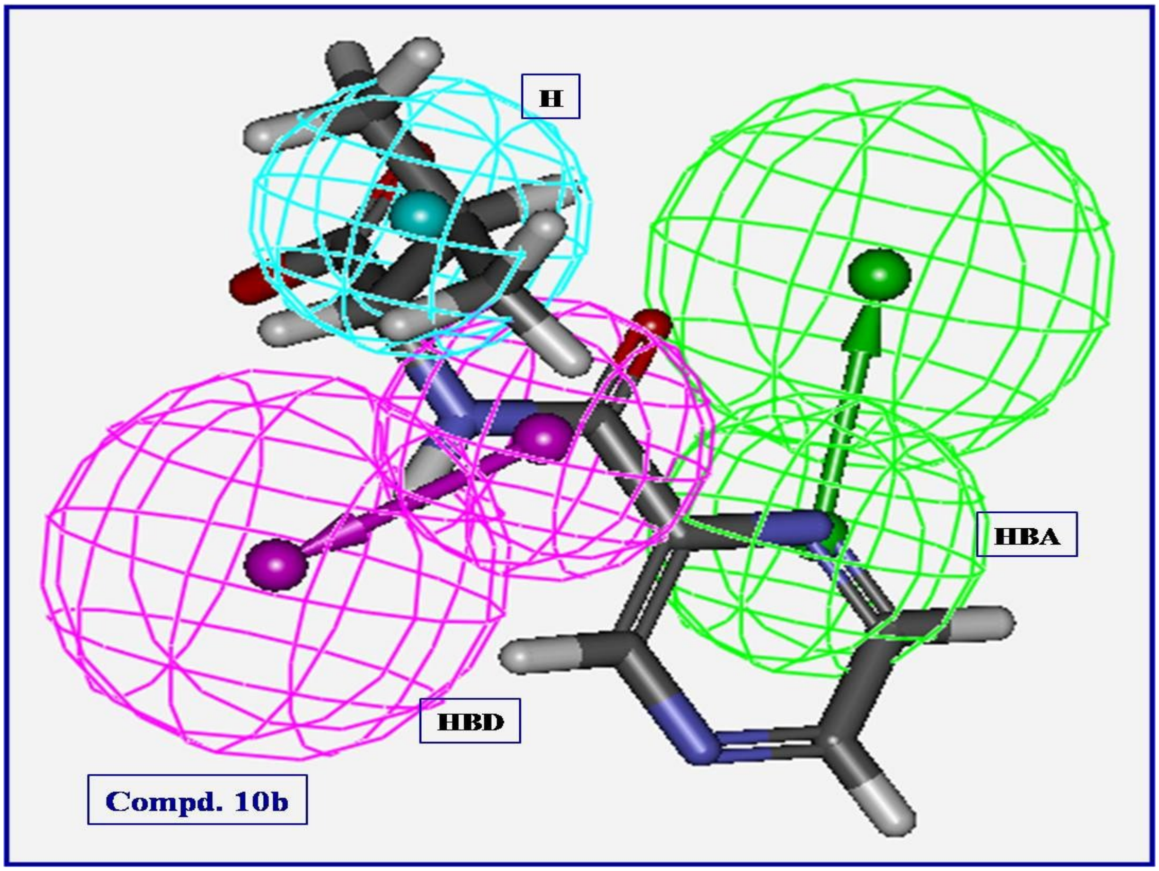
H
HBA
HBD
Compd. 10b

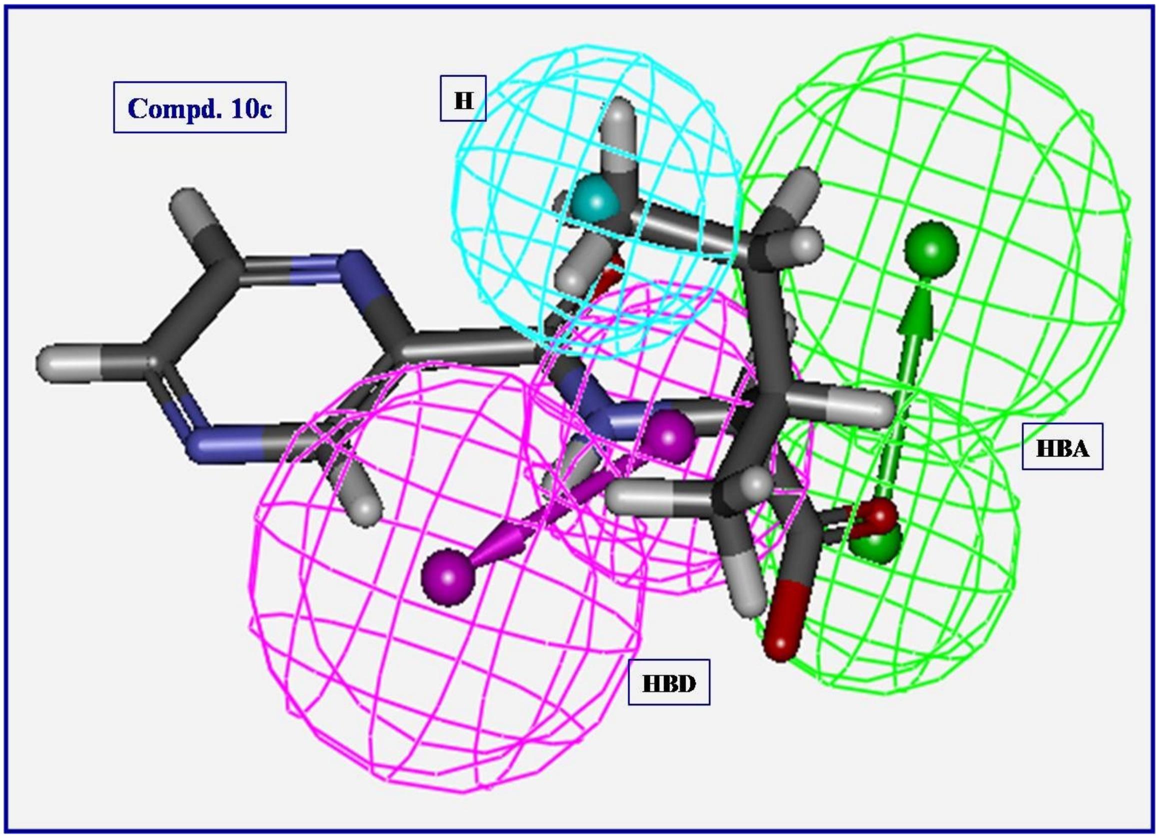
Compd. 10c
H
HBA
HBD

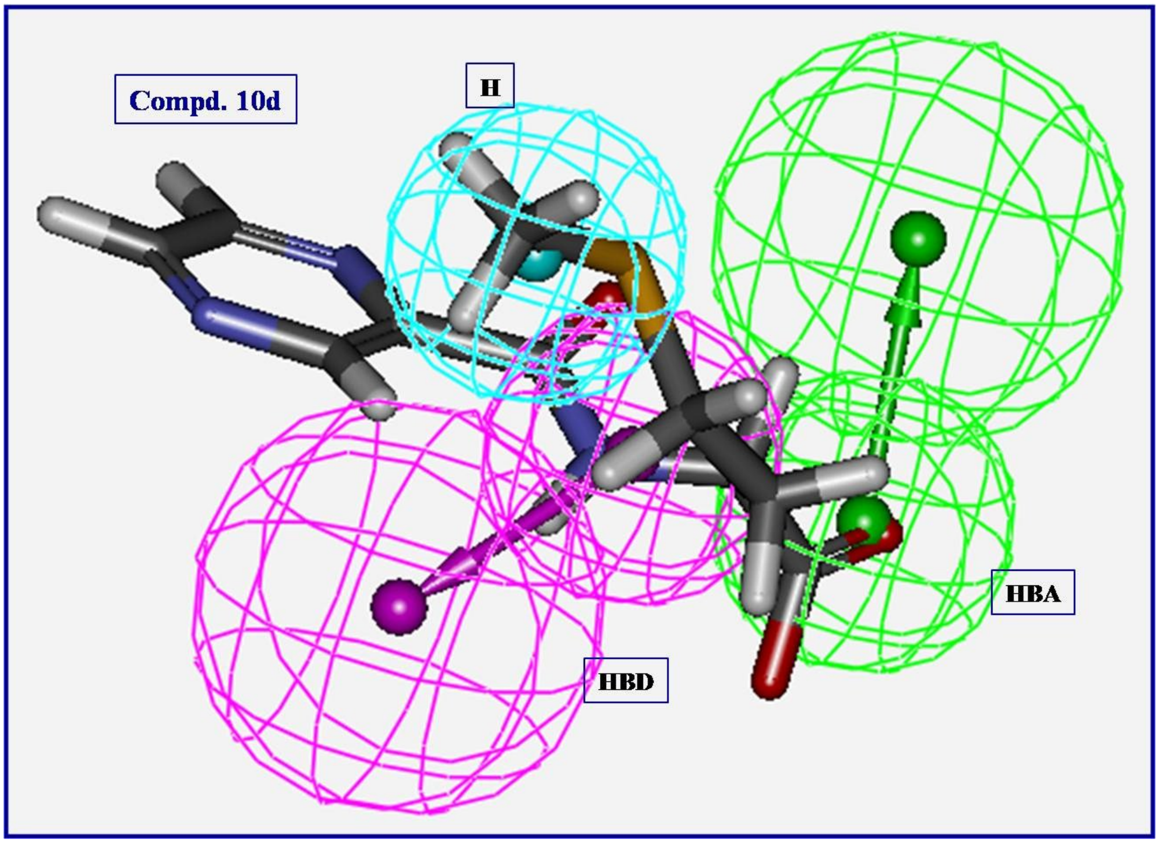
Compd. 10d
H
HBA
HBD

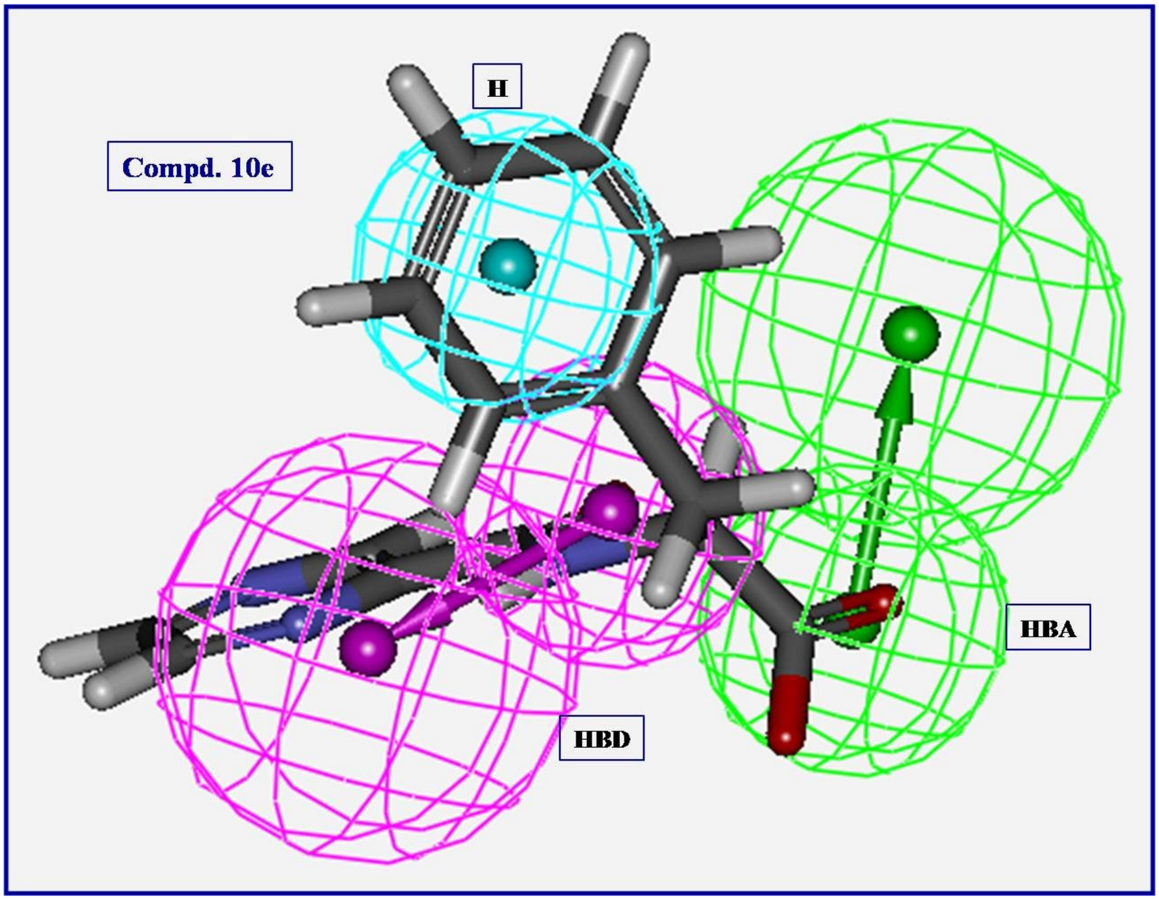
H
Compd. 10e
HBA
HBD

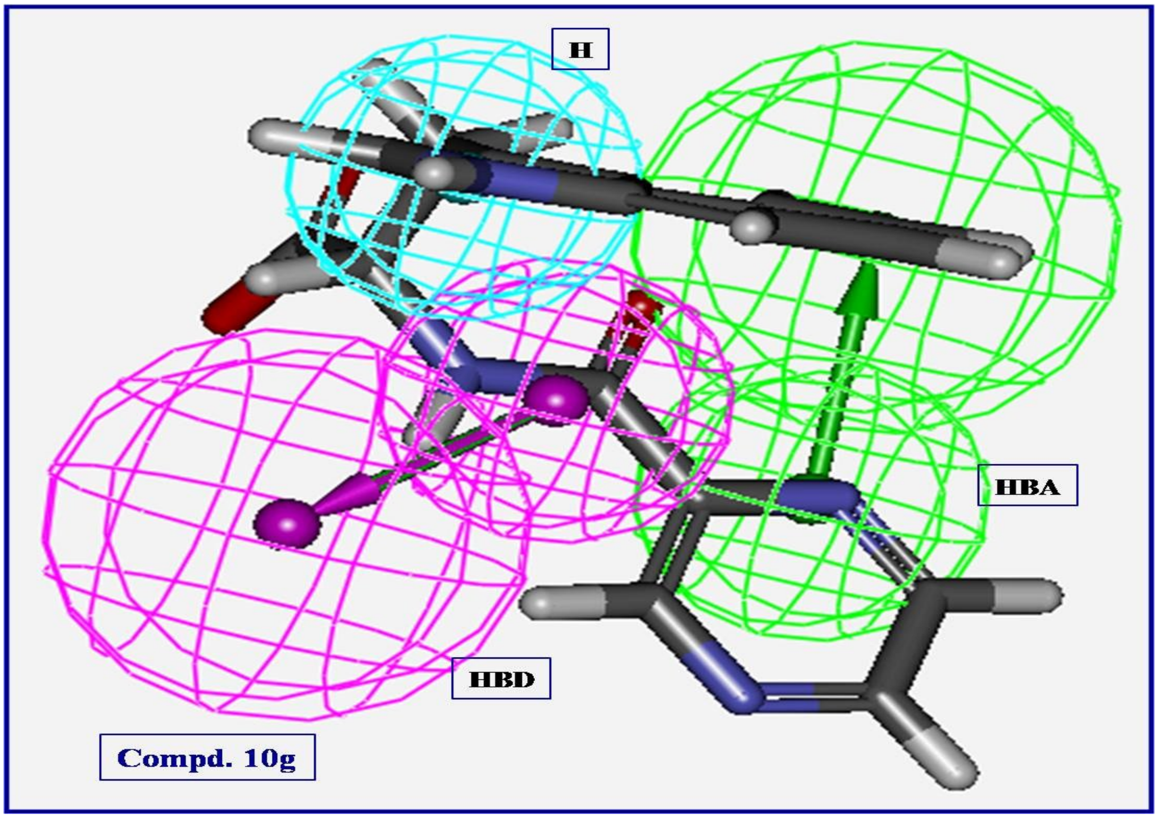
H
HBA
HBD
Compd. 10g

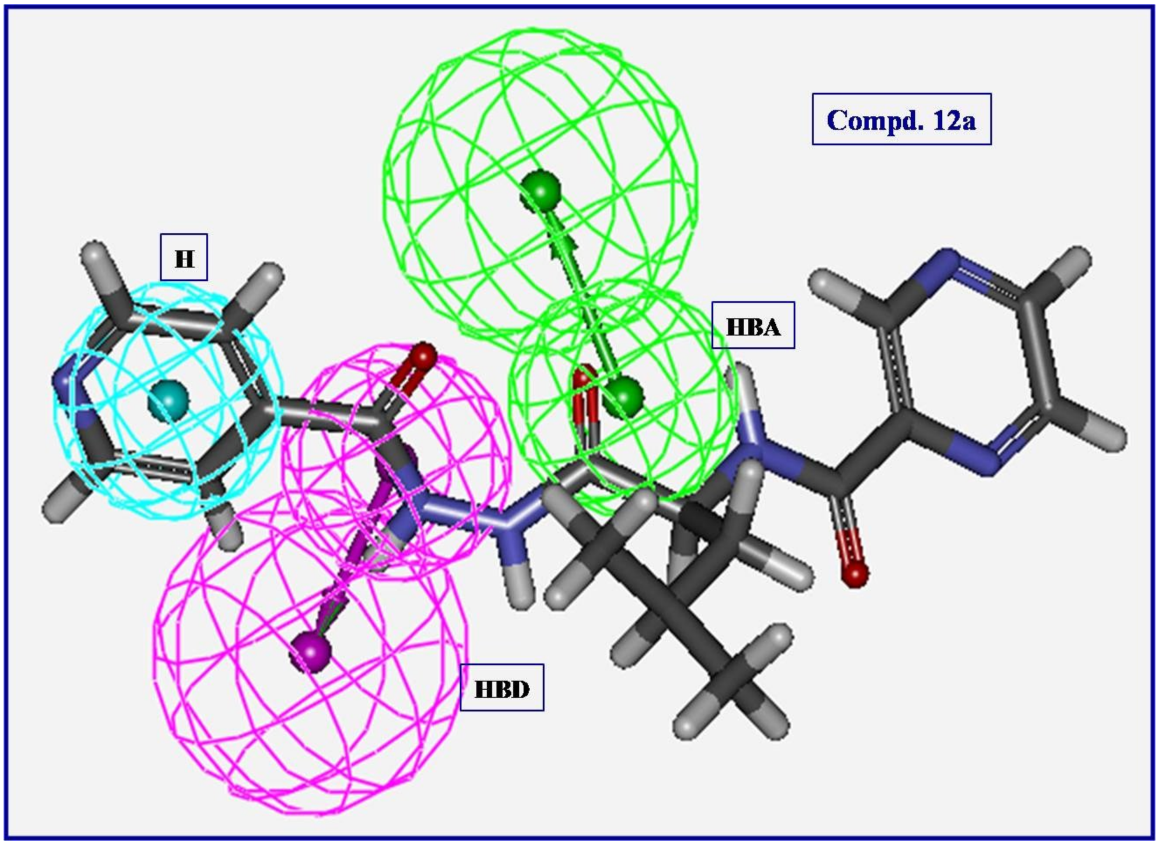
Compd. 12a
H
HBA
HBD

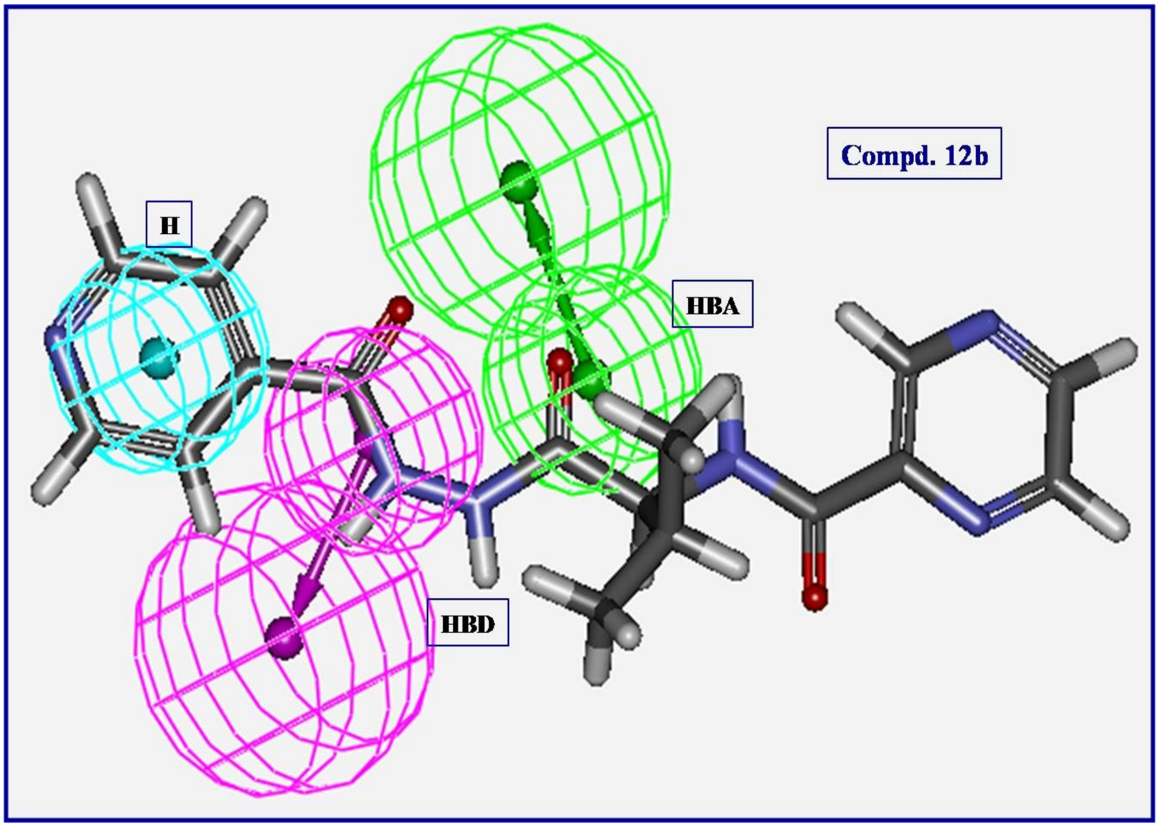
Compd. 12b
H
HBA
HBD

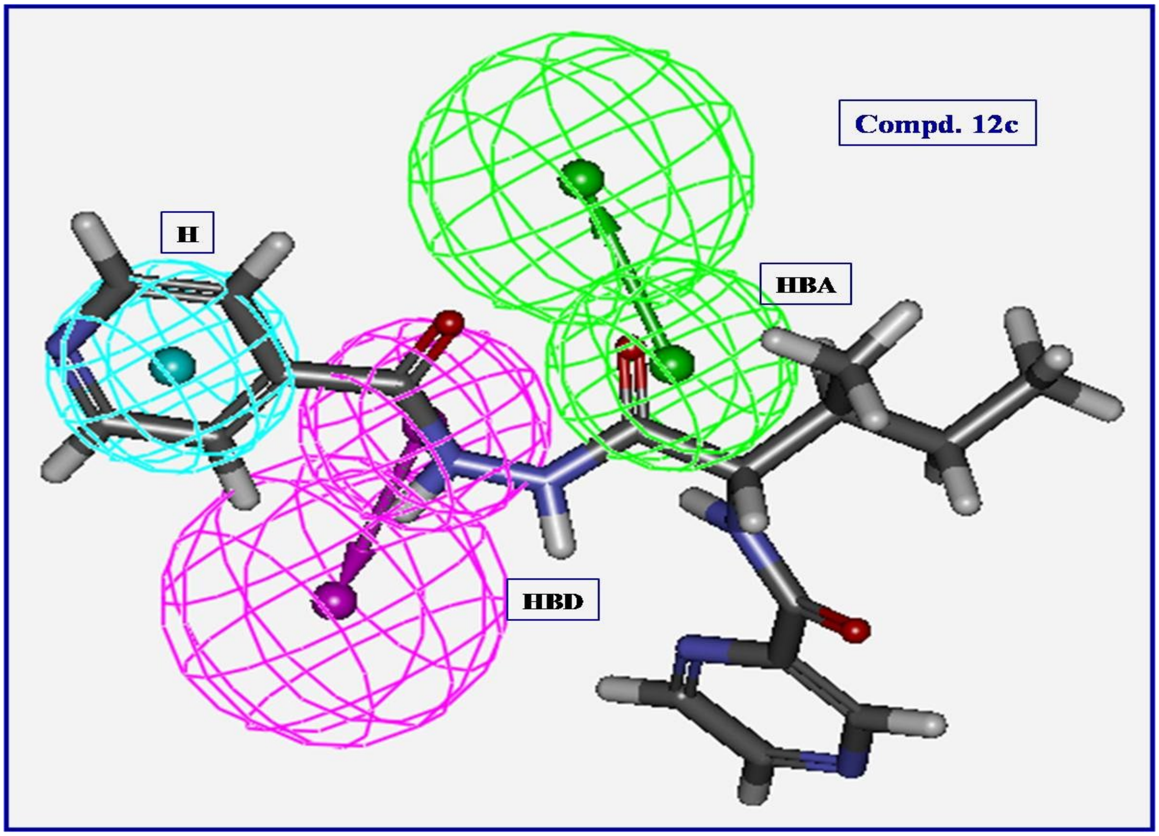
Compd. 12c
H
HBA
HBD

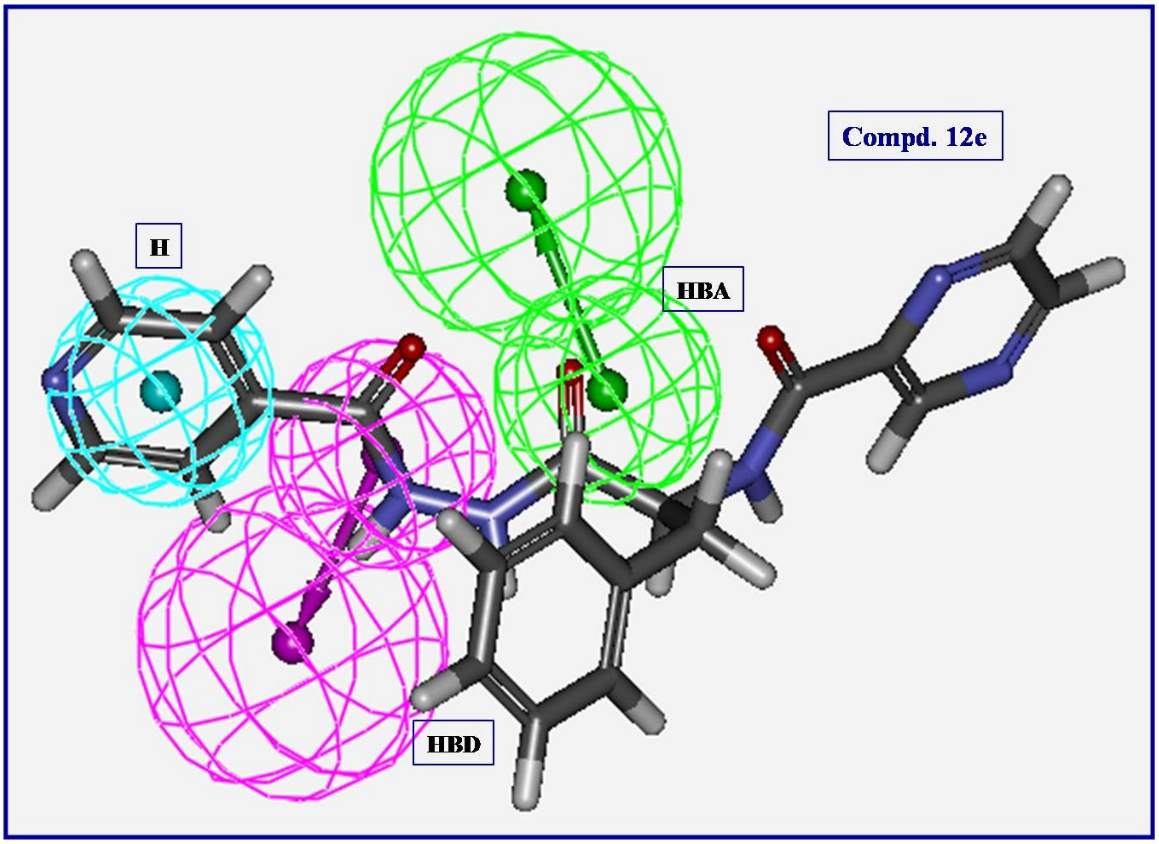
Compd. 12e
H
HBA
HBD

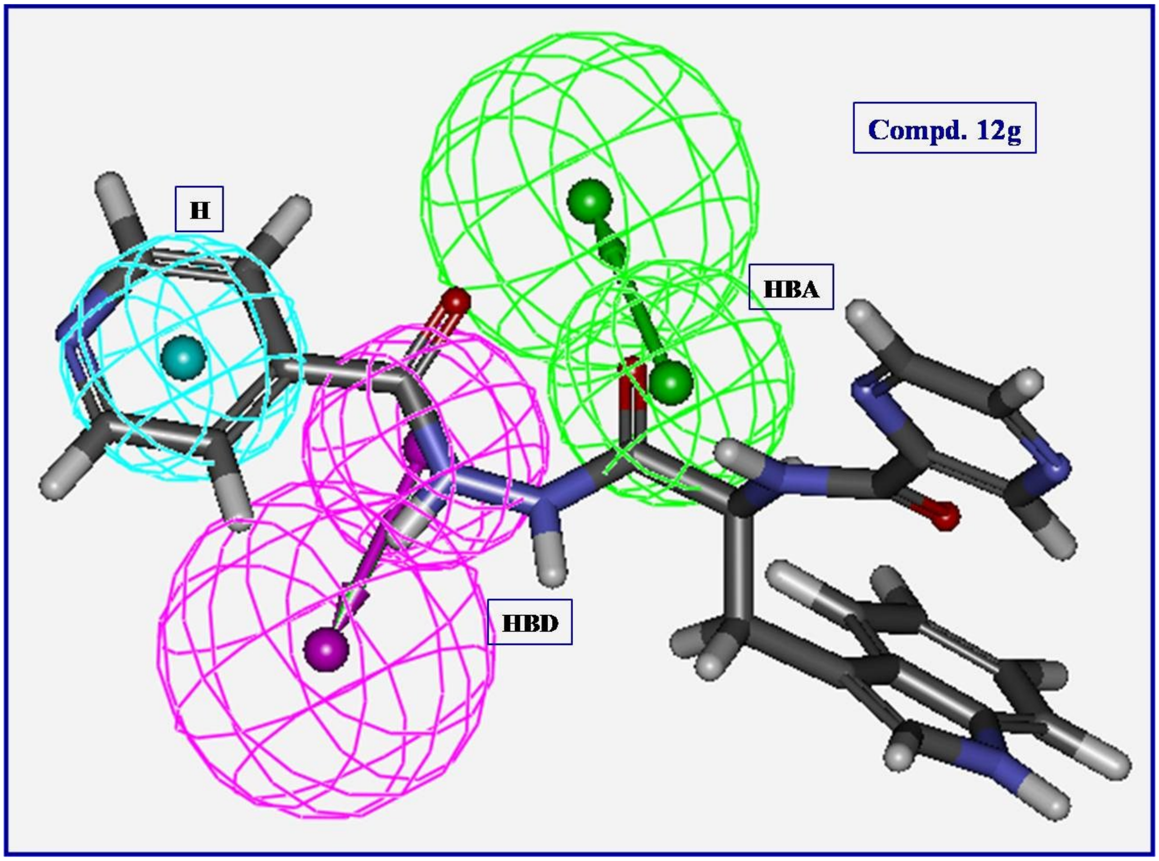
Compd. 12g
H
HBA
HBD

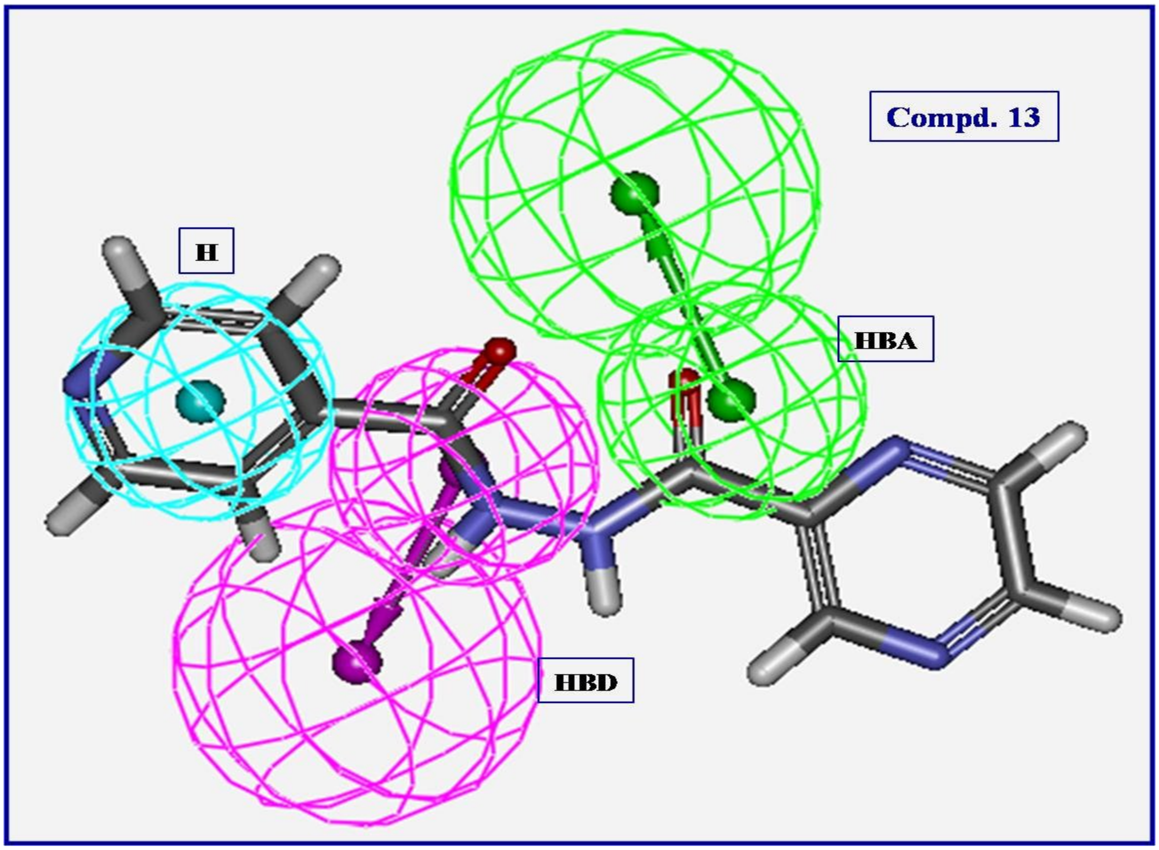
Compd. 13
H
HBA
HBD

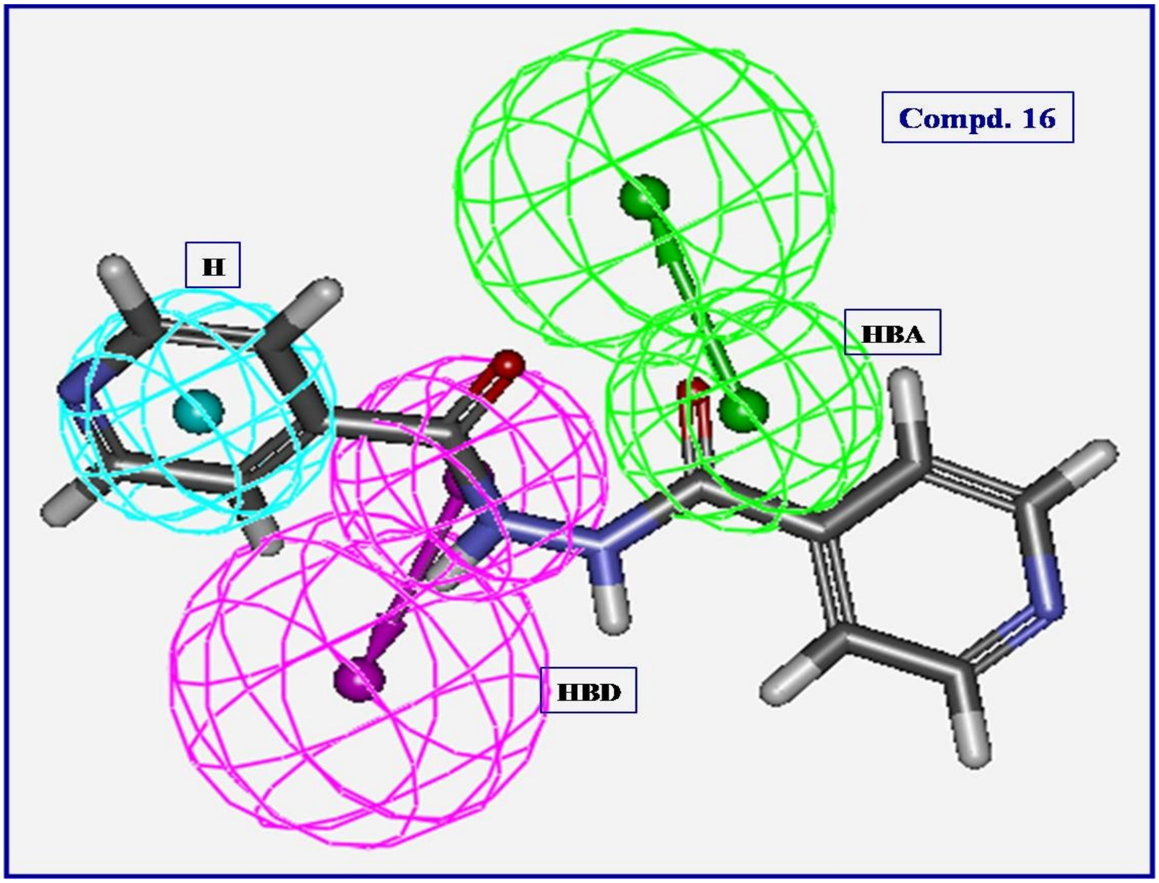

Fig. S6. 3D-pharmacophore mapped on the synthesized bio-active compounds against *Mycobacterium tuberculosis*.

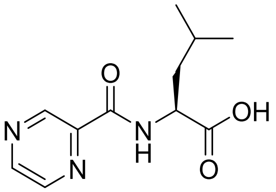

10a

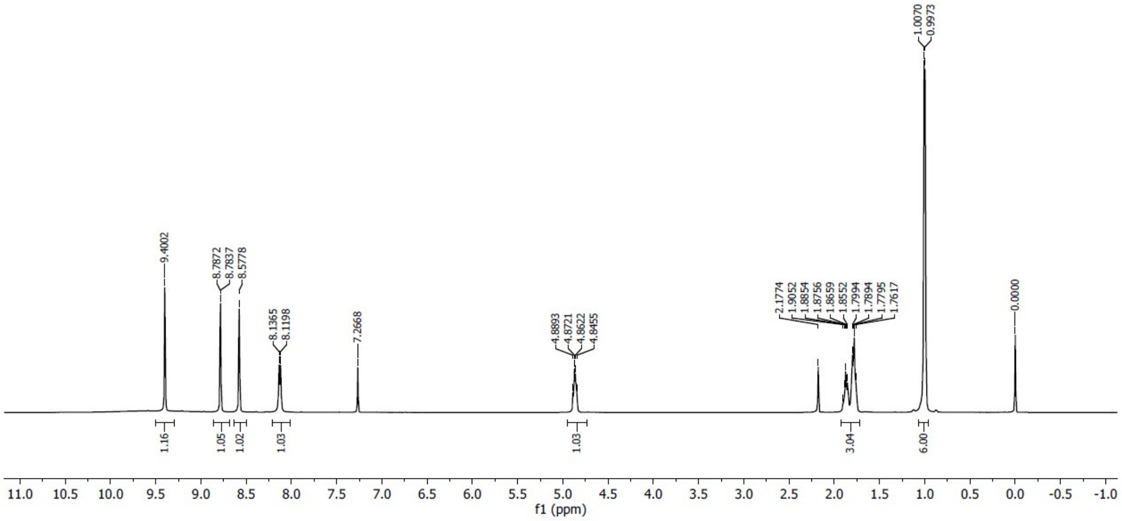
9.4002
8.7872
8.7837
8.5778
8.1365
8.1198
7.2668
4.8893
4.8721
4.8622
4.8455
2.1774
1.9052
1.8854
1.8756
1.8659
1.8552
1.7994
1.7894
1.7795
1.7617
1.0070
0.9973
0.0000
1.16
1.05
1.02
1.03
1.03
3.04
6.00
f1 (ppm)

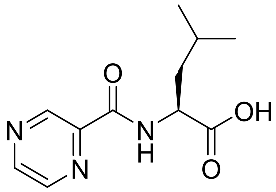

10a

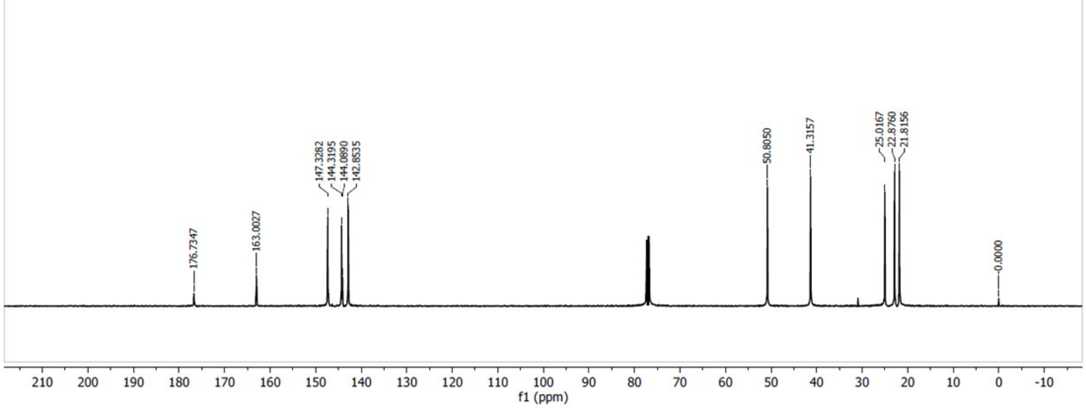
176.7347
163.0027
147.3282
144.3195
144.0890
142.8535
50.8050
41.3157
25.0167
22.8760
21.8156
0.0000
f1 (ppm)

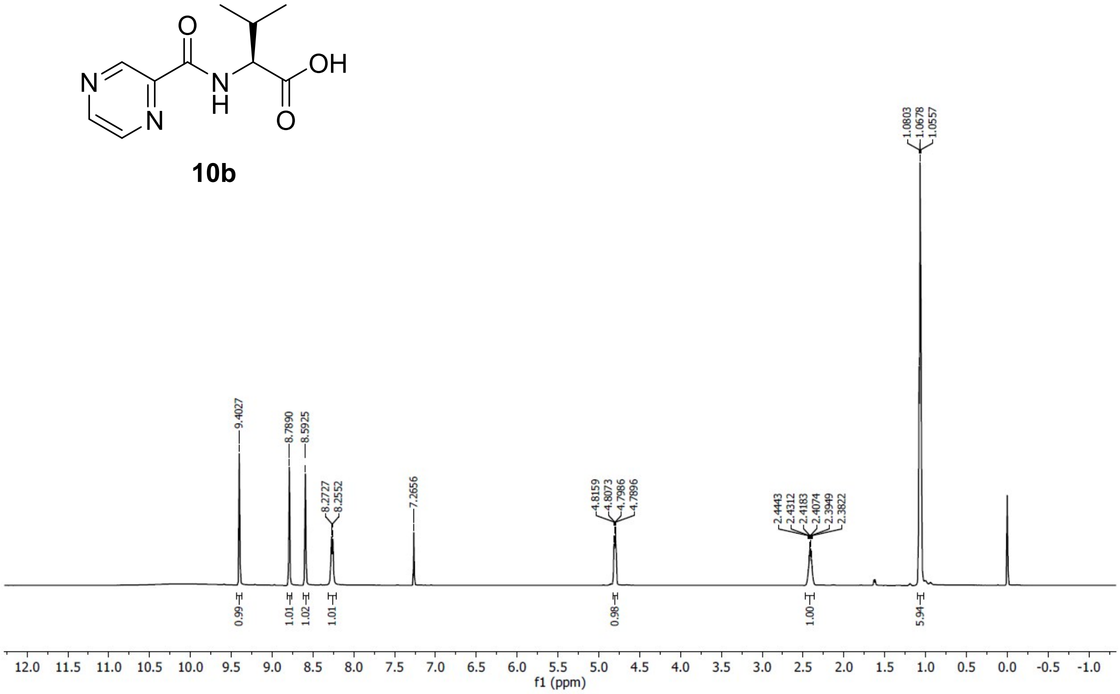
10b
9.4027
8.7890
8.5925
8.2727
8.2552
7.2656
4.8159
4.8073
4.7986
4.7896
2.4443
2.4312
2.4183
2.4074
2.3949
2.3822
1.0803
1.0678
1.0557
0.99
1.01
1.02
1.01
0.98
1.00
5.94
f1 (ppm)

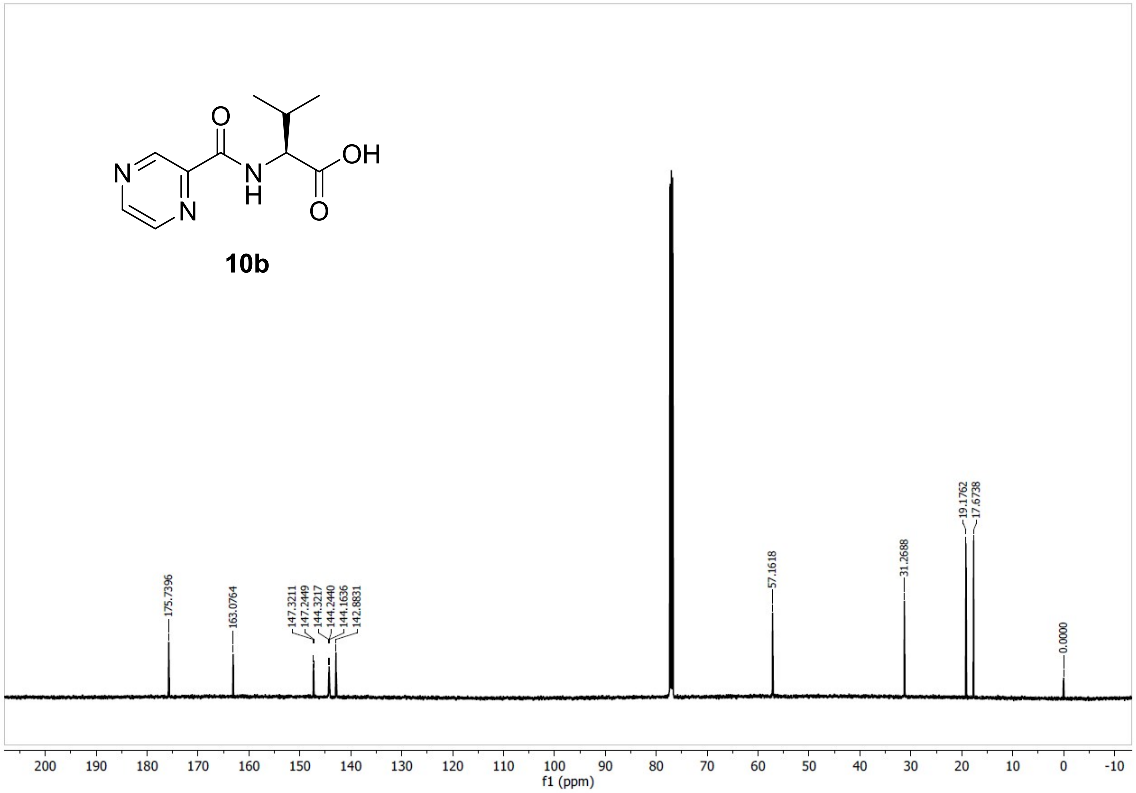
10b
175.7396
163.0764
147.3211
147.2449
144.3217
144.2440
144.1636
142.8831
57.1618
31.2688
19.1762
17.6738
0.0000
f1 (ppm)

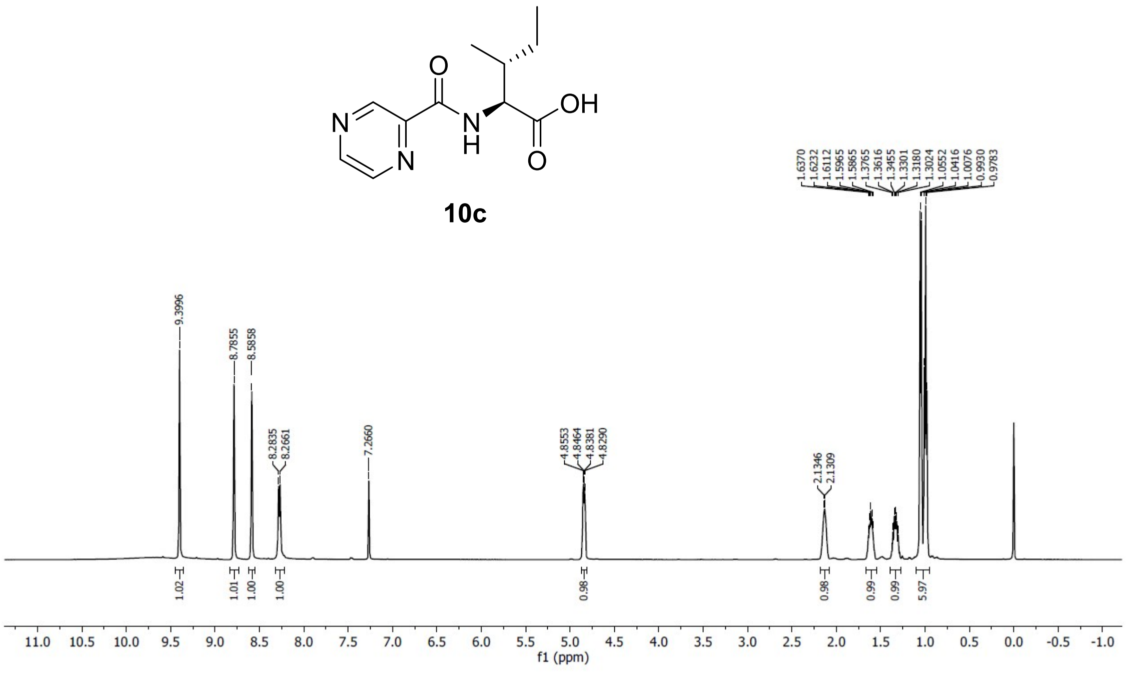
10c
9.3996
8.7855
8.5858
8.2835
8.2661
7.2660
4.8553
4.8464
4.8381
4.8290
2.1346
2.1309
1.6370
1.6232
1.6112
1.5965
1.5865
1.3765
1.3616
1.3455
1.3301
1.3180
1.3024
1.0552
1.0416
1.0076
0.9930
0.9783
1.02
1.01
1.00
1.00
0.98
0.98
0.99
0.99
5.97
f1 (ppm)

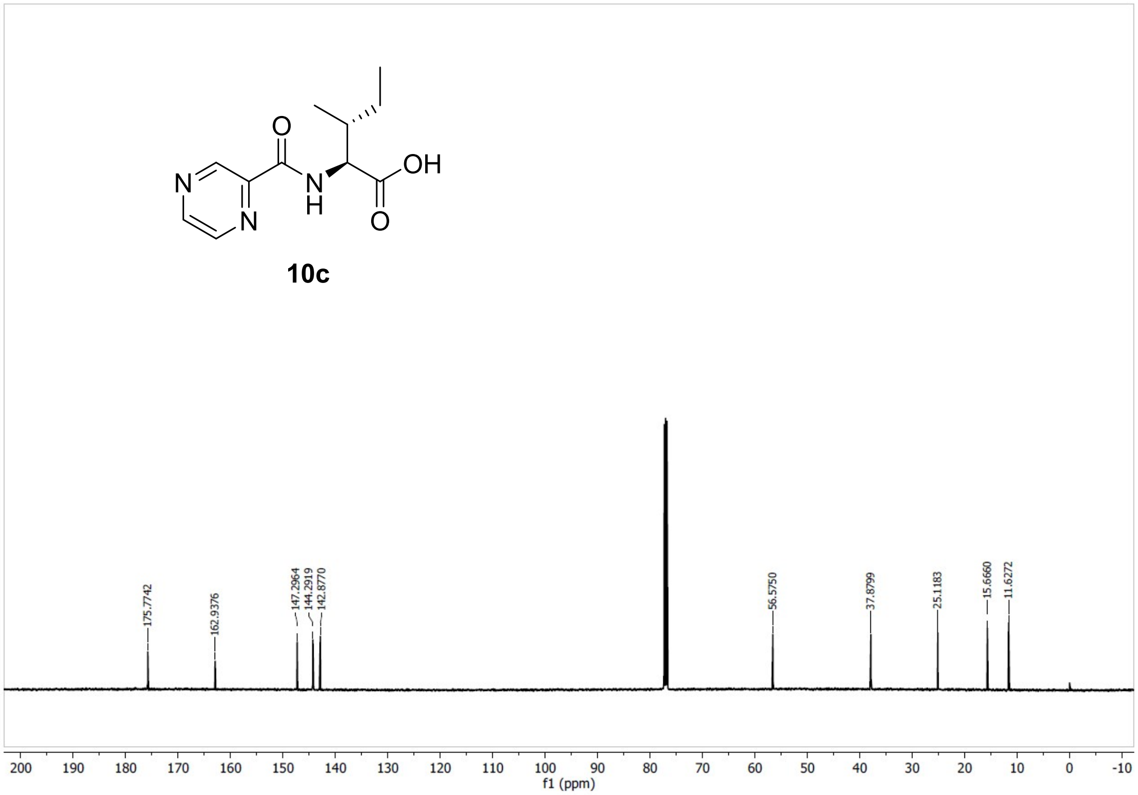
10c
175.7742
162.9376
147.2964
144.2919
142.8770
56.5750
37.8799
25.1183
15.6660
11.6272
f1 (ppm)

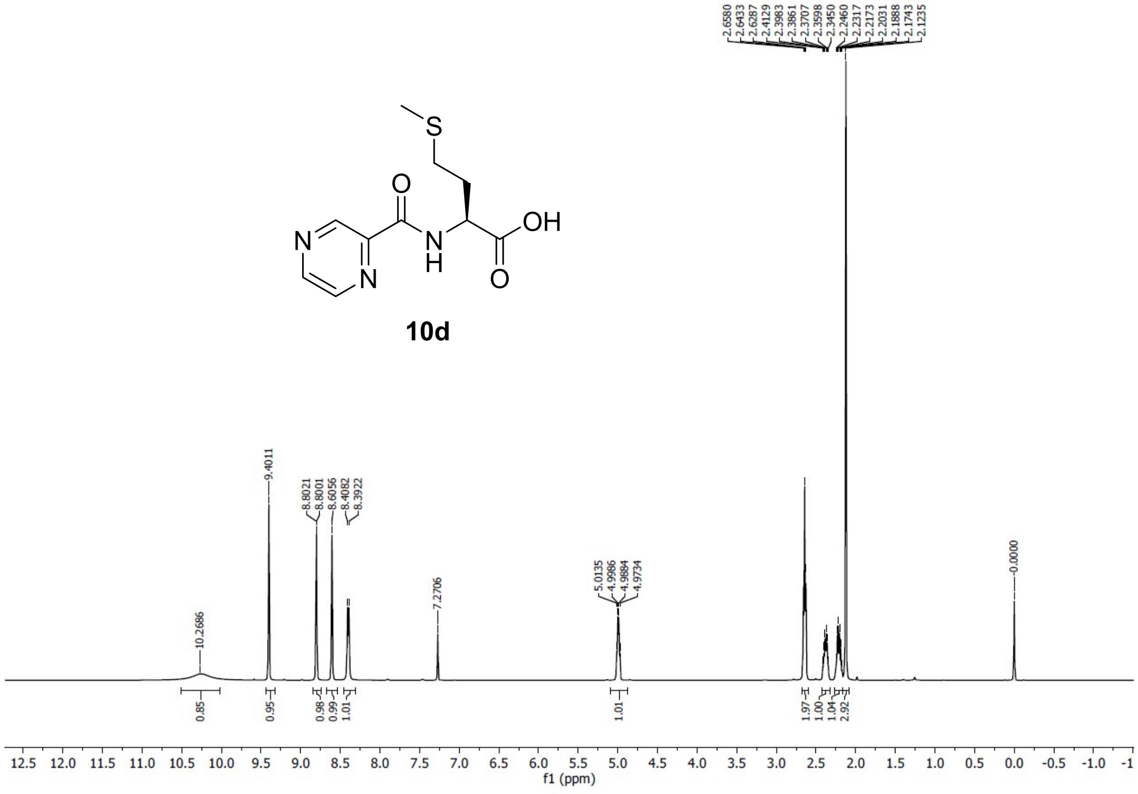

10d
2.6580
2.6433
2.6287
2.4129
2.3983
2.3861
2.3707
2.3598
2.3450
2.2460
2.2317
2.2173
2.2031
2.1888
2.1743
2.1235
10.2686
9.4011
8.8021
8.8001
8.6056
8.4082
8.3922
7.2706
5.0135
4.9986
4.9884
4.9734
0.0000
0.85
0.95
0.98
0.99
1.01
1.01
1.97
1.00
1.04
2.92
f1 (ppm)

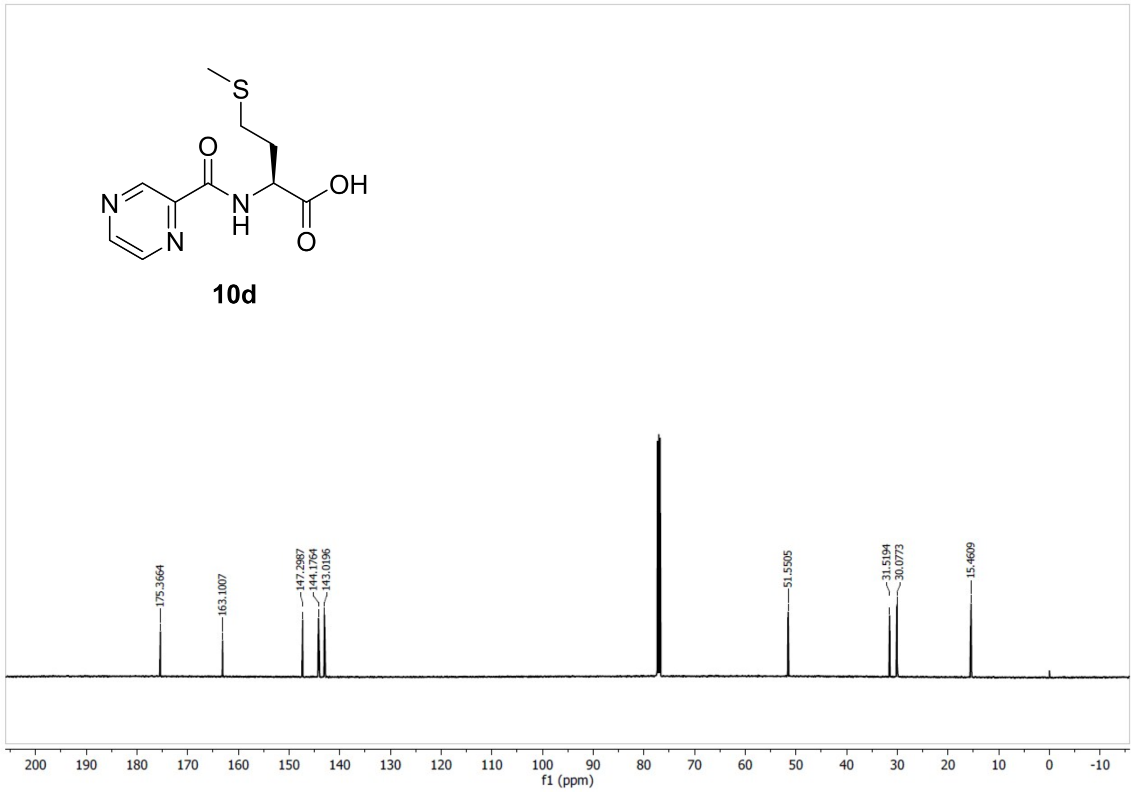

10d
175.3664
163.1007
147.2987
144.1764
143.0196
51.5505
31.5194
30.0773
15.4609
f1 (ppm)

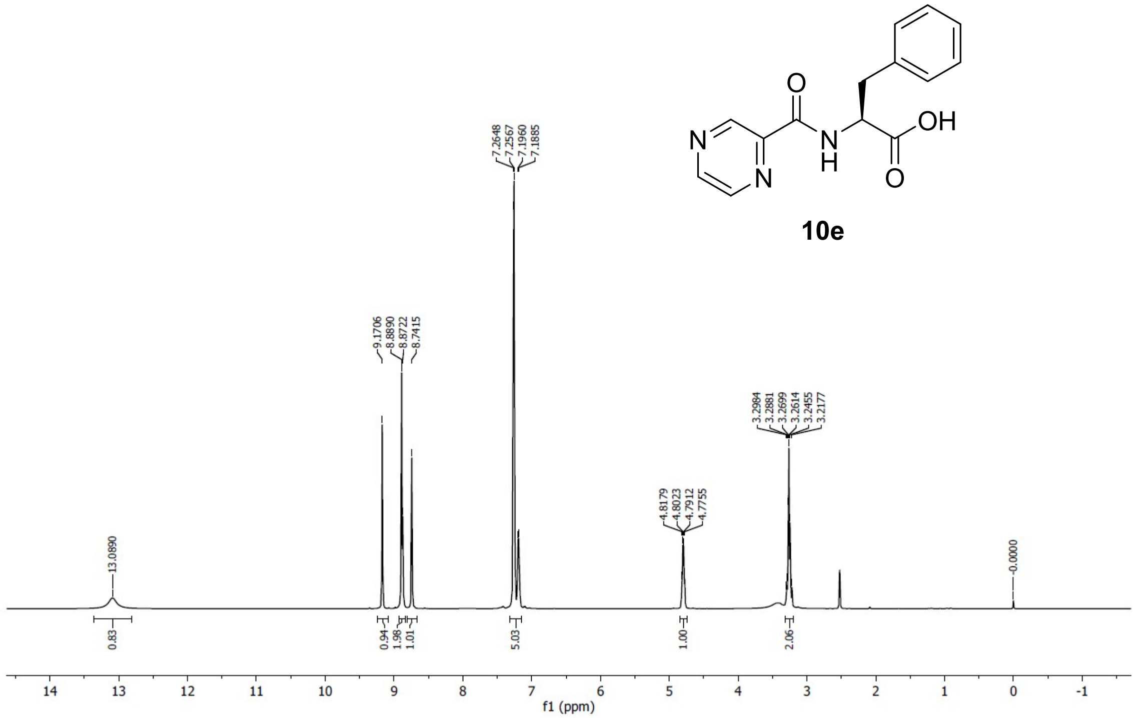
10e
N
N
N
H
O
OH
O
13.0890
9.1706
8.8890
8.8722
8.7415
7.2648
7.2567
7.1960
7.1885
4.8179
4.8023
4.7912
4.7755
3.2984
3.2881
3.2699
3.2614
3.2455
3.2177
0.0000
0.83
0.94
1.98
1.01
5.03
1.00
2.06
f1 (ppm)

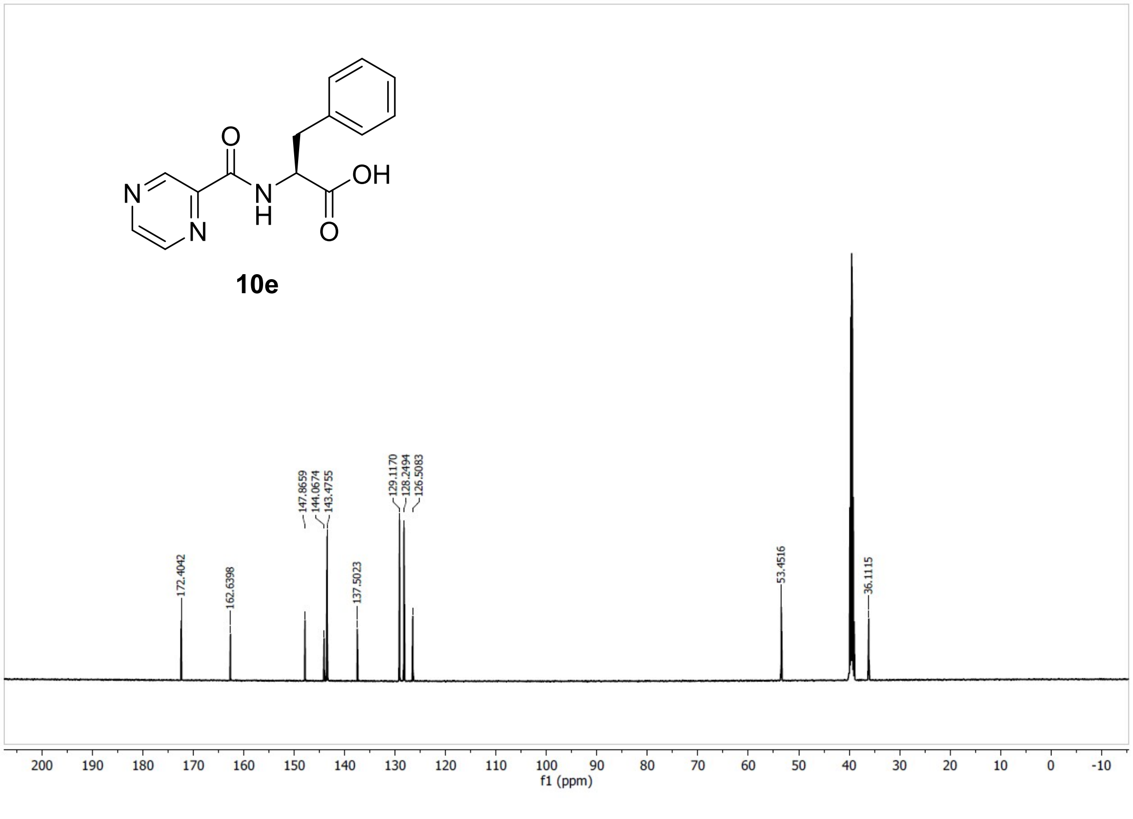
10e
N
N
N
H
O
OH
O
172.4042
162.6398
147.8659
144.0674
143.4755
137.5023
129.1170
128.2494
126.5083
53.4516
36.1115
f1 (ppm)

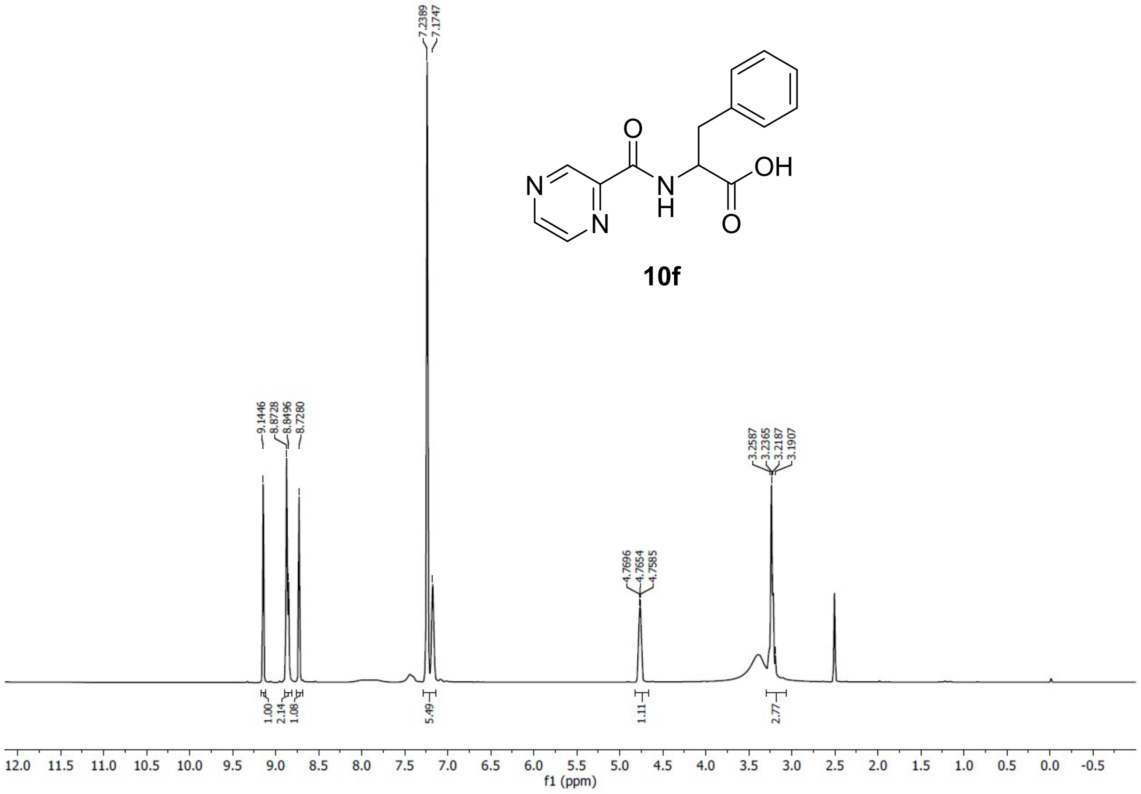
10f
9.1446
8.8728
8.8496
8.7280
7.2389
7.1747
4.7696
4.7654
4.7585
3.2587
3.2365
3.2187
3.1907
1.00
2.14
1.08
5.49
1.11
2.77
f1 (ppm)

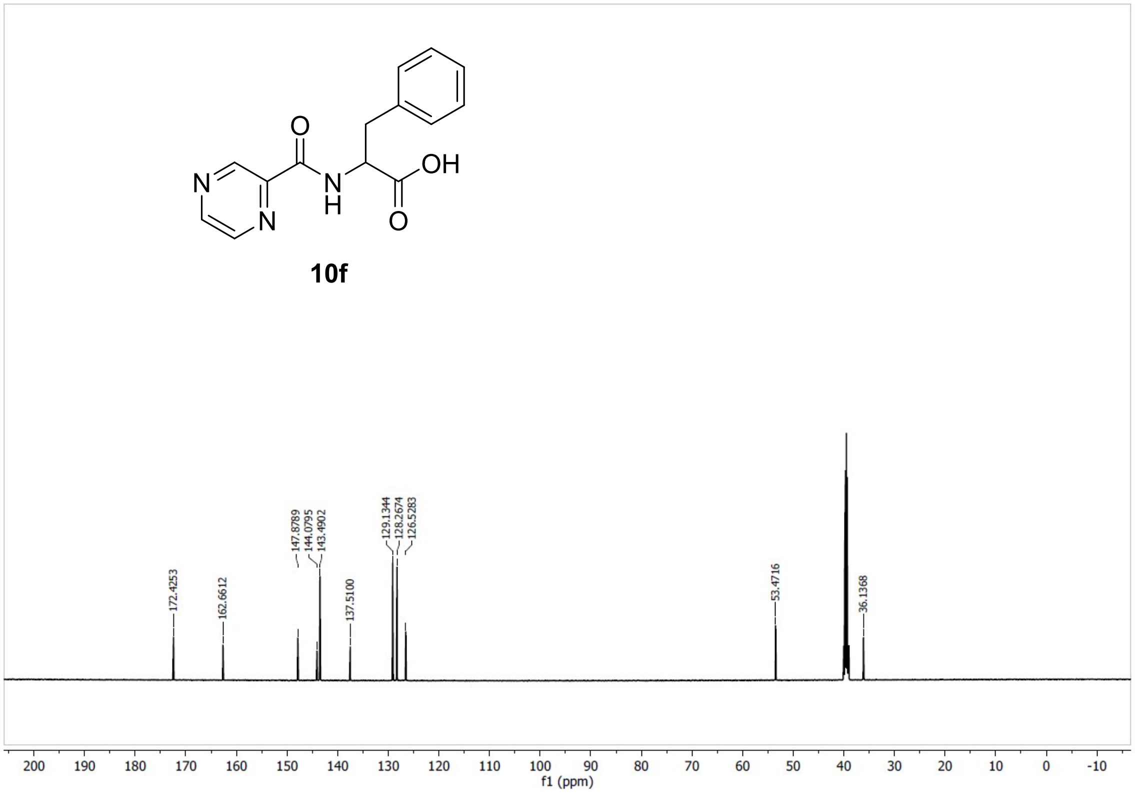
10f
172.4253
162.6612
147.8789
144.0795
143.4902
137.5100
129.1344
128.2674
126.5283
53.4716
36.1368
f1 (ppm)

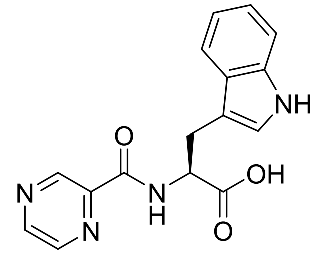
NH
O
OH
N
N
H
N
O

10g

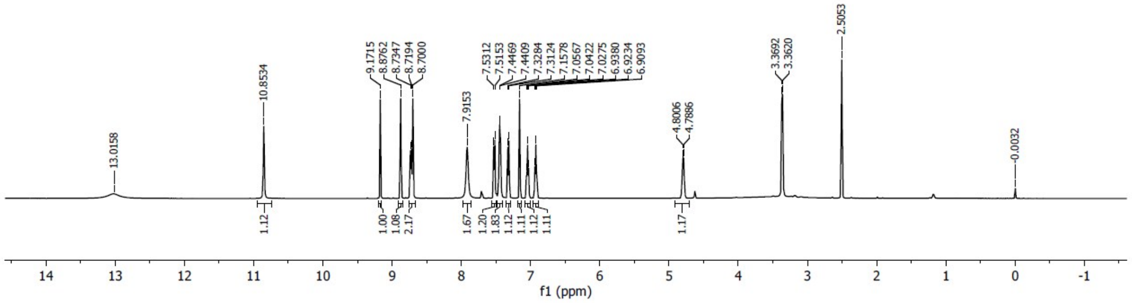
13.0158
10.8534
9.1715
8.8762
8.7347
8.7194
8.7000
7.9153
7.5312
7.5153
7.4469
7.4409
7.3284
7.3124
7.1578
7.0567
7.0422
7.0275
6.9380
6.9234
6.9093
4.8006
4.7886
3.3692
3.3620
2.5053
-0.0032
1.12
1.00
1.08
2.17
1.67
1.20
1.83
1.12
1.11
1.12
1.11
1.17
f1 (ppm)

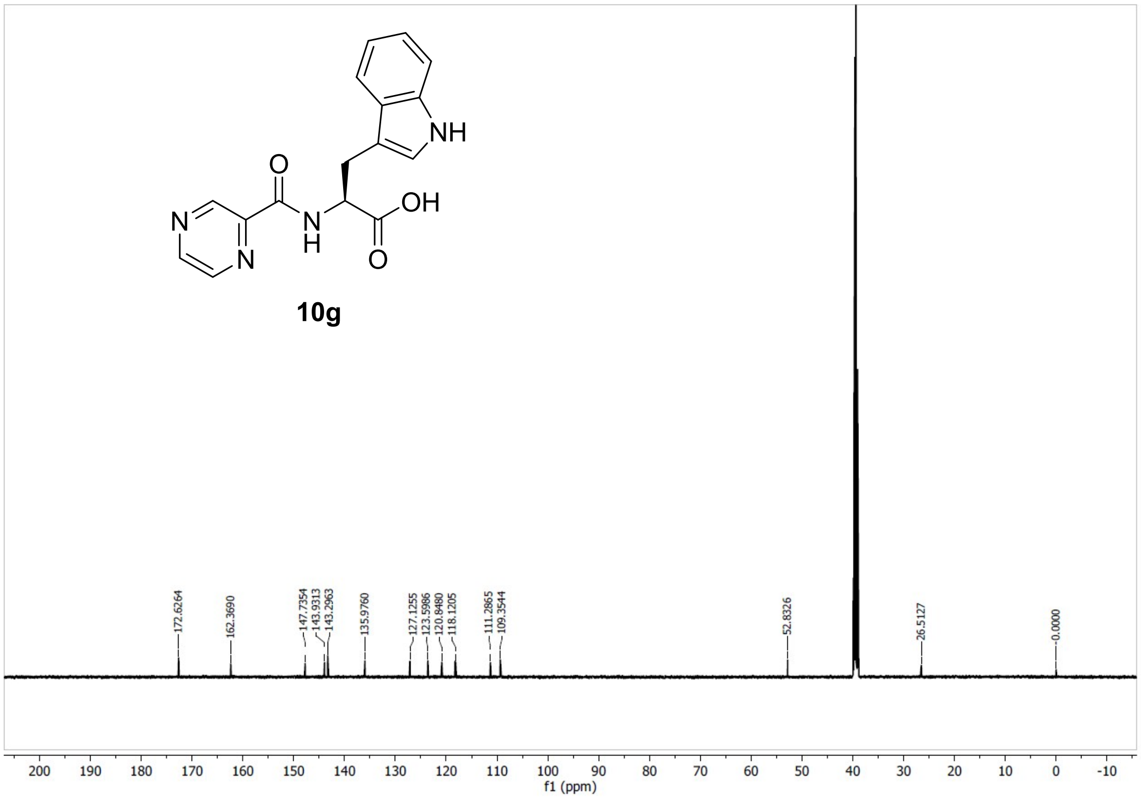
172.6264
162.3690
147.7354
143.9313
143.2963
135.9760
127.1255
123.5986
120.8480
118.1205
111.2865
109.3544
52.8326
26.5127
0.0000
f1 (ppm)
NH
O
OH
N
N
H
N
O
10g

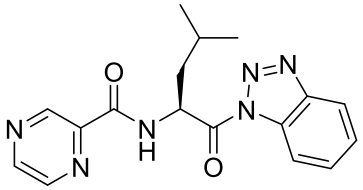
O
N
N
N
H
O
N
N=N

11a

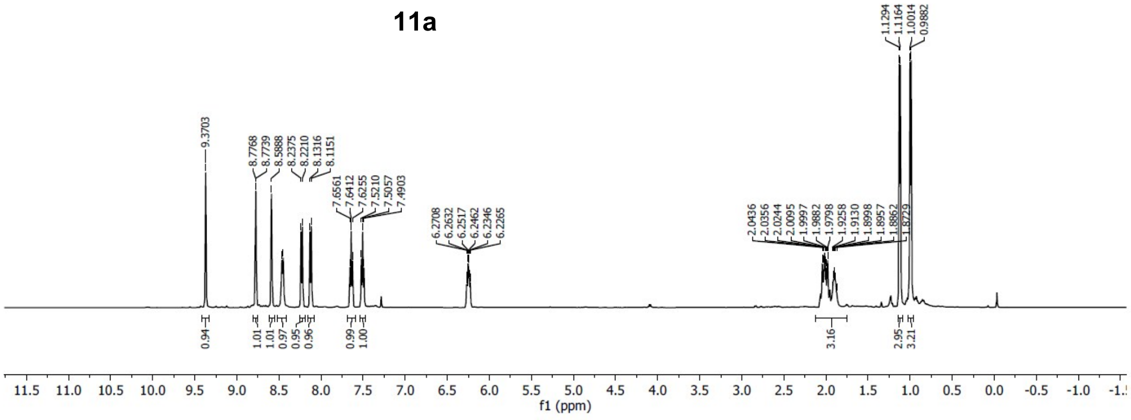
9.3703
8.7768
8.7739
8.5888
8.2375
8.2210
8.1316
8.1151
7.6561
7.6412
7.6255
7.5210
7.5057
7.4903
6.2708
6.2632
6.2517
6.2462
6.2346
6.2265
2.0436
2.0356
2.0244
2.0095
1.9997
1.9882
1.9798
1.9258
1.9130
1.8998
1.8957
1.8862
1.8729
1.1294
1.1164
1.0014
0.9882
0.94
1.01
1.01
0.97
0.95
0.96
0.99
1.00
3.16
2.95
3.21
f1 (ppm)

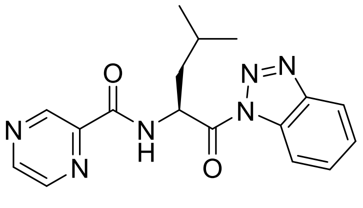
O
N
N
N
H
O
N
N=N

11a

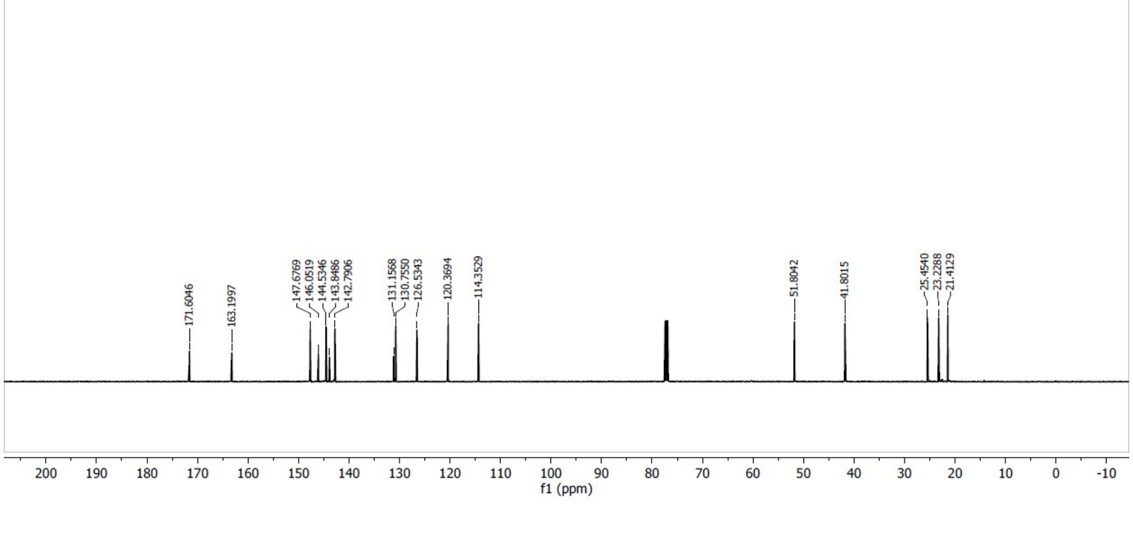
171.6046
163.1997
147.6769
146.0519
144.5346
143.9486
142.7906
131.1568
130.7550
126.5343
120.3694
114.3529
51.8042
41.8015
25.4540
23.2288
21.4129
f1 (ppm)

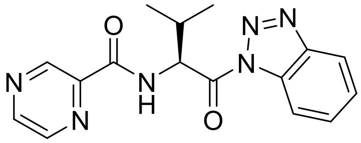

11b

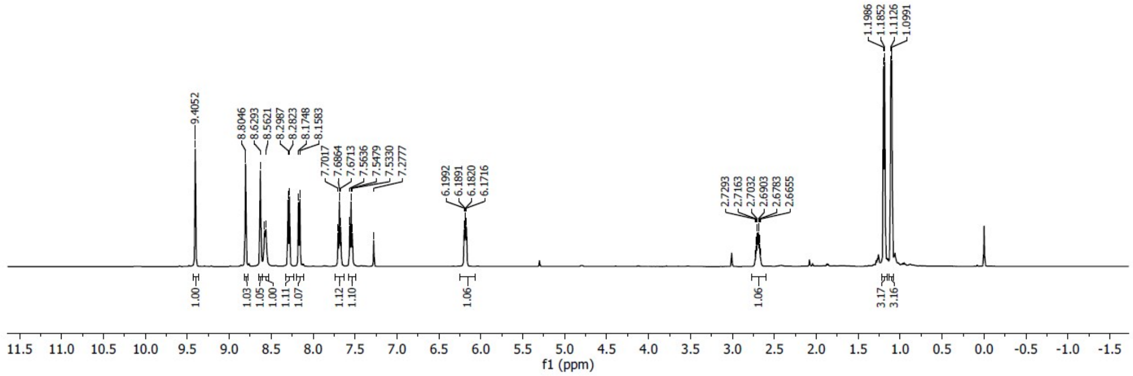
9.4052
8.8046 8.6293 8.5621 8.2987 8.2823 8.1748 8.1583
7.7017 7.6864 7.6713 7.5636 7.5479 7.5330 7.2777
6.1992 6.1891 6.1820 6.1716
2.7293 2.7163 2.7032 2.6903 2.6783 2.6655
1.1986 1.1852 1.1126 1.0991
1.00 1.03 1.05 1.00 1.11 1.07 1.12 1.10 1.06 1.06 3.17 3.16
f1 (ppm)

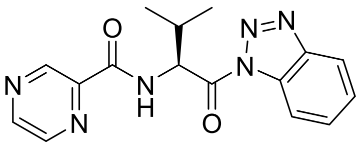

11b

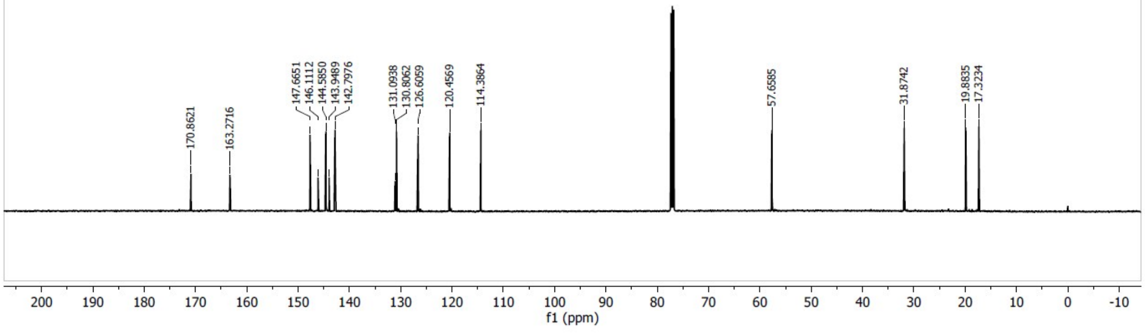
170.8621
163.2716
147.6651 146.1112 144.5850 143.9489 142.7976
131.0938 130.8062 126.6059
120.4569
114.3864
57.6585
31.8742
19.8835 17.3234
f1 (ppm)

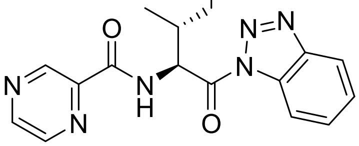

11c

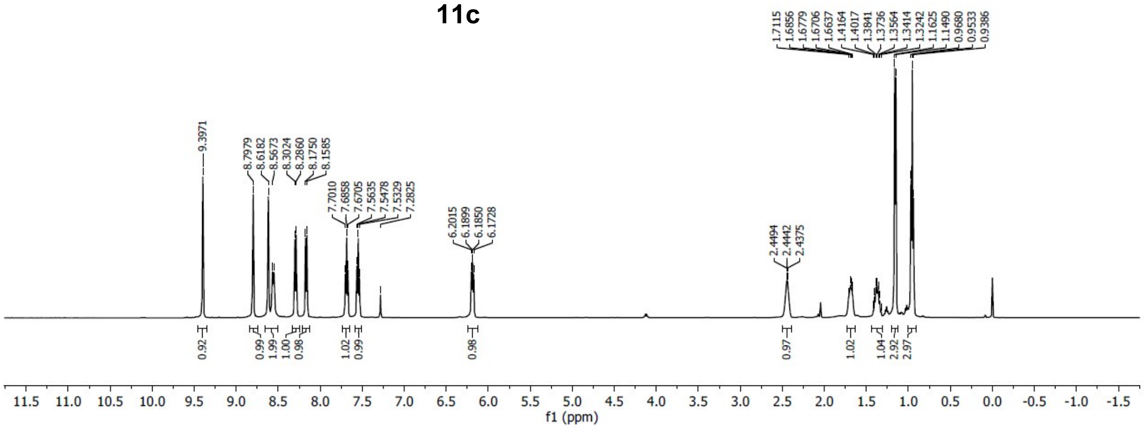

9.3971
8.7979
8.6182
8.5673
8.3024
8.2860
8.1750
8.1585
7.7010
7.6858
7.6705
7.5635
7.5478
7.5329
7.2825
6.2015
6.1899
6.1850
6.1728
2.4494
2.4442
2.4375
1.7115
1.6856
1.6779
1.6706
1.6637
1.4164
1.4017
1.3841
1.3736
1.3564
1.3414
1.3242
1.1625
1.1490
0.9680
0.9533
0.9386
0.92
0.99
1.99
1.00
0.98
1.02
0.99
0.98
0.97
1.02
1.04
2.92
2.97
11.5 11.0 10.5 10.0 9.5 9.0 8.5 8.0 7.5 7.0 6.5 6.0 5.5 5.0 4.5 4.0 3.5 3.0 2.5 2.0 1.5 1.0 0.5 0.0 -0.5 -1.0 -1.5
f1 (ppm)

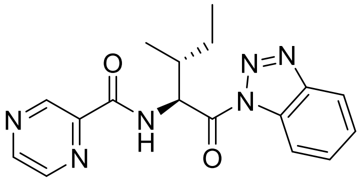

11c

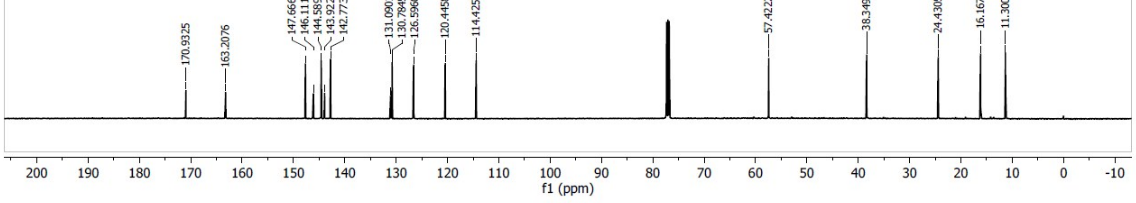

170.9325
163.2076
147.6662
146.1115
144.5894
143.9220
142.7731
131.0901
130.7845
126.5960
120.4458
114.4251
57.4222
38.3490
24.4305
16.1678
11.3009
200 190 180 170 160 150 140 130 120 110 100 90 80 70 60 50 40 30 20 10 0 -10
f1 (ppm)

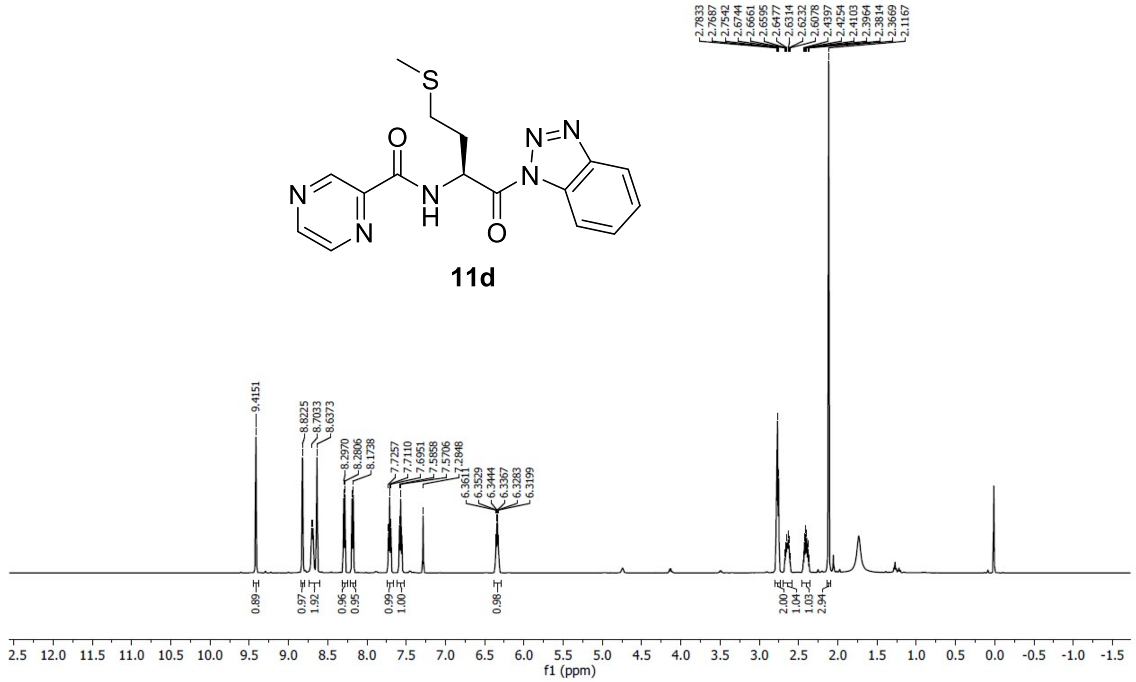
11d
f1 (ppm)

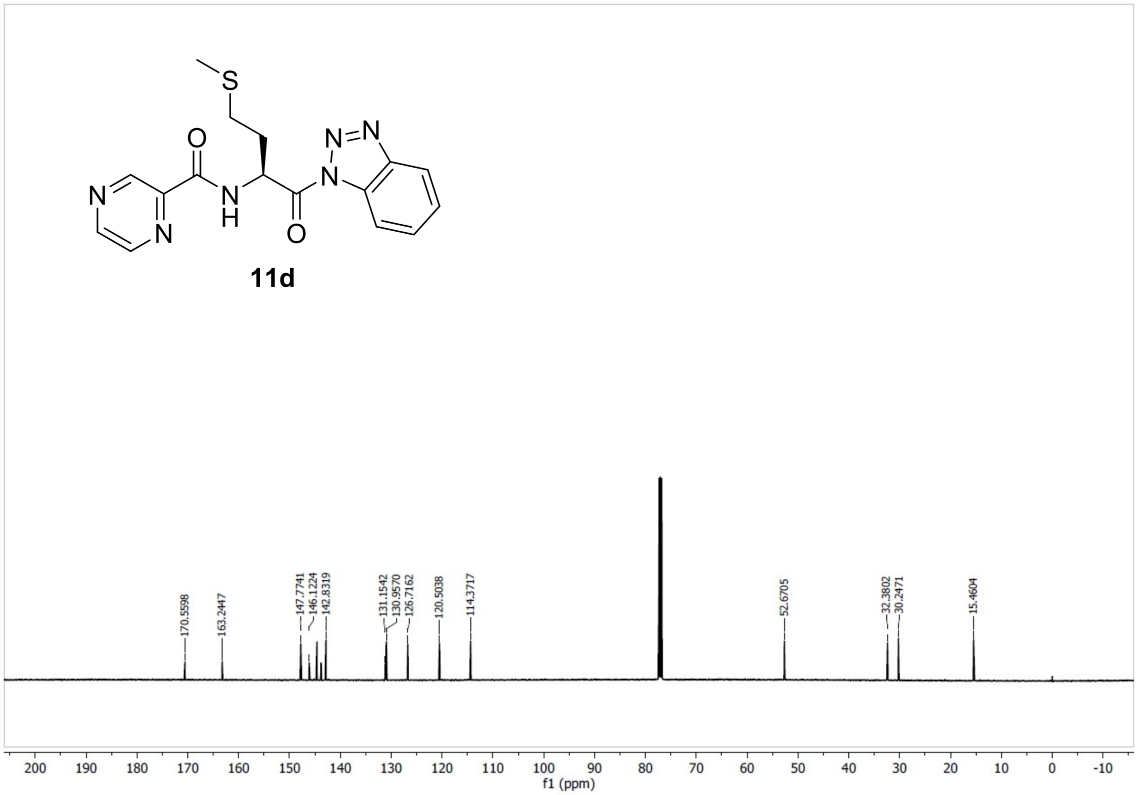
11d
f1 (ppm)

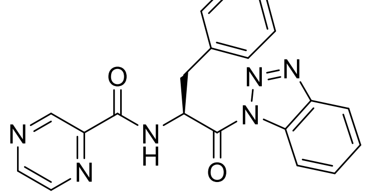

11e

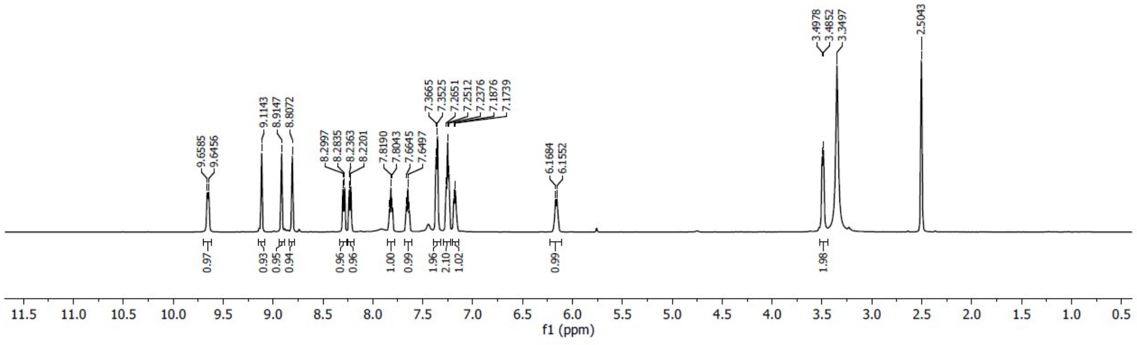

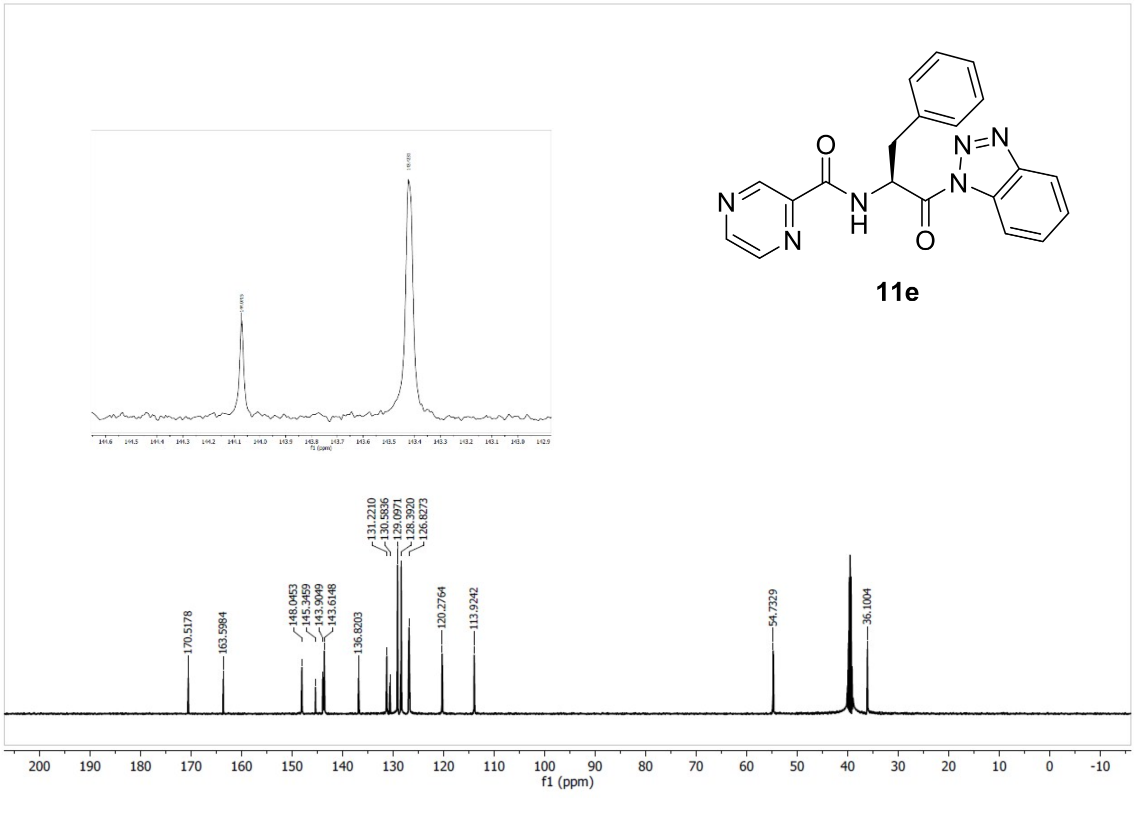

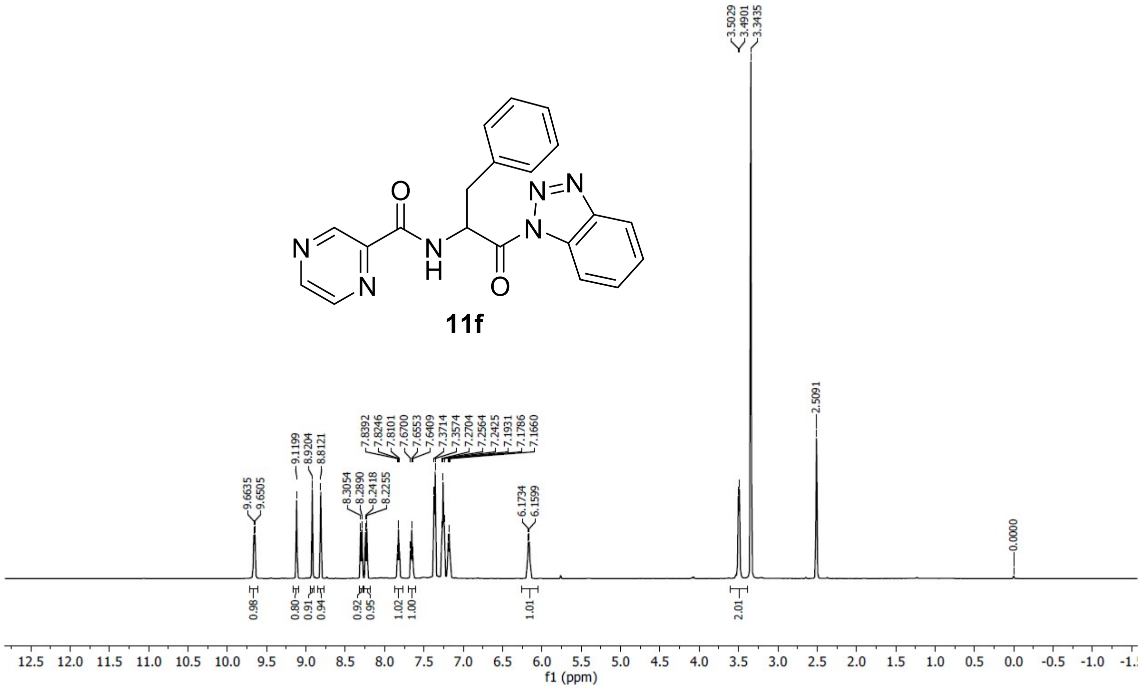
11f
f1 (ppm)

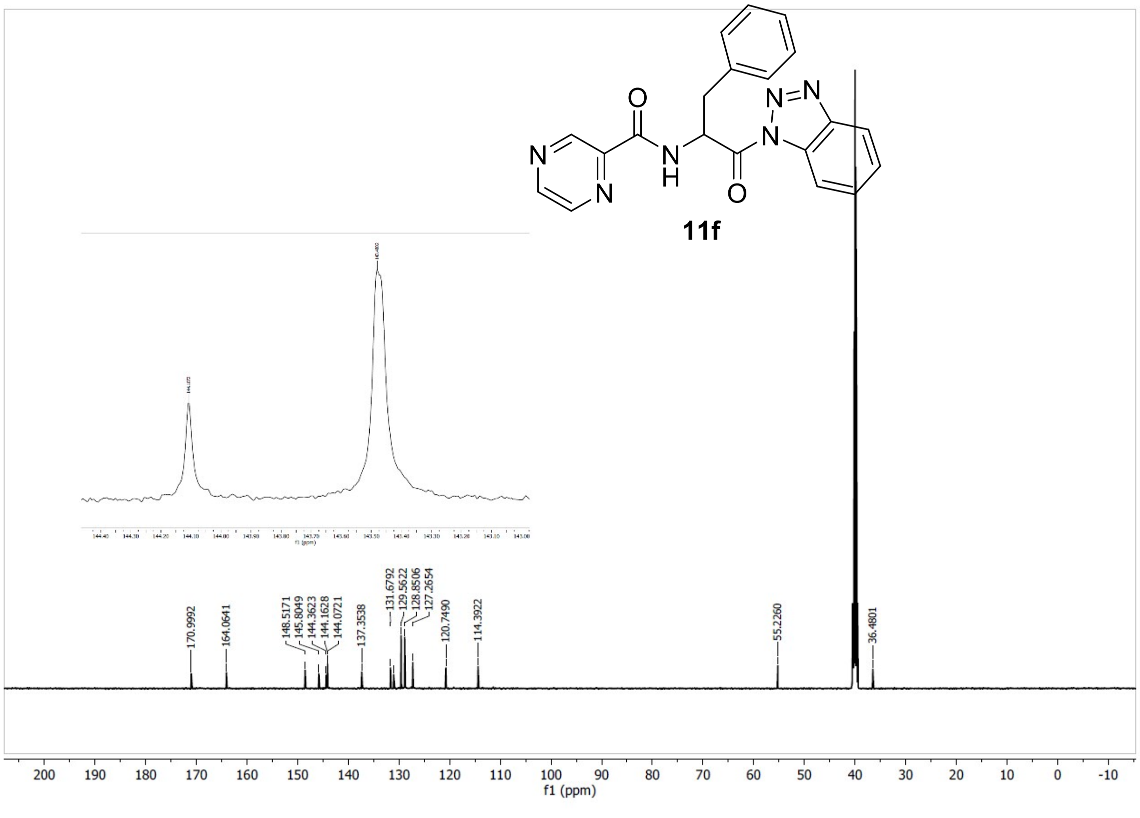
11f
f1 (ppm)

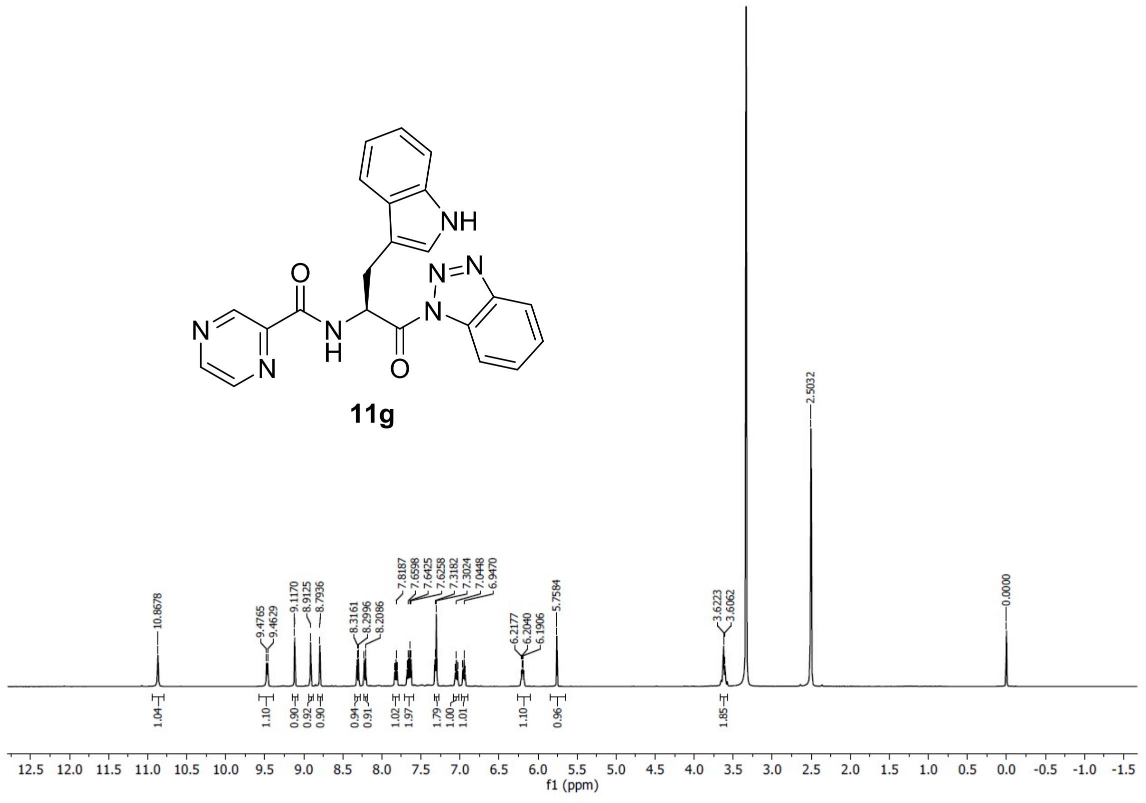

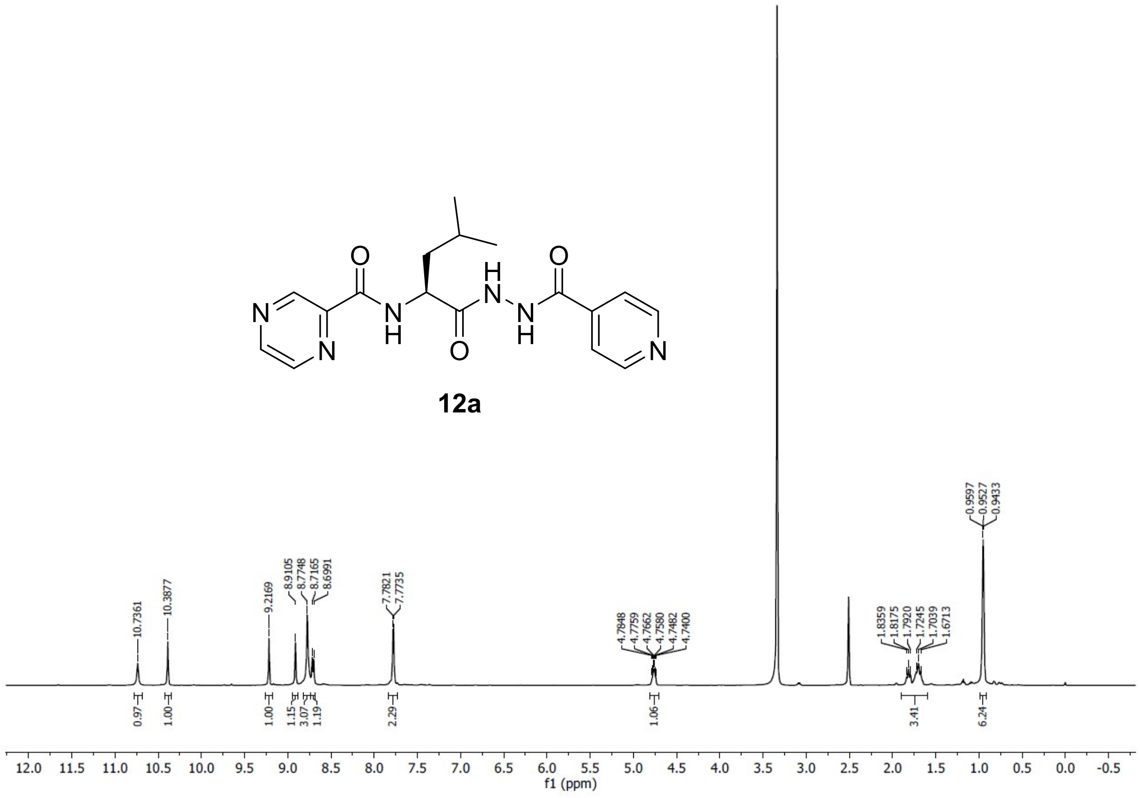

O
H
N
N
O
N
N
H
N
H
O
N
12a
10.7361
10.3877
9.2169
8.9105
8.7748
8.7165
8.6991
7.7821
7.7735
4.7848
4.7759
4.7662
4.7580
4.7482
4.7400
1.8359
1.8175
1.7920
1.7245
1.7039
1.6713
0.9597
0.9527
0.9433
0.97
1.00
1.00
1.15
3.07
1.19
2.29
1.06
3.41
6.24
12.0 11.5 11.0 10.5 10.0 9.5 9.0 8.5 8.0 7.5 7.0 6.5 6.0 5.5 5.0 4.5 4.0 3.5 3.0 2.5 2.0 1.5 1.0 0.5 0.0 -0.5
f1 (ppm)

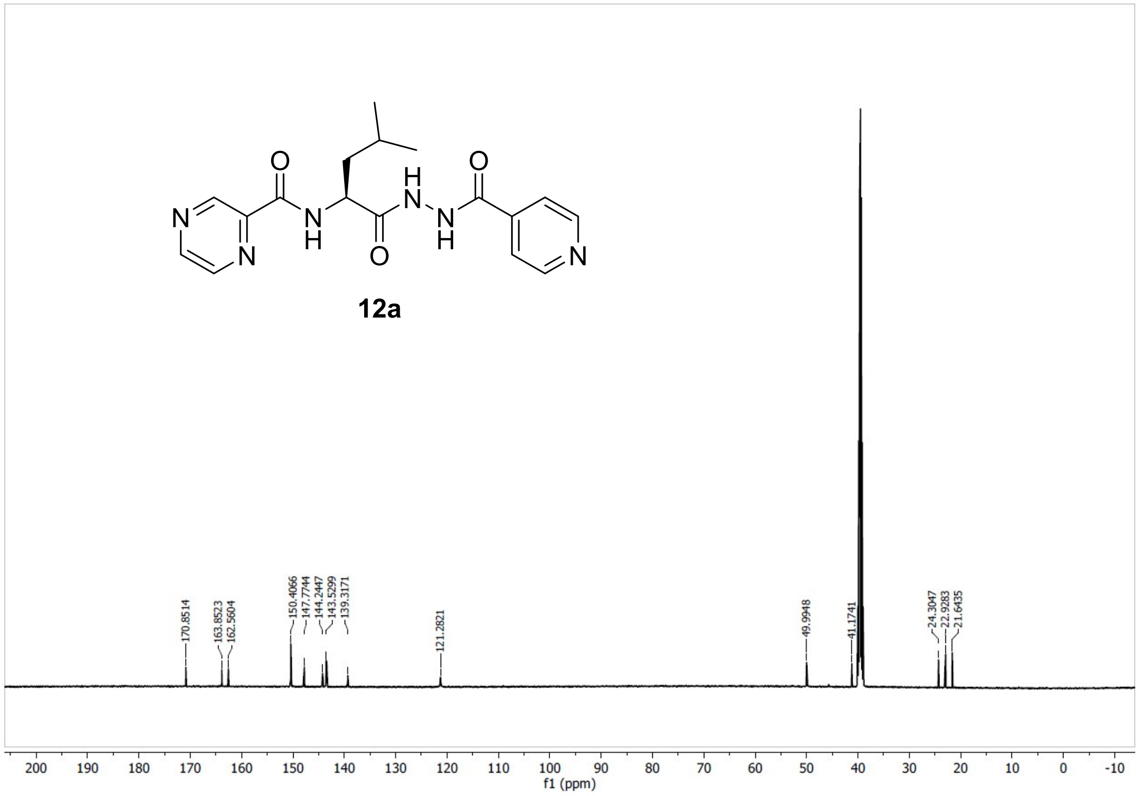

O
H
N
N
O
N
N
H
N
H
O
N
12a
170.8514
163.8523
162.5604
150.4066
147.7744
144.2447
143.5299
139.3171
121.2821
49.9948
41.1741
24.3047
22.9283
21.6435
200 190 180 170 160 150 140 130 120 110 100 90 80 70 60 50 40 30 20 10 0 -10
f1 (ppm)

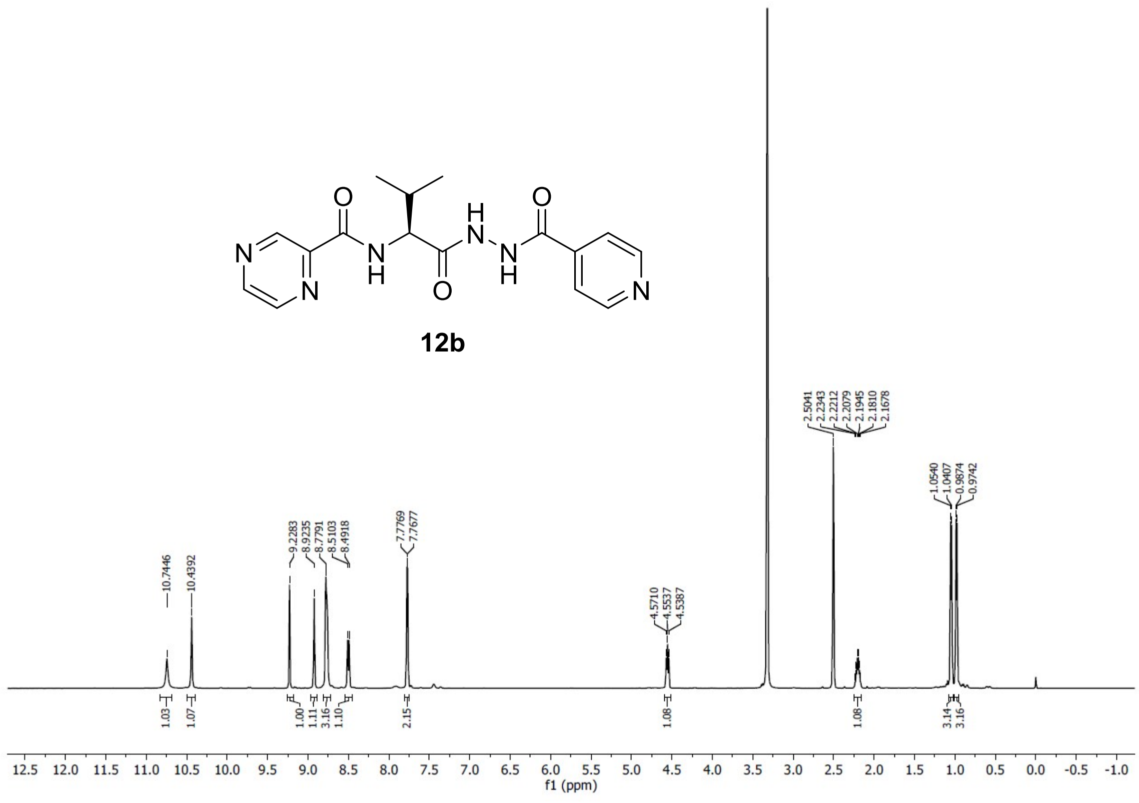

12b
f1 (ppm)

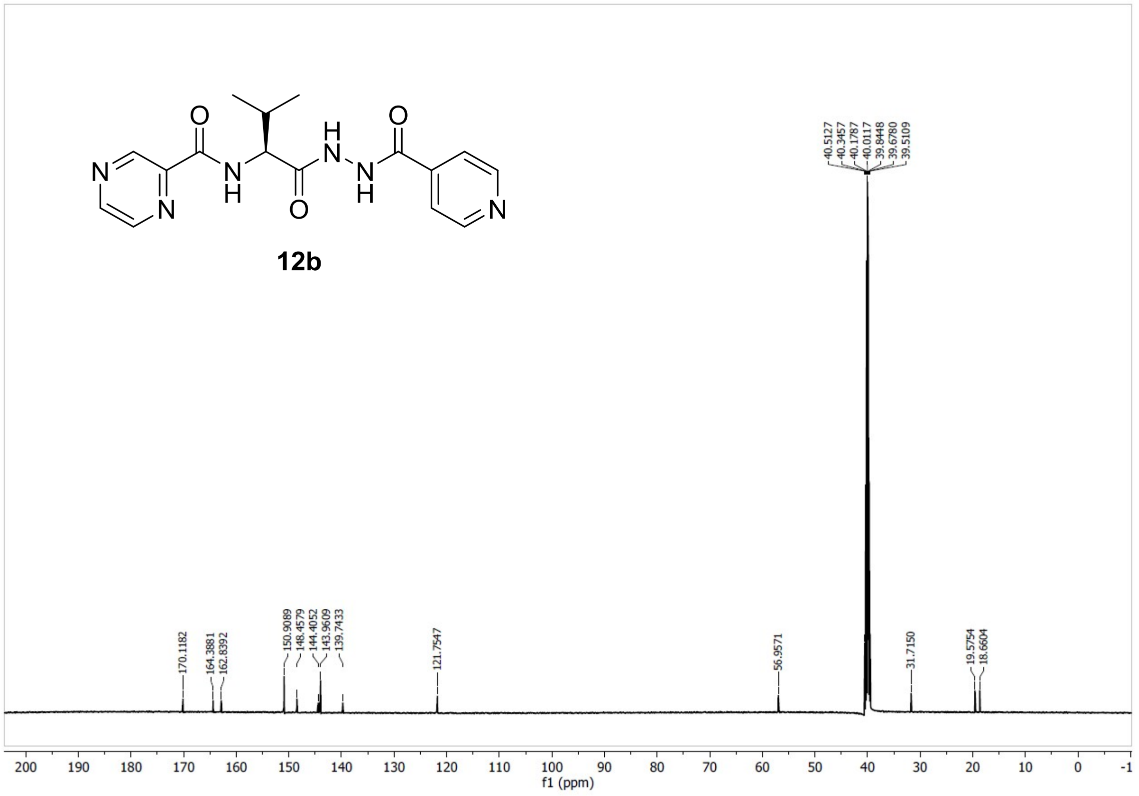

12b
f1 (ppm)

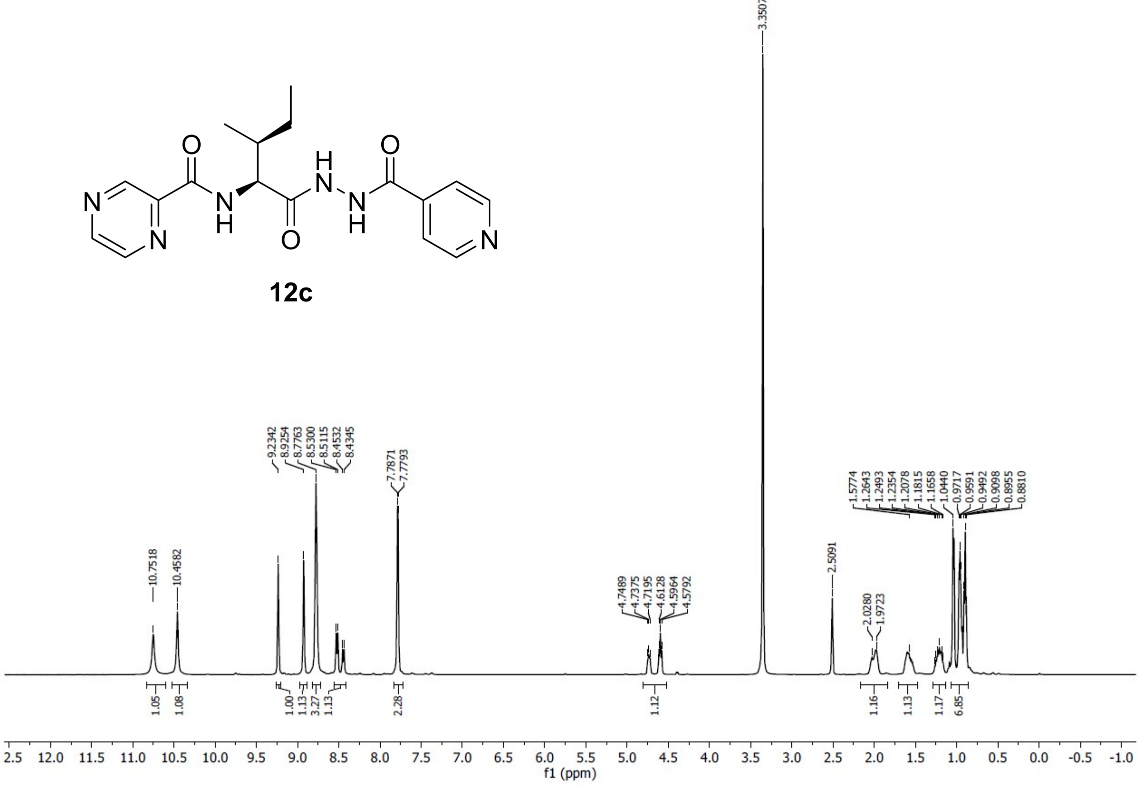

12c

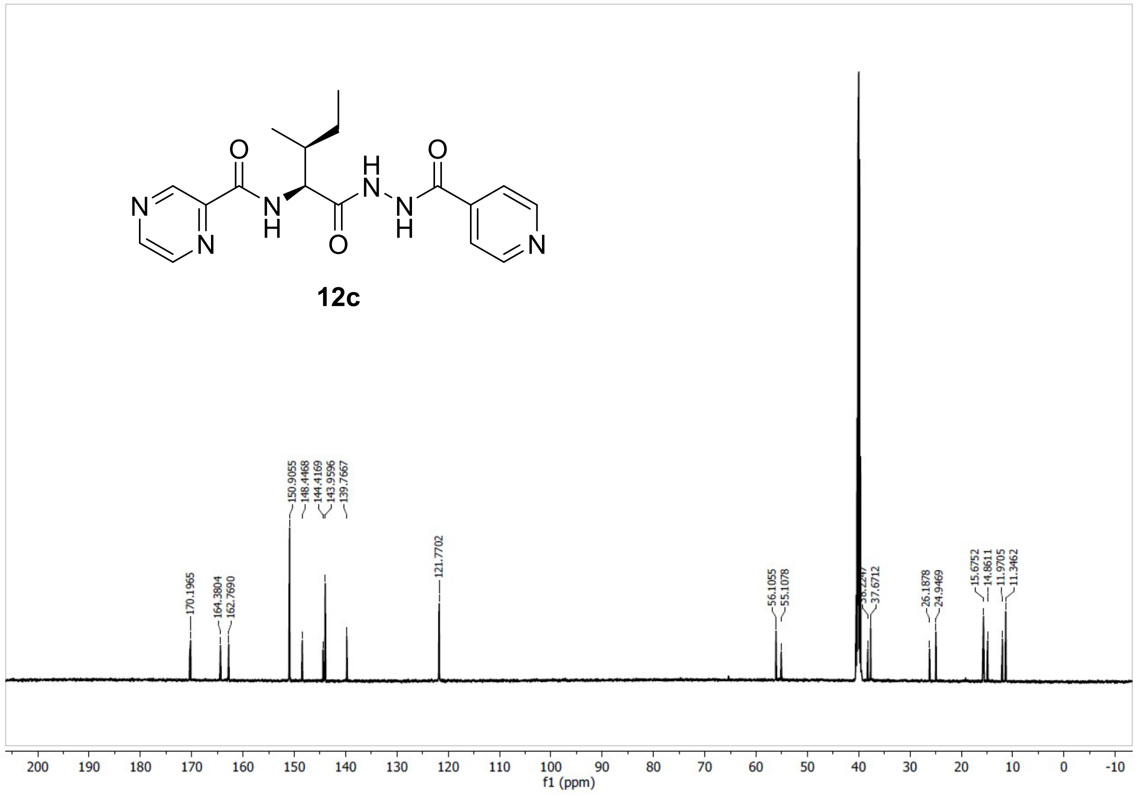

12c

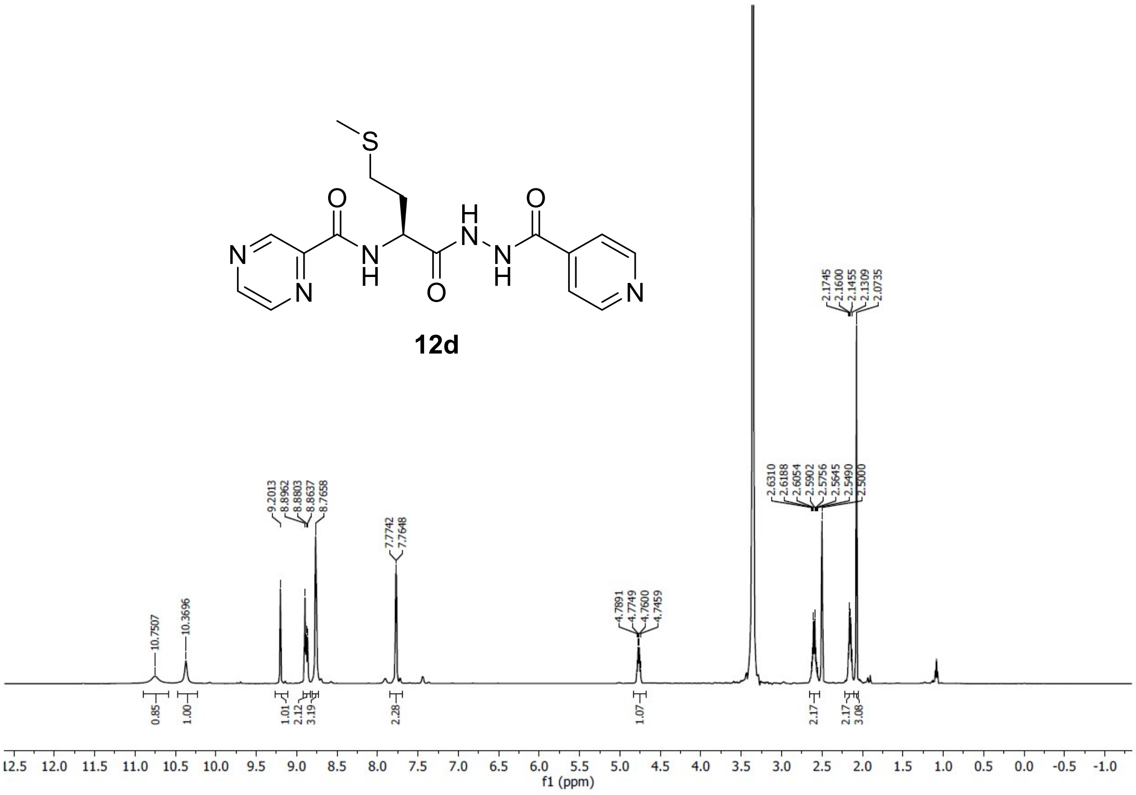
12d
10.7507
10.3696
9.2013
8.8962
8.8803
8.8637
8.7658
7.7742
7.7648
4.7891
4.7749
4.7600
4.7459
2.6310
2.6188
2.6054
2.5902
2.5756
2.5645
2.5490
2.5000
2.1745
2.1600
2.1455
2.1309
2.0735
0.85
1.00
1.01
2.12
3.19
2.28
1.07
2.17
2.17
3.08
f1 (ppm)

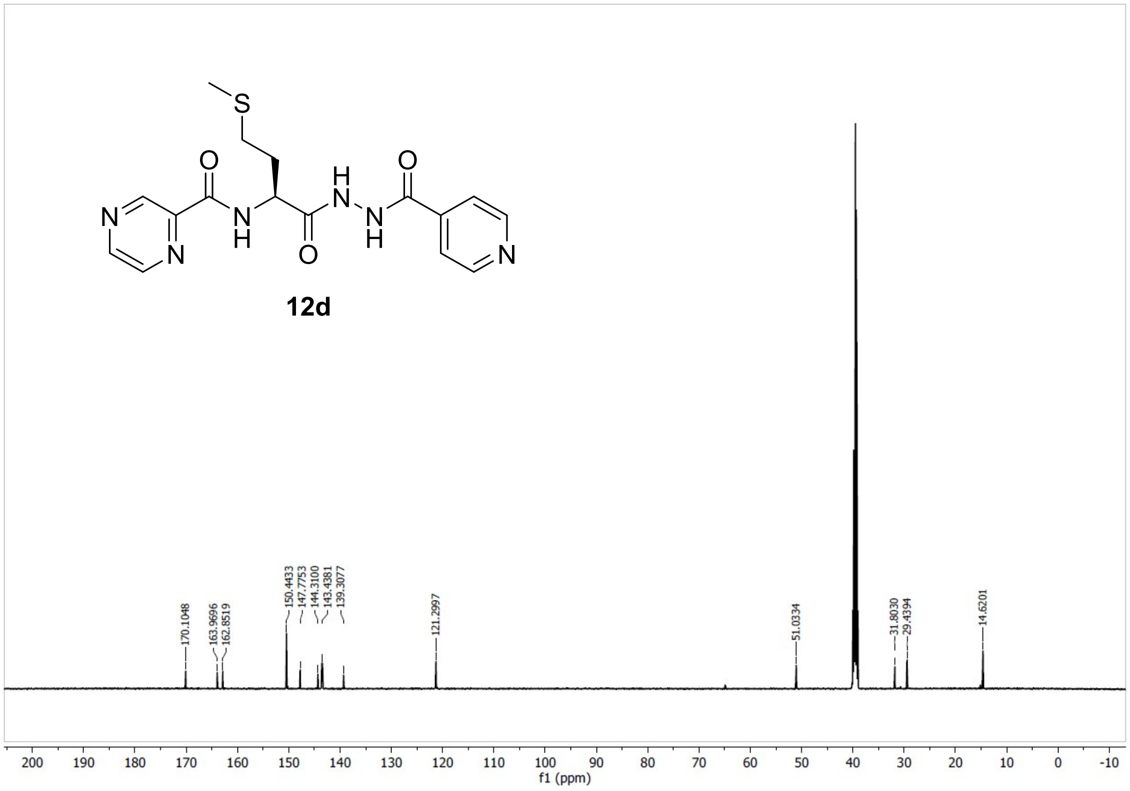
12d
170.1048
163.9696
162.8519
150.4433
147.7753
144.3100
143.4381
139.3077
121.2997
51.0334
31.8030
29.4394
14.6201
f1 (ppm)

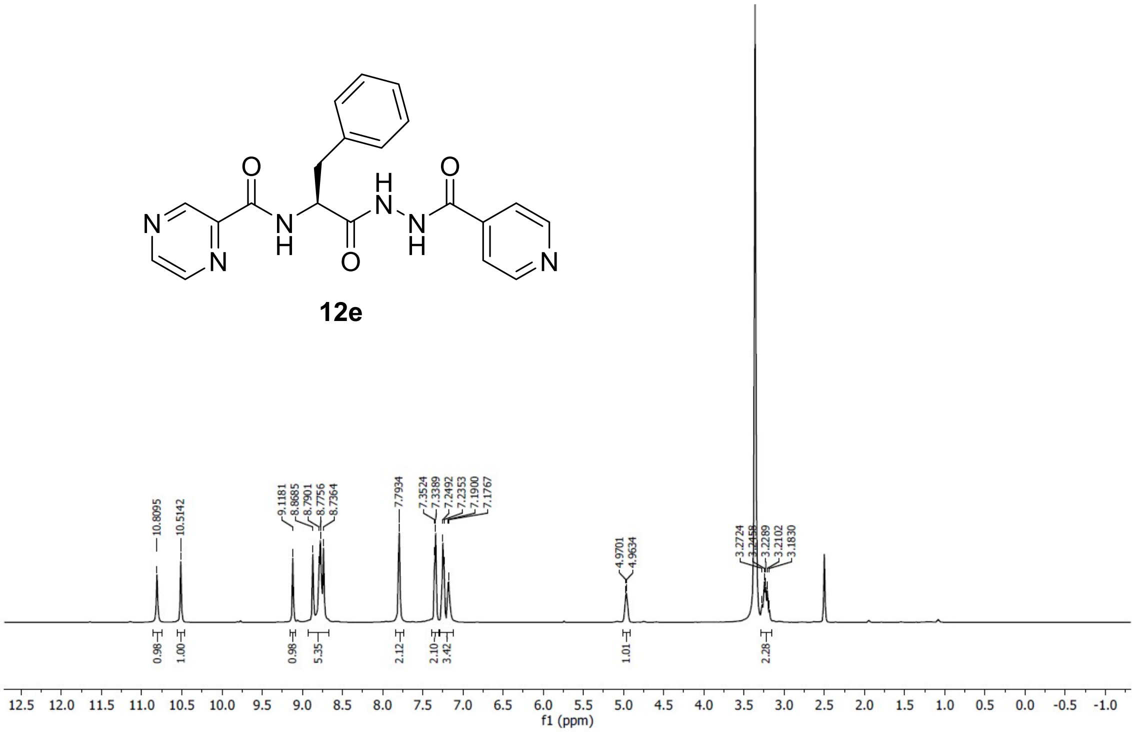
12e
10.8095
10.5142
9.1181
8.8685
8.7901
8.7756
8.7364
7.7934
7.3524
7.3389
7.2492
7.2353
7.1900
7.1767
4.9701
4.9634
3.2724
3.2458
3.2289
3.2102
3.1830
0.98
1.00
0.98
5.35
2.12
2.10
3.42
1.01
2.28
f1 (ppm)

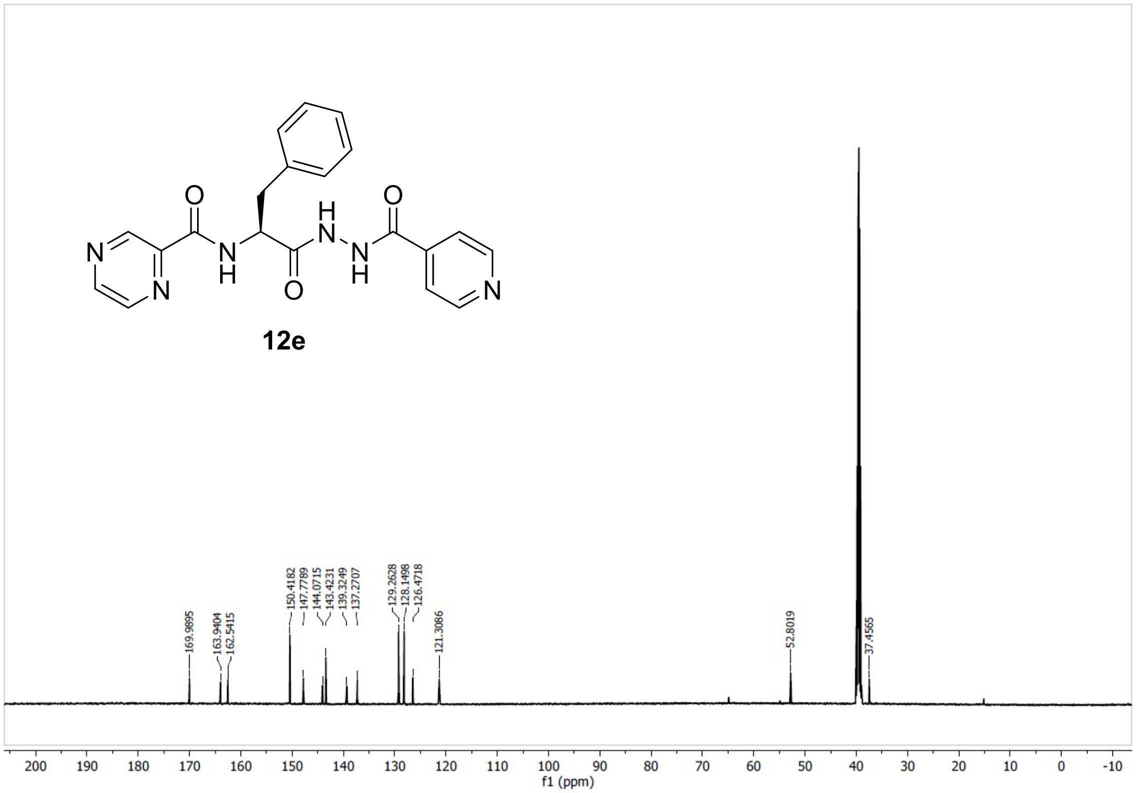
12e
169.9895
163.9404
162.5415
150.4182
147.7789
144.0715
143.4231
139.3249
137.2707
129.2628
128.1498
126.4718
121.3086
52.8019
37.4565
f1 (ppm)

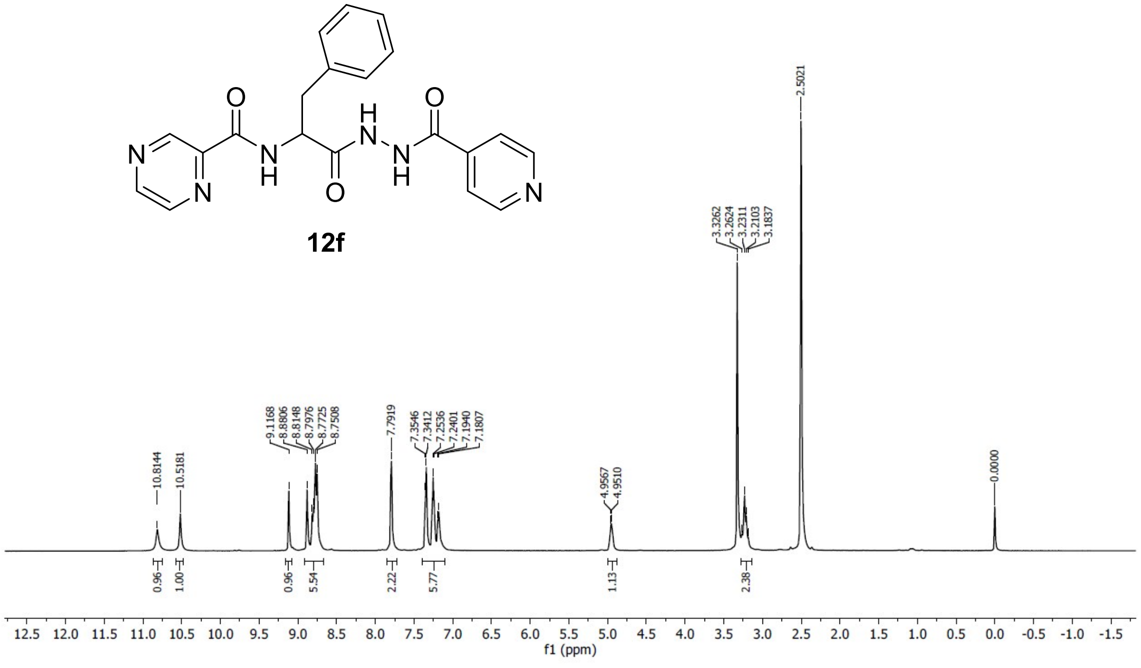
12f
10.8144
10.5181
9.1168
8.8806
8.8148
8.7976
8.7725
8.7508
7.7919
7.3546
7.3412
7.2536
7.2401
7.1940
7.1807
4.9567
4.9510
3.3262
3.2624
3.2311
3.2103
3.1837
2.5021
0.0000
0.96
1.00
0.96
5.54
2.22
5.77
1.13
2.38
f1 (ppm)

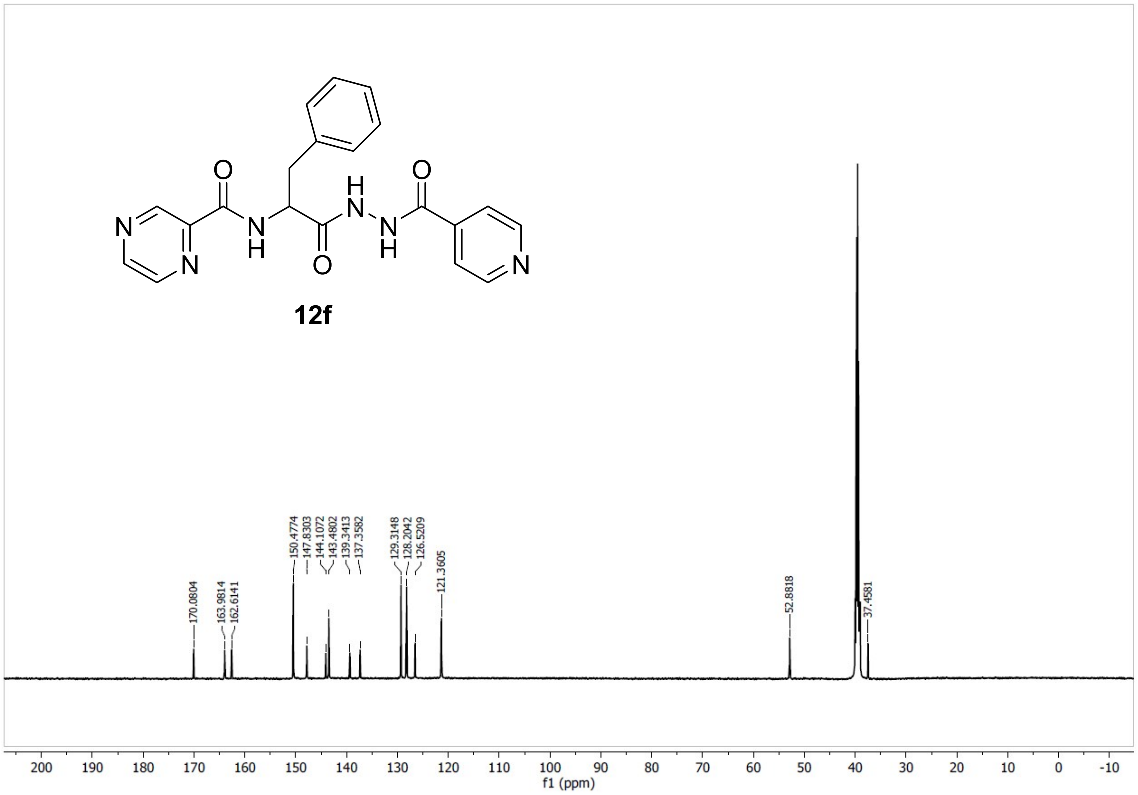
12f
170.0804
163.9814
162.6141
150.4774
147.8303
144.1072
143.4802
139.3413
137.3582
129.3148
128.2042
126.5209
121.3605
52.8818
37.4581
f1 (ppm)

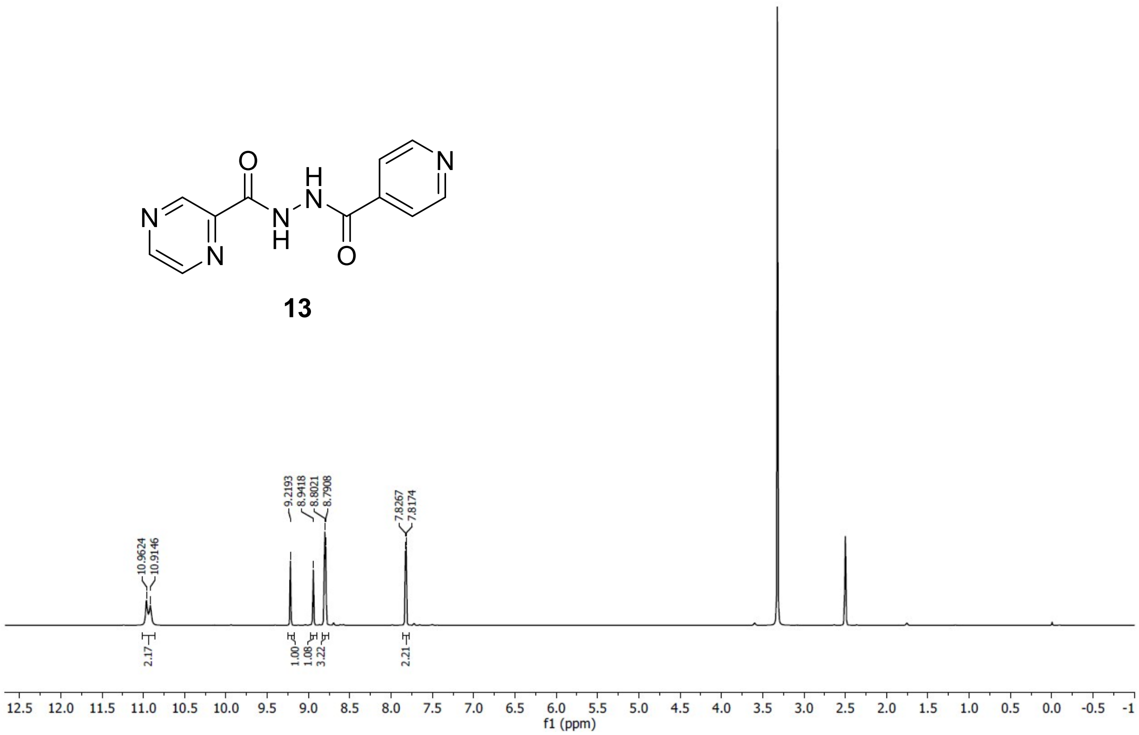
13
10.9624
10.9146
9.2193
8.9418
8.8021
8.7908
7.8267
7.8174
2.17
1.00
1.08
3.22
2.21
12.5 12.0 11.5 11.0 10.5 10.0 9.5 9.0 8.5 8.0 7.5 7.0 6.5 6.0 5.5 5.0 4.5 4.0 3.5 3.0 2.5 2.0 1.5 1.0 0.5 0.0 -0.5 -1
f1 (ppm)

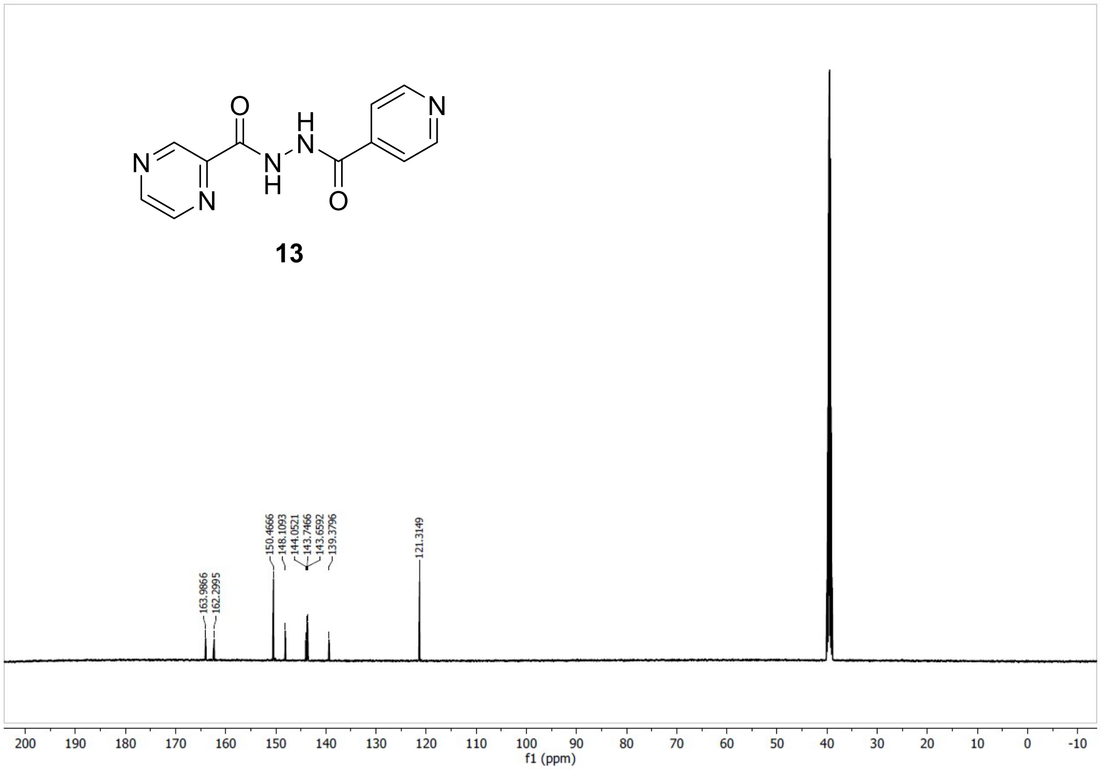
13
163.9866
162.2995
150.4666
148.1093
144.0521
143.7466
143.6592
139.3796
121.3149
200 190 180 170 160 150 140 130 120 110 100 90 80 70 60 50 40 30 20 10 0 -10
f1 (ppm)

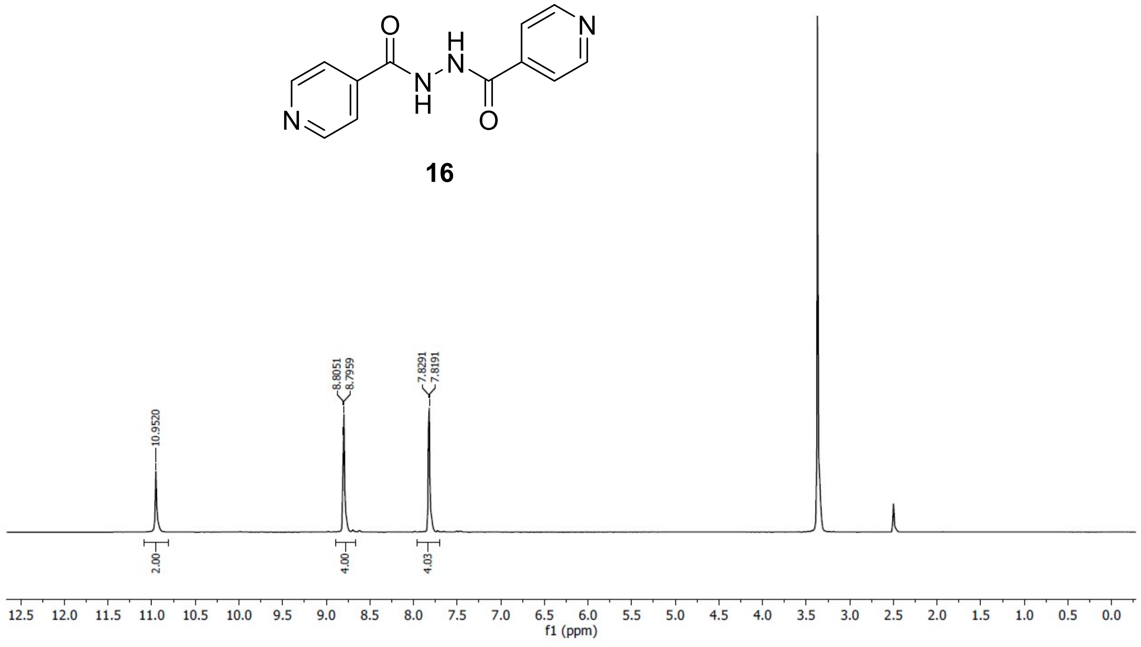
O
H
N
N
H
N
O
N
16
10.9520
8.8051
8.7959
7.8291
7.8191
2.00
4.00
4.03
12.5 12.0 11.5 11.0 10.5 10.0 9.5 9.0 8.5 8.0 7.5 7.0 6.5 6.0 5.5 5.0 4.5 4.0 3.5 3.0 2.5 2.0 1.5 1.0 0.5 0.0
f1 (ppm)

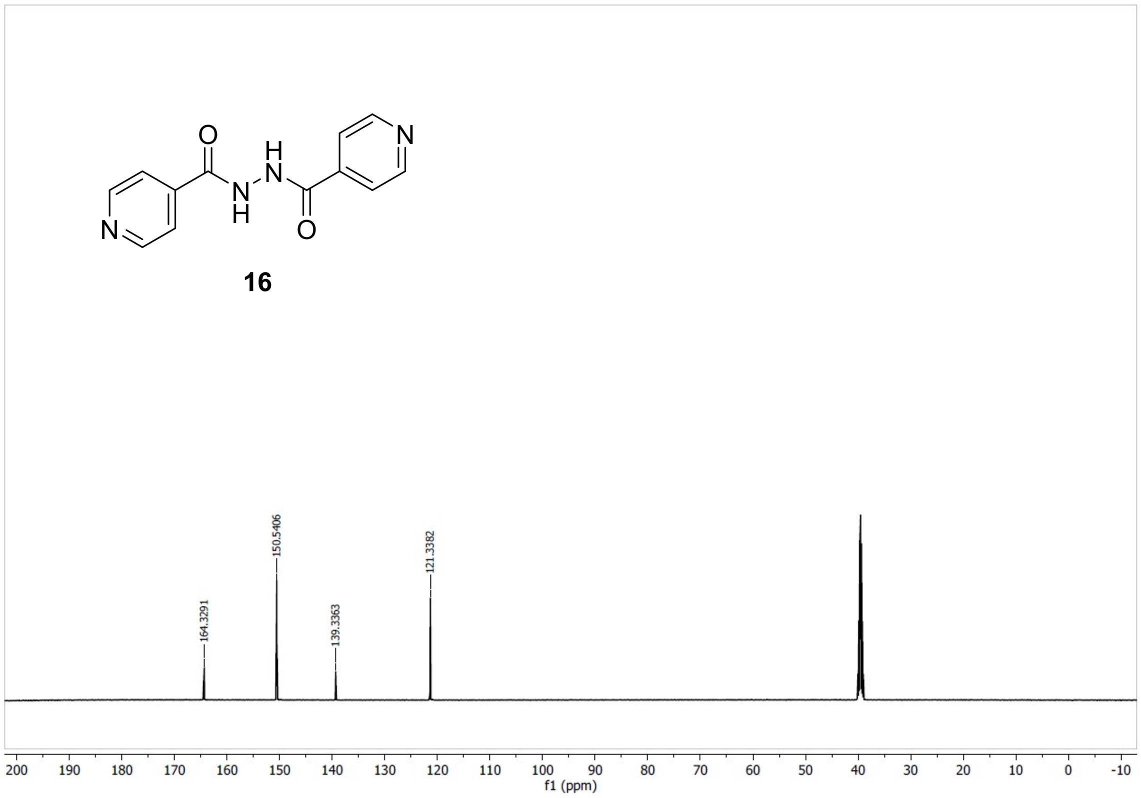
O
H
N
N
H
N
O
N
16
164.3291
150.5406
139.3363
121.3382
200 190 180 170 160 150 140 130 120 110 100 90 80 70 60 50 40 30 20 10 0 -10
f1 (ppm)

Full spectrum

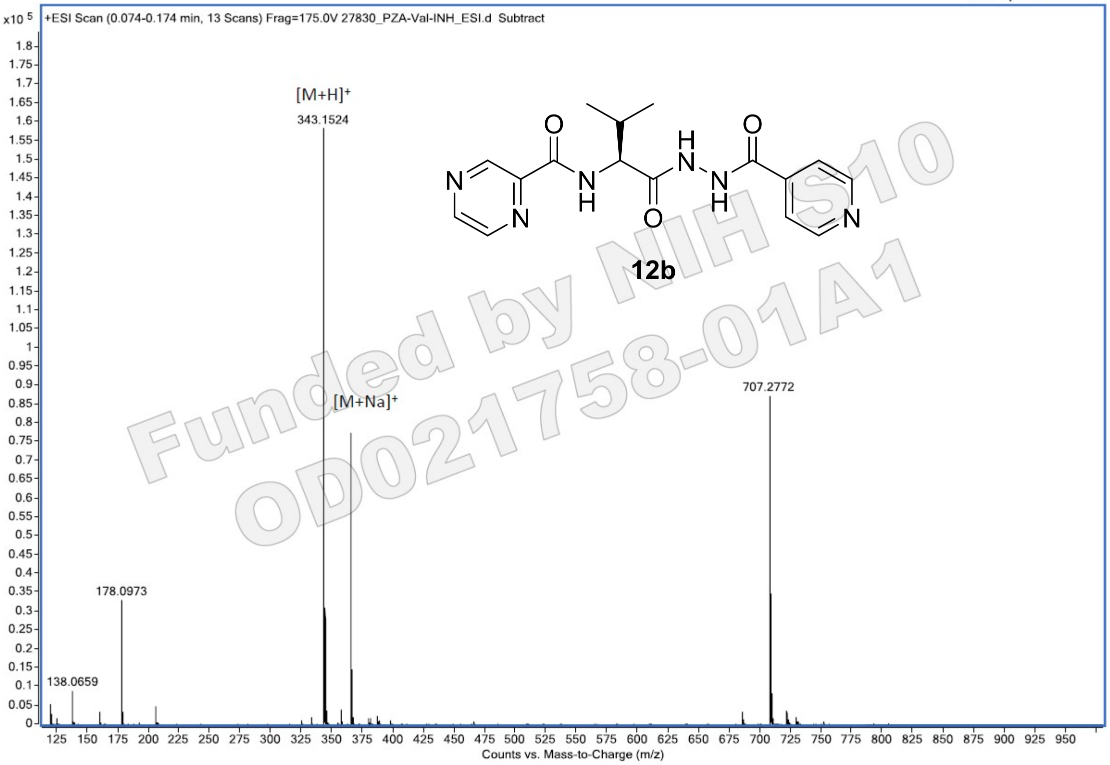

Theoretical [M+H]+ = 343.1513 (3.2 ppm)
Theoretical [M+Na]+ = 365.1335 (0.5 ppm)

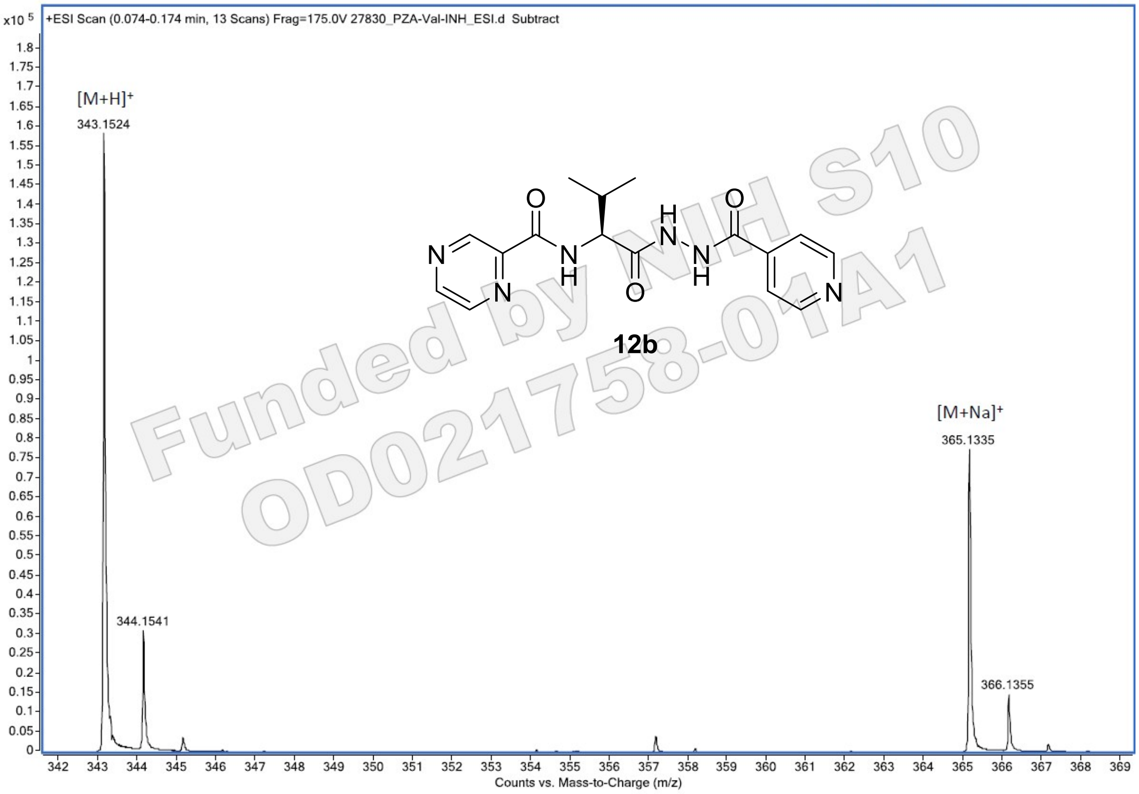

Full spectrum

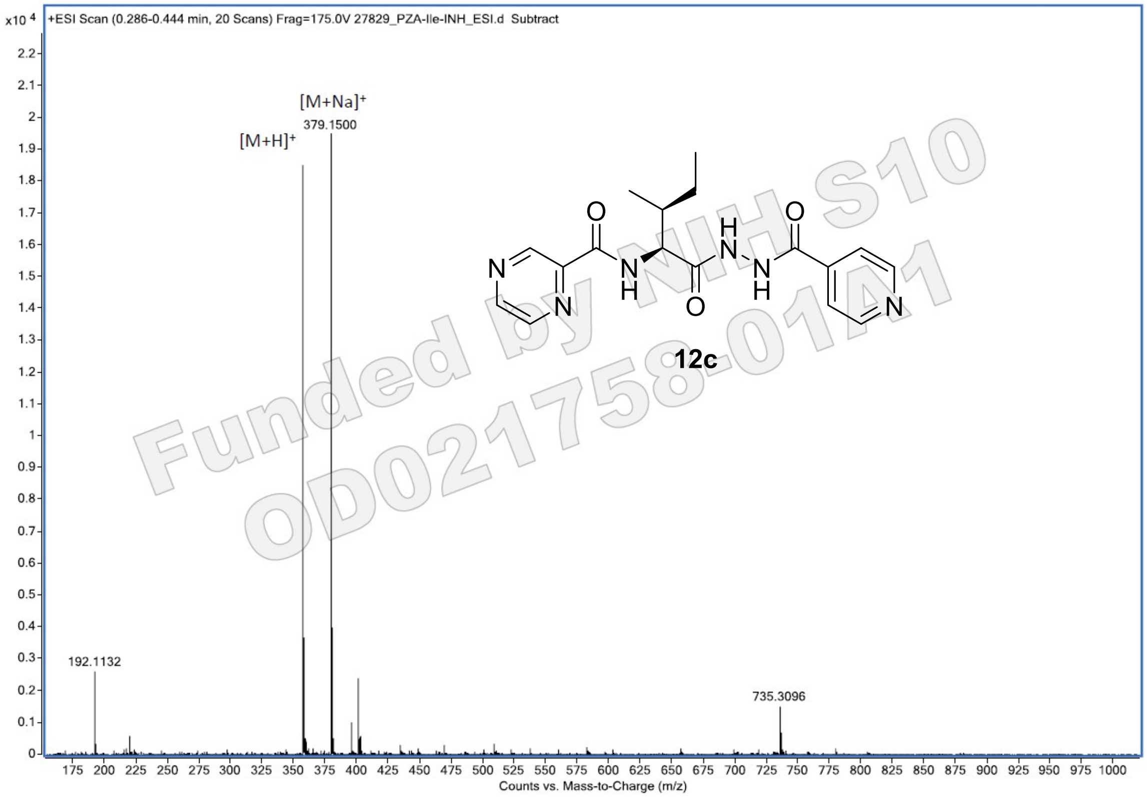

Theoretical [M+H]+ = 357.1670 (2.8 ppm)
Theoretical [M+Na]+ = 379.1489 (2.9 ppm)

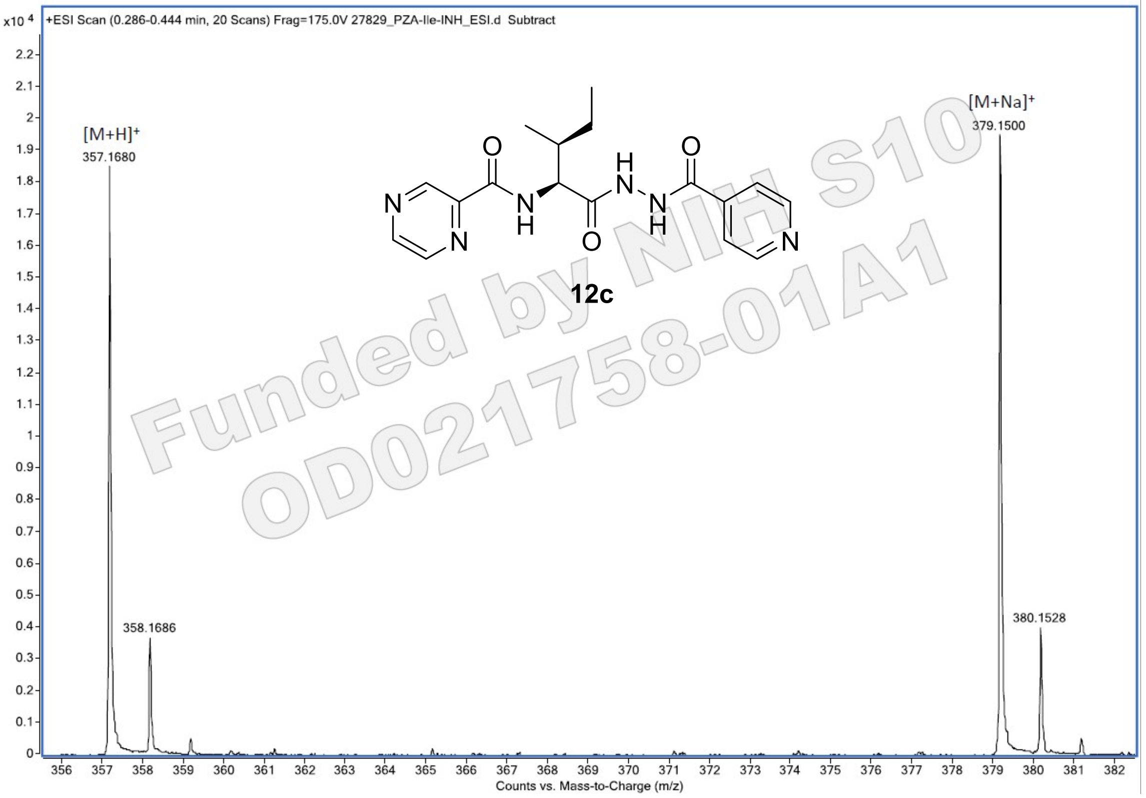

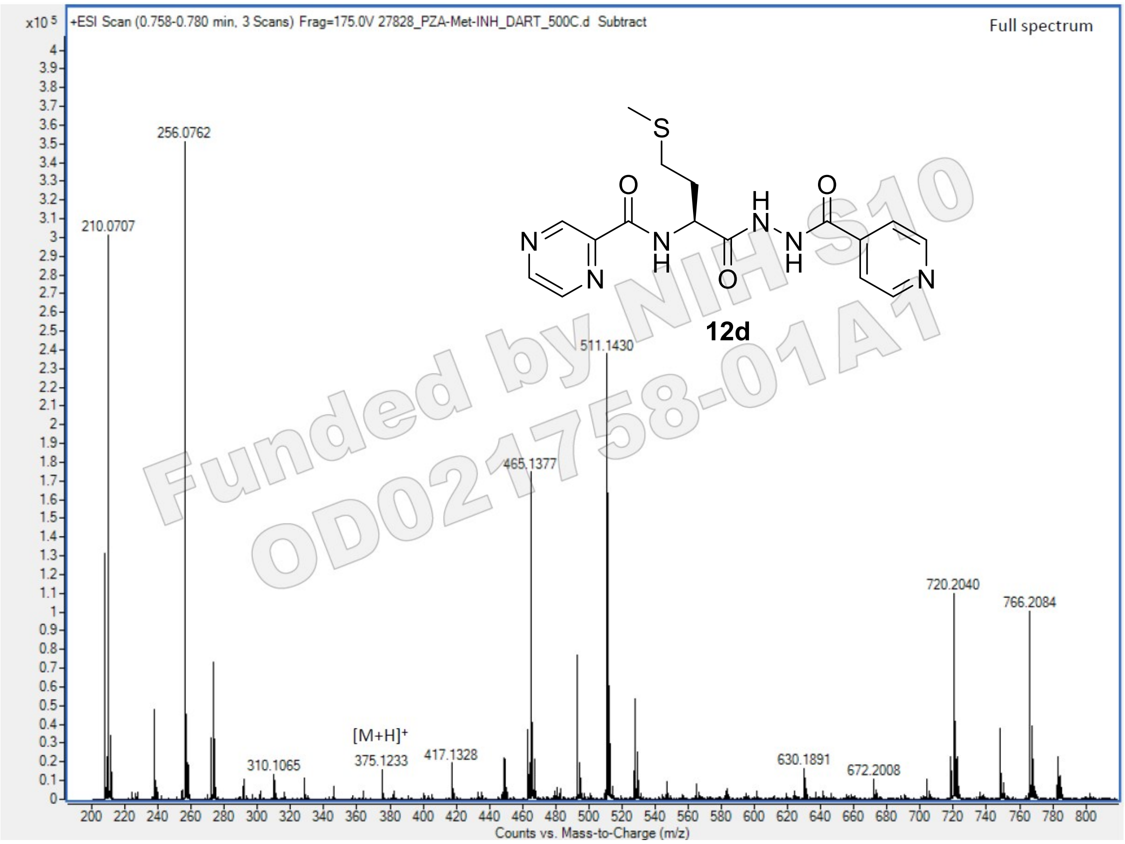

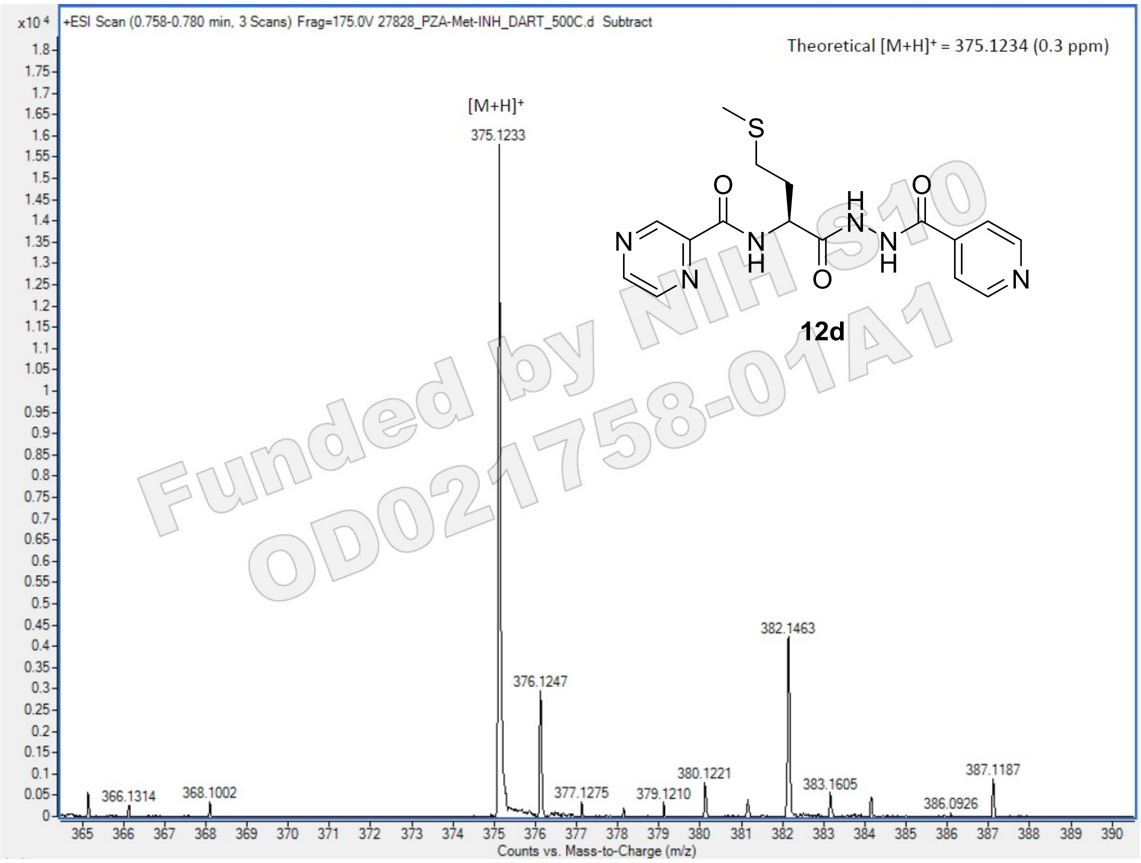

Funded by NIH S10 OD021758-01A1

Full spectrum

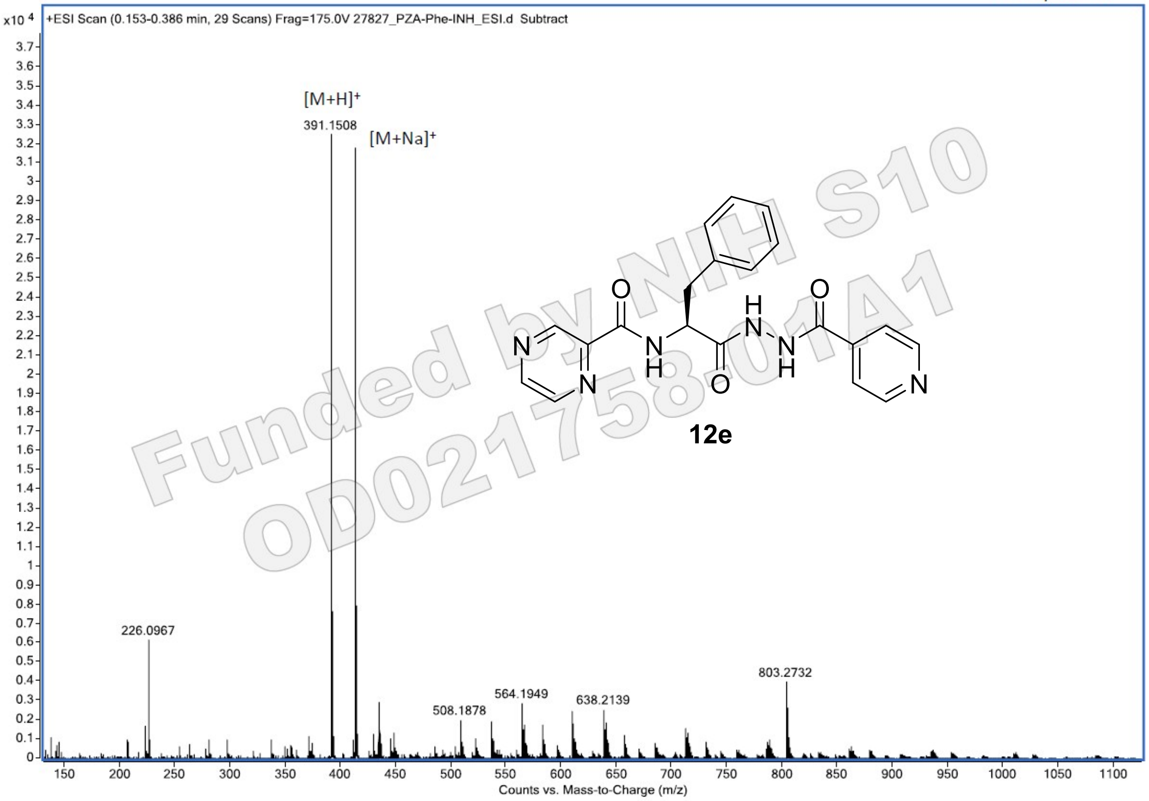

Theoretical [M+H]+ = 391.1513 (1.3 ppm)
Theoretical [M+Na]+ = 413.3333 (3.1 ppm)

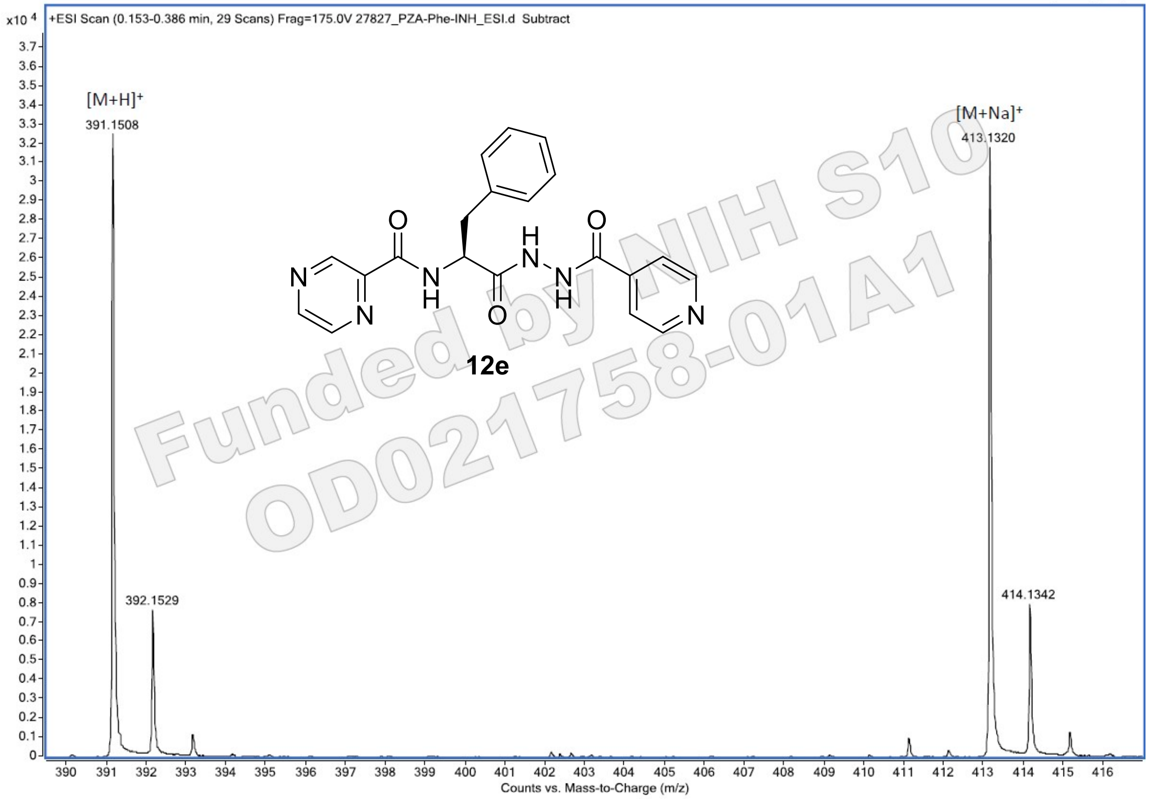

Funded by NIH S10 OD021758-01A1

Full spectrum

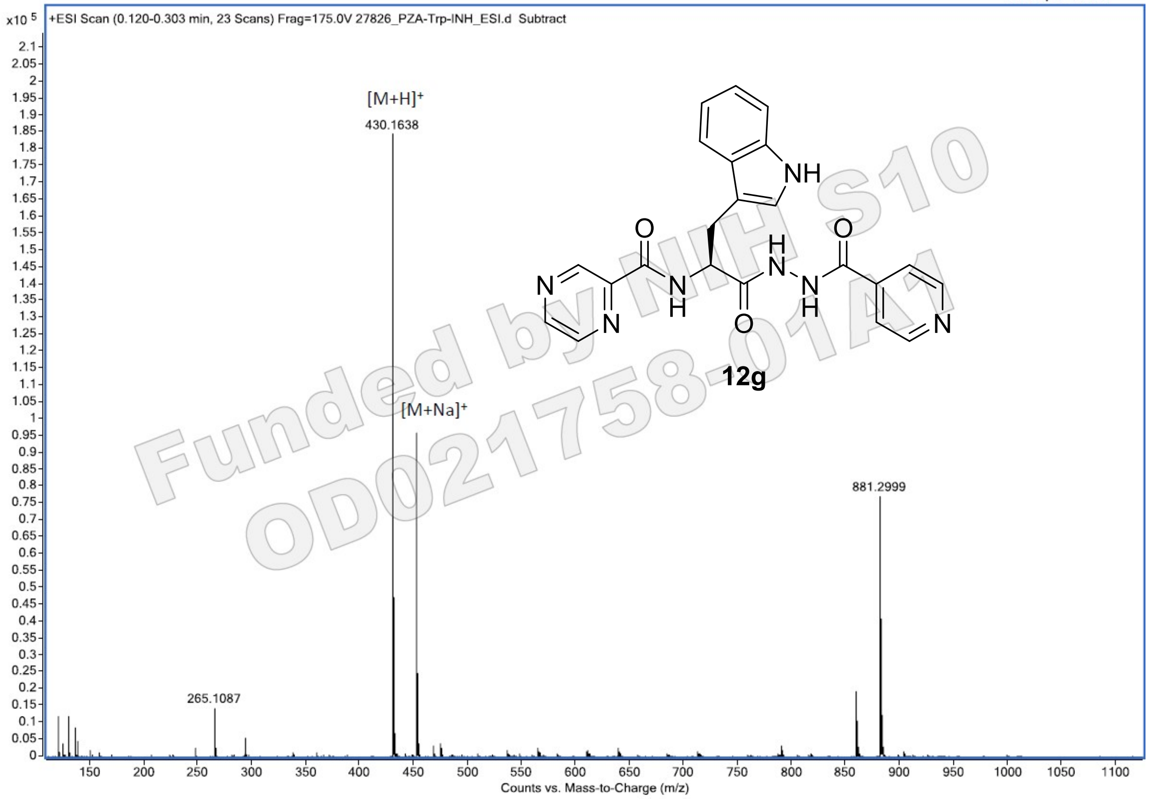

Theoretical [M+H]+ = 430.1622 (3.7 ppm)
Theoretical [M+Na]+ = 452.1442 (2.0 ppm)

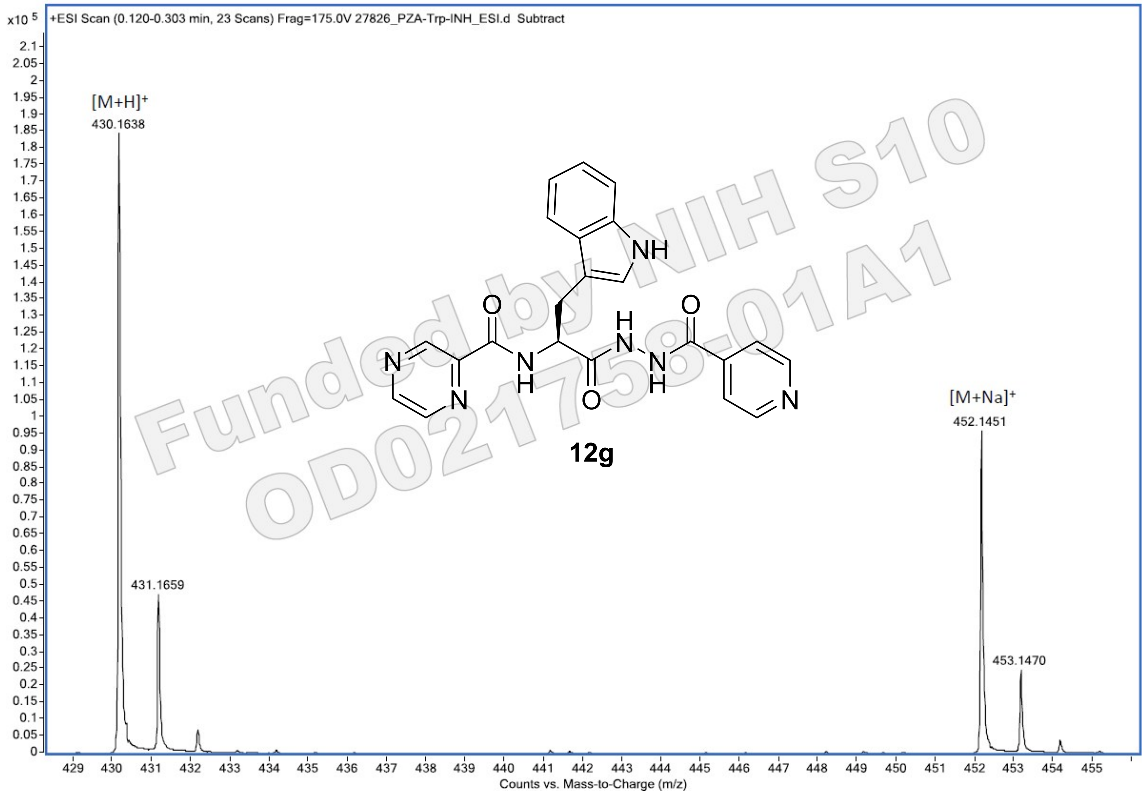

Full spectrum

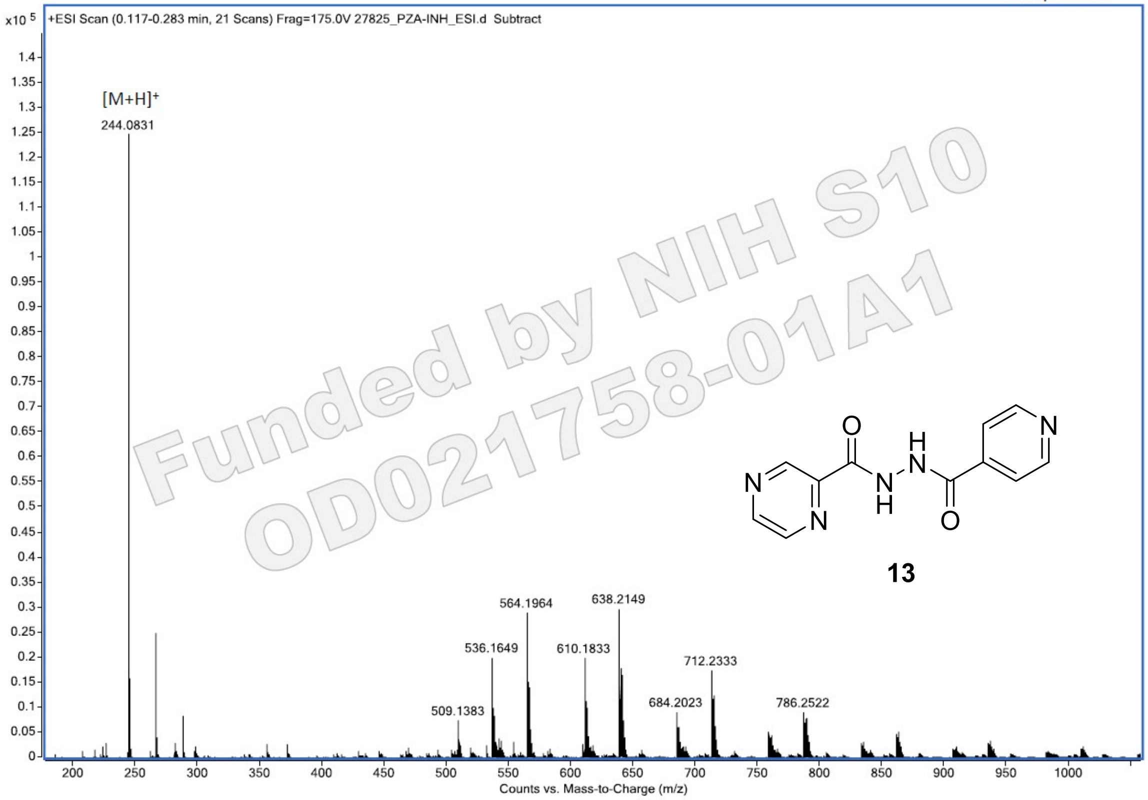

Theoretical $[M+H]^+$ = 244.0829 (0.8 ppm)

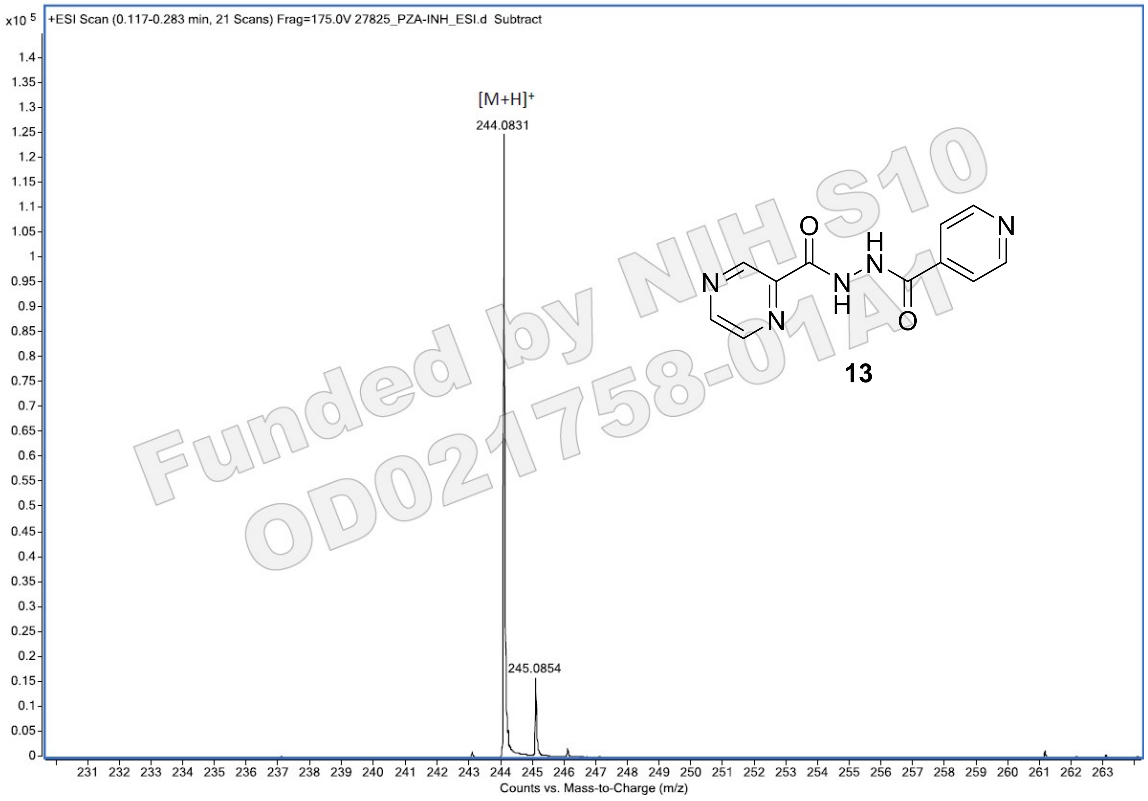

Funded by NIH S10 OD021758-01A1

Full spectrum

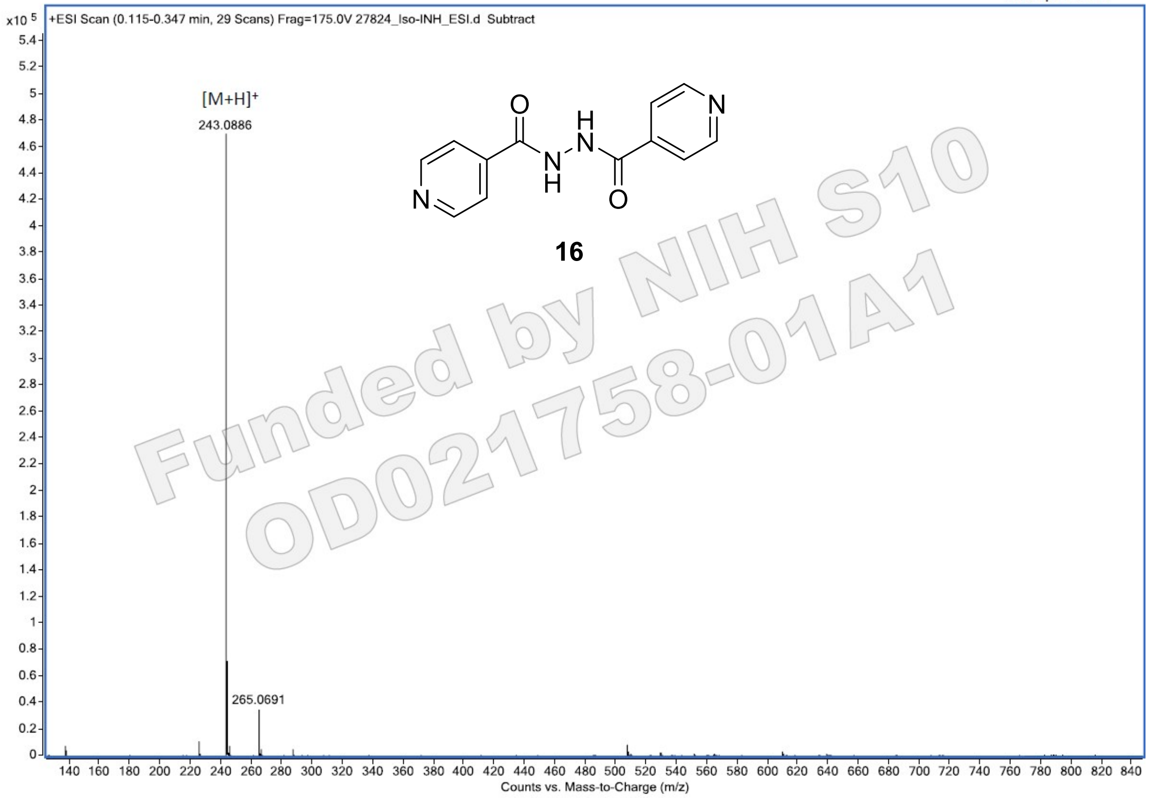

Theoretical [M+H]+ = 243.0877 (3.7 ppm)

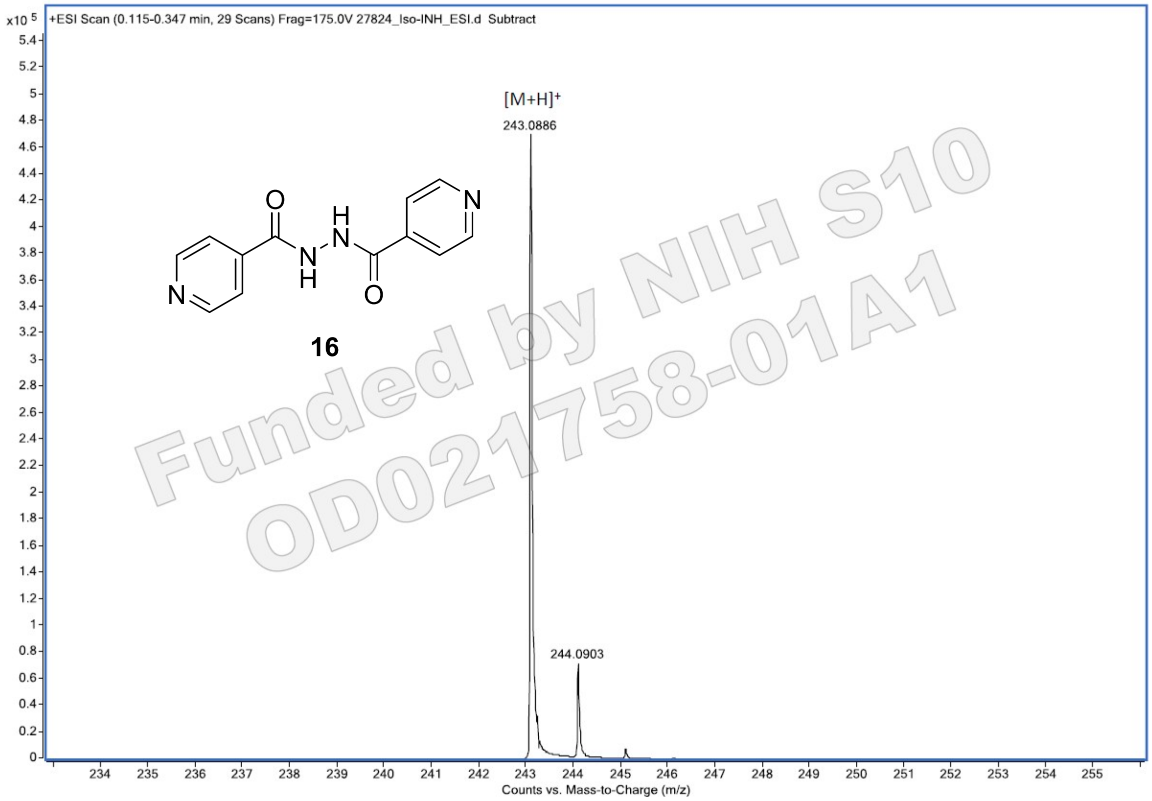

Funded by NIH S10 OD021758-01A1

HPLC spectra of compounds 12e and 12f

Instrument: Agilent 6120

Column: Chirobiotic T

Detector: UV detector

Mobile phase: Methanol

Injection volume: 5 μL

Flow rate: 0.5 mL/min

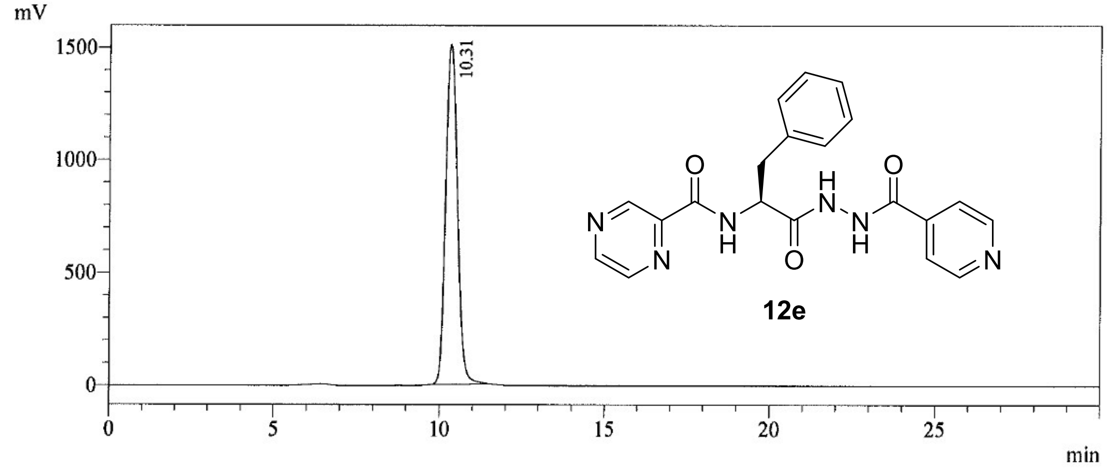

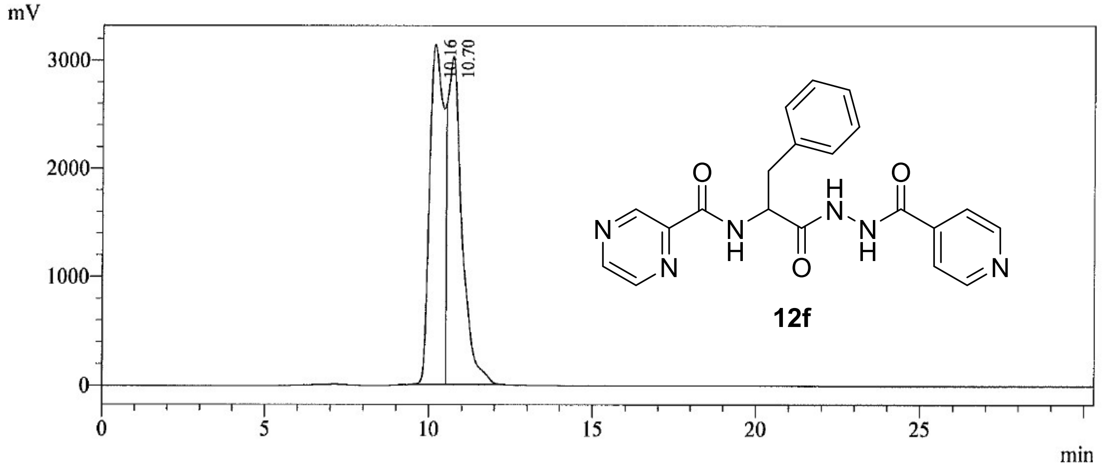

Supplement: RA-009-C9RA03380G-s001 [file RA-009-C9RA03380G-s001.pdf]
